# HacA Governs Virulence Traits and Adaptive Stress Responses in *Trichophyton rubrum*

**DOI:** 10.3389/fmicb.2020.00193

**Published:** 2020-02-20

**Authors:** Tamires A. Bitencourt, Elza A. S. Lang, Pablo R. Sanches, Nalu T. A. Peres, Vanderci M. Oliveira, Ana Lúcia Fachin, Antonio Rossi, Nilce M. Martinez-Rossi

**Affiliations:** ^1^Department of Genetics, Ribeirão Preto Medical School, University of São Paulo, São Paulo, Brazil; ^2^Department of Microbiology, Institute of Biological Sciences, Federal University of Minas Gerais, Belo Horizonte, Brazil; ^3^Department of Biotechnology, University of Ribeirão Preto, Ribeirão Preto, Brazil

**Keywords:** mycoses, secretory system, unfolded protein response, dermatophytes, endoplasmic reticulum, host-pathogen interaction

## Abstract

The ability of fungi to sense environmental stressors and appropriately respond is linked to secretory system functions. The dermatophyte infection process depends on an orchestrated signaling regulation that triggers the transcription of genes responsible for adherence and penetration of the pathogen into host-tissue. A high secretion system is activated to support the host-pathogen interaction and assures maintenance of the dermatophyte infection. The gateway of secretion machinery is the endoplasmic reticulum (ER), which is the primary site for protein folding and transport. Current studies have shown that ER stress that affects adaptive responses is primarily regulated by UPR and supports fungal pathogenicity; this has been assessed for yeasts and *Aspergillus fumigatus*, in regard to how these fungi cope with host environmental stressors. Fungal UPR consists of a transmembrane kinase sensor (Ire1/IreA) and a downstream target Hac1/HacA. The active form of Hac is achieved via non-spliceosomal intron removal promoted by endonuclease activity of Ire1/IreA. Here, we assessed features of HacA and its involvement in virulence and susceptibility in *Trichophyton rubrum*. Our results showed that exposure to antifungals and ER-stressing agents initiated the activation of HacA from *T. rubrum*. Interestingly, the activation occurs when a 20 nt fragment is removed from part of the exon-2 and part of intron-2, which in turn promotes the arisen of the DNA binding site motif and a dimer interface domain. Further, we found changes in the cell wall and cellular membrane composition in the Δ*hacA* mutant as well as an increase in susceptibility toward azole and cell wall disturbing agents. Moreover, the Δ*hacA* mutant presented significant defects in important virulence traits like thermotolerance and growth on keratin substrates. For instance, the development of the Δ*hacA* mutant was impaired in co-culture with keratinocytes or human nail fragments. Changes in the pro-inflammatory cytokine release were verified for the Δ*hacA* mutant during the co-culture assay, which might be related to differences in pathogen-associated molecular patterns (PAMPs) in the cell wall. Together, these results suggested that HacA is an integral part of *T. rubrum* physiology and virulence, implying that it is an important molecular target for antidermatophytic therapy.

## Introduction

The superficial infections of the skin and nails represent the most common human mycoses, and it is estimated to affect about 1.7 billion of the population (Brown et al., 2012). These infections are mainly caused by dermatophytes, in which *Trichophyton rubrum* followed by *Trichophyton interdigitale* have been described as the predominant species isolated in dermatophytosis cases worldwide (Almeida et al., 2019).

During the dermatophyte-host interaction, a complex signaling network enables infection establishment. A profound metabolic change is required to overcome the hostile host environment, in which fungi cope with acidic skin pH, shortages of nutrients, skin desquamation, the action of phagocytic cells, and antimicrobial peptides (Leal et al., 2009; Martinez-Rossi et al., 2016b). Besides, a highly efficient secretion system is triggered to support the host attachment and nutrient acquisition by the pathogen (Martinez-Rossi et al., 2016b; Mercer and Stewart, 2019).

The endoplasmic reticulum (ER) represents the gateway of the secretory pathway, where most of the plasma membrane and secreted proteins undergo proper folding and post-translation modifications (Schwarz and Blower, 2016). The secretory system is used by different pathogens to express virulence factors, and to cope with stress conditions, which ultimately might favor their adaptation to specific biological niches (Krishnan and Askew, 2014).

When the ER capacity is overwhelmed by high concentrations of proteins inside the milieu, which exceed the ER folding competence, there is an accumulation of misfolded proteins that compromise cellular physiology. In order to mitigate this ensuing status of ER stress, a series of adaptive responses collectively termed unfolded protein responses (UPR) is initiated (Moore and Hollien, 2012). The UPR pathway is activated to restore ER homeostasis by enhancing the folding ability and controlling misfolded proteins disposal. In fungi, UPR consists of an ER-transmembrane sensor Ire1/IreA (Ser/Thr kinase) with an endonuclease domain, and the transcription factor Hac1/HacA. Upon ER stress, the IreA is activated and cleaves in a non-canonical way to the Hac mRNA. The splicing sites are recognized through a conserved RNA secondary structure that flanks the cleavage sites. The splicing of cytosolic Hac1/HacA mRNA shifts the open reading frame, and the spliced form is translated to a potent bZIP transcription factor that is transported to the nucleus, where it regulates the UPR target genes (Saloheimo et al., 2003).

The UPR has been reported to be a therapeutic vulnerability target in pathogenic fungi. It is assumed to be an essential regulator of *Aspergillus fumigatus* pathogenicity (Feng et al., 2011; Richie et al., 2011). Moreover, in *Cryptococcus neoformans* and *Candida albicans*, UPR affected virulence traits as deletions in these genes were associated with impairment of the ability to switch from yeast to hyphal form, compromised cell wall adhesive proteins, and reduction of thermotolerance (Matsumoto et al., 2005; Wimalasena et al., 2008; Blankenship et al., 2010; Krishnan and Askew, 2014).

Notably, consistent divergences of domain structure from mammalian Hac1/HacA ortholog, termed Xbp1, denote HacA as an attractive target for antifungal therapy (Zhang et al., 2016). Further, the current paradigm is that ER-stress response pathways are involved in the expression of different virulence traits that may be necessary for pathogen-host interaction (Krishnan and Askew, 2014). Thus, we have addressed the scope of HacA in the dermatophyte *Trichophyton rubrum*. Here, we characterized HacA from *T. rubrum* and confirmed its involvement in *T. rubrum* pathogenicity and adaptive responses to different stressors, and the immune modulation of keratinocyte cells.

## Materials and Methods

### Strain and Culture Conditions

*Trichophyton rubrum* CBS118892 strain (Centraalbureau voor Schimmelcultures, Fungal Biodiversity Centre, Netherlands) was cultivated in malt extract at 28°C, as previously described (Peres et al., 2016). Conidia suspension was obtained from 20-days-old plates, and the concentration was estimated by using a Newbauer chamber. Approximately 1 × 10^6^ conidia were added in 50 mL of liquid Sabouraud followed by incubation at 28°C for 96 h under continuous shaking. The resulting mycelia were then transferred to 100 mL of Sabouraud in the presence of sublethal doses of acriflavine (ACR), caspofungin (CASP), griseofulvin (GRS), terbinafine (TRB), or undecanoic acid (UDA), and in the absence of drugs (control), followed by incubation at 28°C with shaking (120 rpm) for 12 h. The concentrations used for each drug were 70% of their minimum inhibitory concentration values, and obtained in accordance with the CLSI (M-38A2) (Clinical Laboratory Standards Institute [CLSI], 2008) with the following modifications, in Sabouraud media corresponding to 5.46 μg/mL for ACR, 87.5 μg/mL for CASP, 2.76 μg/mL for GRS, 0.014 μg/mL for TRB, and 35 μg/mL for UDA. Dithiothreitol (DTT) and tunicamycin (TUN), in concentrations of 10 mM and 21.87 μg/mL, respectively, were used as a positive control for the UPR response. The cytotoxic compounds were purchased from Sigma-Aldrich (St. Louis, MO, United States), with the exception of CASP, which was purchased from Merck (Kenilworth, NJ, United States).

### Total RNA Extraction and cDNA Synthesis

The mycelia were ground by mechanical pulverization with a pestle and mortar in liquid nitrogen, and total RNA isolation was carried out using TRIzol (Thermo Fisher Scientific, Carlsbad, CA, United States) following the manufacturer’s instructions. RNA samples were treated with RNase-free DNAse I (Sigma-Aldrich). Complementary DNA was synthesized from each condition containing 1000 ng of total RNA in a 20 μL reaction volume using a High Capacity cDNA Synthesis kit (Thermo Fisher Scientific #4368814).

### RT-PCR and qRT-PCR

For qualitative expression analysis, primer pairs that yielded PCR products around the predicted *hacA* excision region (Table 1) were used to amplify the two isoform products of the gene coding for HacA in *T. rubrum* (TERG_05396). For the PCR reaction, approximately 140 ng of cDNA and 0.2 pmol/μL of each oligonucleotide were used. Thermocycler conditions were 95°C for 2 min, followed by 35 cycles at 95°C for 30 s, 53°C for 45 s, and 72°C for 1 min, and the last cycle at 72°C for 10 min.

**TABLE 1:** Set of primers used in RT-PCR and qRT-PCR.

**ID**	**Gene symbol**	**Sequence 5′–3′**	**Concentration (nM)**	**Size (bp)**	**References**
TERG_05717	*erg1*	F:GTGAAGATACCTTTCCCTAGCG	100	148	Komoto et al., 2015
		R: TTATGGTAGAAACGGCCTTGG			
TERG_01127	*fks1*	F: CGTGGTGGTGATGGTGATTA	100	111	This work
		R: GTAGGAGATCTGAGAGGATGGA			
TERG_01883	*hsp75*-like	F: GTCTACTGAAACTTACGACG	300	87	Neves-Da-Rocha et al., 2019
		R: TCAACGTTGGCGCCCTCATA			
TERG_05742	*rpb2*	F: TGCAGGAGCTGGTGGAAGA	300	59	Jacob et al., 2012
		R: GCTGGGAGGTACTGTTTGATCAA			
TERG_06963	*hsp90*	F: ACCGTGCTGCCCTTGCT	300	61	Jacob et al., 2015
		R: GTGATCTCGTCGCCAGACTTG			
TERG_06338	*N-man*	F: TAAACGACAGTGGTATGCCG	300	203	Mendes et al., 2012
		R: TGTAGCCTGTTGGGTTCTCT			
TERG_06465	O-man	F: CCATGGGACGTGTATACTC	300	129	Mendes et al., 2012
		R: CGTCATCATAGCAACATTCAG			
TERG_01292	Alpha-man	F: CCTACTACACCGGAAATCACAC	300	119	This work
		R: GTCGCCAGTATACCACCAATAG			
TERG_07657	*Chsd*	F: AGCAGTGTGCCGATCTATTC	300	91	This work
		R: CTGTGCCTAGCTCCAATCAT			
TERG_02850	*pksP*	F: CTTTGTGGCAGCGTGATATTG	100	85	This work
		R: CGATCCAGACCAGCAGTAAAG			
TERG_05396	*hacA*	F: TCTCACCGGCTGACTTGGAT	–	253/233	This work
		R: CCCGTCTTCAAGGAATGA			
TERG_07904	β*-tub*	F: CGGTATGATGGCCACTTTCT	–	315	This work
		R: CTGACCTGGGAAACGAAGAC			

The qPCR assays were performed using SYBR green PCR master mix (Applied Biosystems), 70 ng cDNA, forward and reverse primers used in concentrations previously determined (Table 1) in a 12.5 μL reaction mixture. The *rpb2* gene was used as a reference control, and all the analyses were carried out as previously described (Jacob et al., 2012). A set of genes potentially regulated by HacA was analyzed.

### Identification and Characterization of *T. rubrum hacA*

A Blastx search using the *S. cerevisiae hac1* sequence as a query identified TERG_05396 as a putative ortholog for this gene. Thereafter, the prediction of the non-canonical intron excision site was determined by Infernal software (Nawrocki et al., 2009). From a multiple alignment file of *hac*A RNAs in the Stockholm format, a covariance model was established. From this model, screening of the *T. rubrum* genome was carried out and resulted in a region of 62 nt corresponding to fractions of exon-2 and intron-2. Next, oligonucleotides surrounding this predicted region were used for products amplification of cDNAs from *T. rubrum* following exposure to antifungal and ER stress compounds, and the sequencing of these products showed the intron removed from *T. rubrum hacA* mRNA.

### *hacA* Gene Deletion

The inactivation gene cassette was obtained using a split-marker approach (Kuck and Hoff, 2010). The 5′ UTR and 3′ UTR were fused with parts of a hygromycin resistance gene (*hph*) from pCSN43. The Overlap PCR was used to join the PCR products. Sets of primers were used for the split-marker approach, as described in Supplementary Table S1.

The protoplasts transformation was carried out as previously described (Fachin et al., 2006). The transformants were selected in Cove’s medium with 1 M sucrose and 500 μg/mL hygromycin. PCR screened the prominent colonies through analysis of amplification of the *hacA* gene. Thereafter, the loss of *hacA* gene and integration of *hph* gene were confirmed by PCR and Southern blot analysis (Supplementary Figure S1).

### Biochemical Assays

The quantification of ergosterol content was carried out as previously described (Arthington-Skaggs et al., 1999) with modifications. Mycelia of wild type and Δ*hacA* were inoculated in 50 mL liquid Sabouraud medium and incubated at 28°C for 24 h under shaking (200 rpm). The mycelia were then harvested and transferred to 20 mL fresh liquid Sabouraud medium and incubated for an extra 48 h under the same conditions described above. Further, the biomass was harvested through vacuum filtration and dried under sterile filter papers. The dried mycelium was weighed prior to saponification. Ergosterol was extracted as previously described (Arthington-Skaggs et al., 1999) and quantified spectrophotometrically based on a standard curve of different concentrations of ergosterol (Sigma-Aldrich #45480). Values were presented as μg ergosterol per g dry weight.

The keratinolytic activity was determined, as previously described (Gradisar et al., 2005). Keratin was used as a substrate (pH 8.0), and 1.0 mL of the culture supernatant was utilized as the enzyme. Conidia suspension (5 × 10^5^) of each strain, wild type and Δ*hacA* was inoculated into 25 mL of water that contained keratin (2.5 g/L) at pH 5.0, as the sole carbon and nitrogen sources, and incubated at 28°C for 7 days under shaking conditions (120 rpm). The mycelia were then harvested, and the supernatant was collected. The mycelial dry weight was obtained, and the pH of the supernatant was determined. Thereafter, the keratinolytic activity was estimated and expressed as units per gram of dry weight.

### Phenotypic Assays

The analysis of growth rates was performed in different culture media: (i) agar malt extract (MEA), pH 5.7, (ii) potato dextrose agar (PDA), pH 5.7, (iii) Sabouraud, pH 5.7, (iv) MM (Cove’s) containing nitrate (70 mM) and glucose (55 mM) pH 5.0 (Cove, 1966), and (v) MMK corresponding to MM supplemented with 5% of powder keratin, pH 5.0. A mycelium plug (0.8 cm) was inoculated into the plate center and incubated for 9 days at 28°C.

A microculture of strains was also carried out to assess differences in hyphal formation. The microculture was performed in Sabouraud agar and incubated for 6 days at 28°C.

### Susceptibility and Thermotolerance Assays

A serial drop dilution assay was performed to analyze the susceptibility of strains toward antifungals and an ER stress compound DTT. Plates were inoculated with different concentrations of a conidia suspension in the range of 10^6^–10^2^ cell/mL. After 7 days of incubation at 28°C, images were taken using Image J software (Schneider et al., 2012). The compounds assessed were: DTT (5 and 10 mM), ketoconazole – KTC (3.90, 1.95 and 0.98 μg/mL), GRS (0.98 μg/mL) and TRB (0.005 μg/mL). The antifungal compounds were used as sublethal doses (1/4 of MIC value for GRS and TRB, and 1/2, 1/4 and 1/8 of MIC value for KTC), as preliminarily determined through microdilution assays in *T. rubrum* (data not shown).

In order to evaluate the susceptibility toward compounds that act on cell wall, a conidia suspension (approximately 1 × 10^5^) was inoculated into the center of each well of a multi-well plate containing Sabouraud agar supplemented with concentrations of calcofluor white – CFW (in the range of 0–80 μg/mL) or CASP (in the range of 0–200 μg/mL) and incubated for 7 days at 28°C.

The tolerance to thermal stress conditions was also evaluated. In this sense, the number of colonies grown (CFU) after exposure to conidia (10^4^ cells and 10^3^ cells) at different temperatures (37 and 42°C) for 30 and 60 min were determined.

### Coculture and Nail Infection

Nail interaction assay was performed as previously described (Peres et al., 2016) with minor modifications. Human nail fragments (approximately 25 cm^2^) were sterilized by autoclaving. Nail fragments were then soaked with conidia (1 × 10^4^/mL) from *T. rubrum* strains for 1 h, followed by the addition of 200 μL of distilled water. The plates were incubated at 28°C for 72 h, and fungal growth was assessed by light microscopy (Leica DMI3000B). The human keratinocytes (HaCaT) cell line was cultured, as previously described (Komoto et al., 2015). The coculture assay was performed using 2 × 10^6^ conidia/mL and 2.5 10^5^ keratinocyte cells/mL. The coculture was incubated for 24 h at 37°C in 5% CO_2_. HaCaT and conidia cells were grown on RPMI medium used as controls for qPCR and cytokine evaluation assays. The cytokine levels in the supernatant cells were determined by Elisa (Peprotech, NJ, United States) according to the manufacturer’s recommendation. Committee of Ethics in Human Research of the Ribeirão Preto Medical School at the University of São Paulo approved all experiments involving the use of human nail fragments provided by healthy adults (protocol No.8330/2009).

## Results

### Identification and Characterization of *T. rubrum hacA*

**FIGURE 1:**
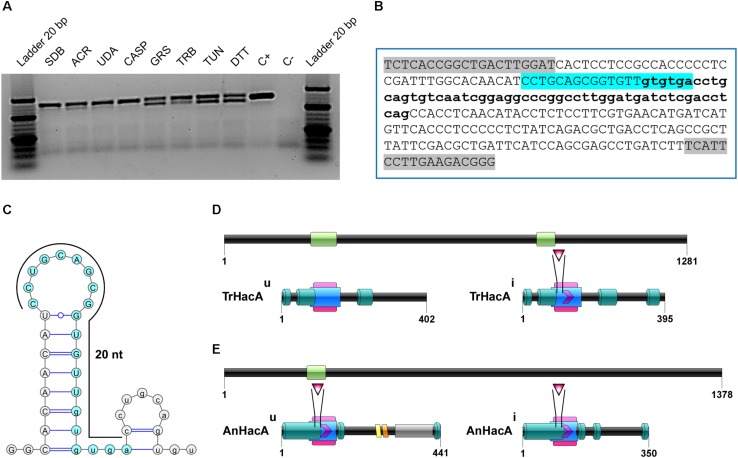
The *hac*A from *T. rubrum* is processed under antifungals exposure and ER stress-inducing agents. **(A)** RT-PCR analysis of *hac*A mRNA was used to detect the processing under antifungals and chemical exposure. **(B)** Schematic representation of an unconventional fragment removal of parts of the exon-2 and intron-2 from *hac*A mRNA, primers used for RT-PCR are indicated by gray boxes, the position of the excised fragment is shown by the color contoured box. **(C)** Prediction of the twin stem-loop structure of an unconventional splicing of *T. rubrum* and fungi from *hac*A mRNA carried out by mFOLD (Zuker, 2003) and the drawing of structures with VARNA (Darty et al., 2009). **(D)** Schematic representation of HacA induced isoform (*hac*A^*i*^) with 395 aa and uninduced isoform (*hac*A^*u*^) with 402 aa from *T. rubrum*. **(E)** Schematic representation of HacA induced (with 350 aa) and non-induced (with 441 aa) isoforms from *A. nidulans.* The introns in the *hacA* gene are represented by green boxes, and directly below both of the HacA isoforms are shown. The bZIP domain is shown in a pink box, the coil domain is shown in a blue rectangle, the mobiDB-lite domains are shown as dark green cylinders, a red triangle shows the DNA binding site, and the dimer interface residues are represented by a purple arrow. The transmembrane domain is shown in an orange lozenge and in a yellow lozenge is shown the non-cytoplasmatic domain, and the cytoplasmatic domain is shown as a gray box.

BlastX using *hac-1* from *Saccharomyces cerevisiae* as a query revealed TERG_05396 as a putative ortholog for this gene in *T. rubrum.* The predicted *hac-1/hacA* gene from *T. rubrum* contains 1281 nucleotides. The comparison among mRNA secondary structures of *hac-1/hacA* sequences from different fungal species evidenced a region of intron excision for prompt *hac-1/hacA* activation. Following sequencing of the *hacA* amplified products from *T. rubrum*, we showed that under ER stress, a fragment of 20 nt was removed from *hacA* mRNA. We also demonstrated that some antifungal compounds (griseofulvin and terbinafine) were responsible for *hacA* activation through the 20 nt fragment removal as well as the positive controls DTT and tunicamycin (Figure 1A). Curiously this fragment corresponds to portions of both exon-2 and intron-2, and even upon excision, part of intron-2 (47 nt) was retained (Figure 1B). Further, the consensus of this excision region and the surrounding regions generated a typical secondary structure of *hac-1/hacA*, in which the boundaries surrounding the intron/exon of excision made two little hairpins (Figure 1C). The removal of the fragment of 20 nucleotides changes the open reading frame arising a DNA binding site and dimer interface residues, which are considered crucial for UPR function in the homologs of HacA as already assessed in other fungi (Oh et al., 2010). The resultant protein is 395 amino acids long and contains a conserved bZIP domain, plus a coiled domain. The uninduced form is 402 amino acids long, and shows the bZIP domain and coiled domain, but neither the DNA binding site nor the dimer interface residues are presented (Figure 1D). Moreover, we ascertained a different pattern in the induced form of HacA from *T. rubrum* in comparison to the filamentous fungi such as *Aspergillus nidulans*, *A. fumigatus*, and *Neurospora crassa*, where the main changes between the induced and uninduced form were located on the 3′ portion of the protein (Figure 1E).

### Deletion of *hacA* Gene

Deletion of the *hacA* gene from *T. rubrum* was carried out by replacing the entire coding region with a hygromycin resistance cassette (*hph*) using a split-marker approach (Kuck and Hoff, 2010). The two overlapping fragments corresponded to 5′ UTR joined to *hph*, and 3′ UTR joined to *hph*, and were confirmed by PCR generating the expected products of 2611 and 3733 bp, respectively. Enzyme restriction using *Eco*RI (Thermo Scientific) also made the expected fragments of 452 and 2159 bp (5′UTR fragment), and 684, 1283, and 1766 bp (3′ UTR fragment). The PCR confirmed the loss of the *hacA* gene, and homologous integration was identified by genomic Southern blot analysis (Supplementary Figure S1).

### *hacA* Involvement in *T. rubrum* Susceptibility Toward Cytotoxic Compounds, and Thermotolerance

**FIGURE 2:**
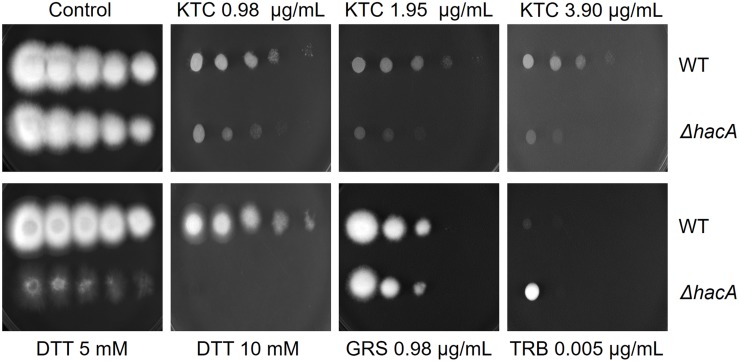
*hacA* plays a role in *T. rubrum* susceptibility toward antifungals and DTT. Susceptibility of *T. rubrum* strains to ketoconazole (KTC), in concentrations of 0.98, 1.95, and 3.90 μg/mL; DTT (5 and 10 mM); griseofulvin (GRS) in concentration of 0.98 μg/mL, and terbinafine (TRB), in a concentration of 0.005 μg/mL. Plates were inoculated, from left to right in each panel, with 10^6^, 10^5^, 10^4^, 10^3^, and 10^2^ conidia/mL and incubated at 28°C for 7 days.

**FIGURE 3:**
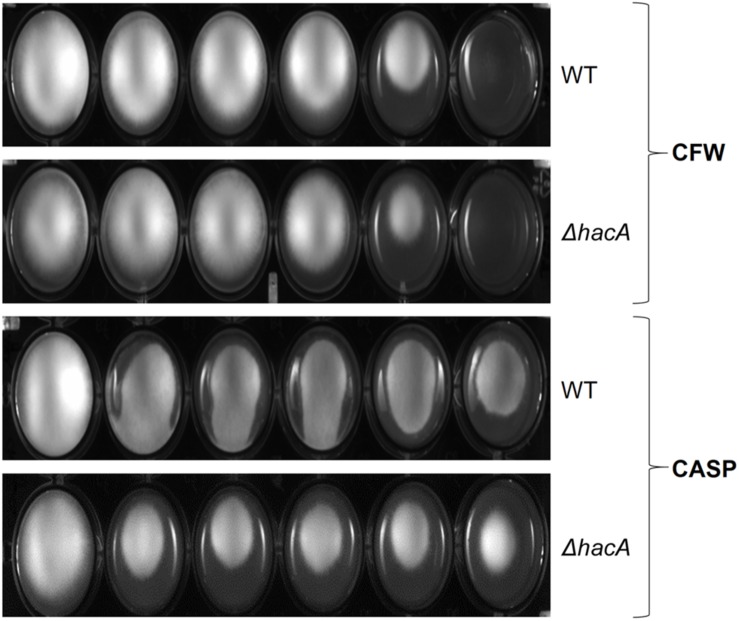
*hacA* affects *T. rubrum* susceptibility to cell wall inhibitor agents. Susceptibility of *T. rubrum* strains to CASP (0, 12.5, 25, 50, 100, and 200 μg/mL), and CFW (0, 5, 10, 20, 40, and 80 μg/mL). Plates were inoculated with approximately 1 × 10^5^ cells, and the drugs were added in corresponding concentrations from left to right, respectively, and incubated for 7 days at 28°C.

The growth of the Δ*hacA* strain was healthy under non-stressful conditions. However, we evidenced differences in growth rates under chemically-induced ER stress and antifungal exposure. The mutant strain showed an enhanced sensitivity to DTT (a reducing agent that leads to ER stress), compared to the wild-type strain (Figure 2) and a reduced growth was observed following KTC exposure, whereas under TRB exposure the mutant strain presented a slight increase in growth (Figure 2). In regard to compounds that act on the cell wall, slight differences in the growth rate were noticed for the Δ*hacA* strain during CFW exposure, primarily in the higher concentrations of 40 and 80 μg/mL, while CASP promoted a slight reduction in colony growth at all tested levels (Figure 3).

**FIGURE 4:**
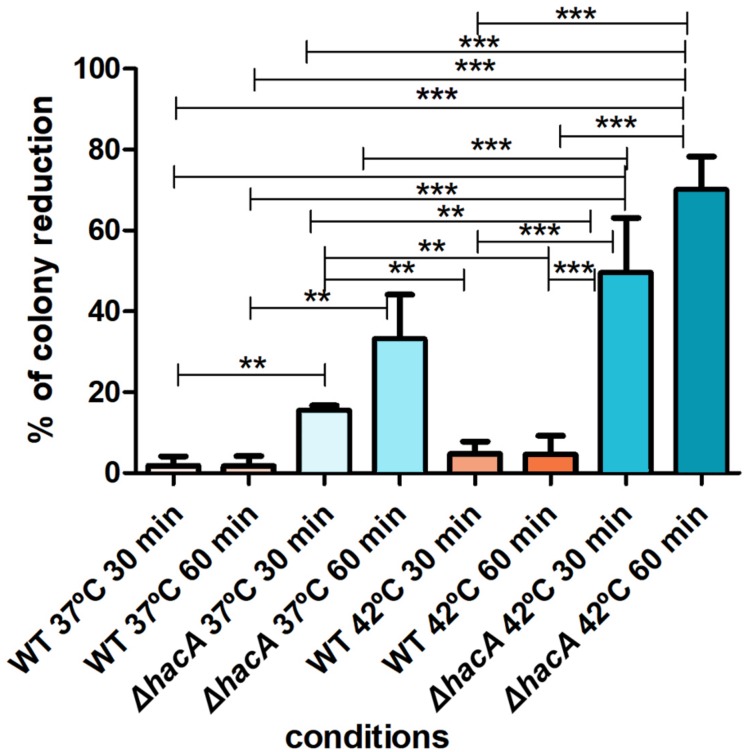
*hac*A affects thermotolerant growth. Equal concentrations of conidia from each strain were exposed to different temperatures (37 and 42°C) for 30 and 60 min. The control consisted of conidia from both strains without thermal exposition. Thereafter, conidia were inoculated in Sabouraud medium. The percentage reduction for the number of colonies is depicted in this graph. Significantly different values are shown by asterisks, and were determined using ANOVA followed by Tukey’s *ad hoc* test (**P < 0.01; ***P < 0.001).

The activation of the UPR enhances the protein folding and contributes to protein secretion, thus favoring the adaptation of pathogenic fungi for intracellular survival. Notably, fast-paced proliferation at mammalian body temperature is an important virulence attribute that favors adaptation. HacA seems to play a pivotal role in orchestrating adaptive responses to thermal stress (Richie et al., 2011; Boppidi et al., 2018). Since higher temperatures induce protein conformational changes and alter protein secretion (Mori et al., 2000), we assessed the ability of conidia of both strains to withstand different temperatures (37 and 42°C). Indeed, the mutant was found to be thermosensitive to a higher extent. Analysis of the number of colonies showed a decrease when the mutant was cultivated at 37°C (approximately 15 and 33% for 30 and 60 min of exposure, respectively) and a marked reduction was observed at 42°C (approximately 50 and 70% for 30 and 60 min, respectively). Meanwhile, the wild type strain showed less than a 5% reduction in the number of the colonies for both temperatures assessed (Figure 4). These data suggest that *hacA* was of paramount importance for *T. rubrum* growth at high temperatures.

### *hacA* Association With Nutritional Versatility

**FIGURE 5:**
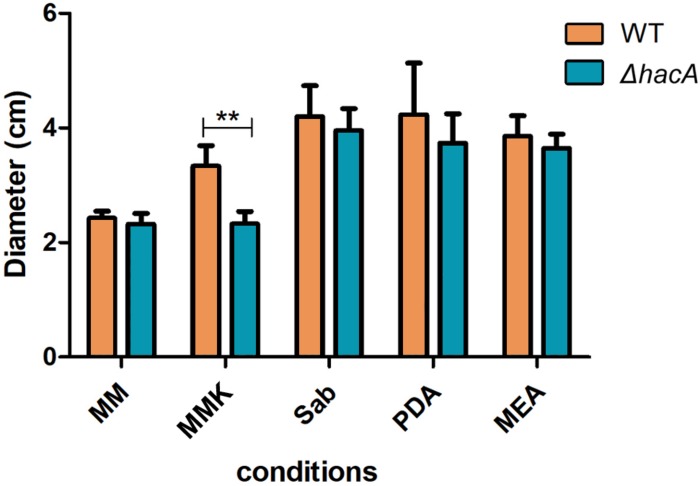
*hacA* supports *T. rubrum* growth on keratin sources. An equal plug from each indicated strain was spotted onto plates with a different culture medium. The growth rate (diameter in cm) was calculated for each culture, and each strain after 9 days at 28°C. MM, minimal medium; MMK, minimal medium contained keratin; Sab, Sabouraud; PDA, Potato dextrose agar; MEA, agar malt extract. Statistical significance was determined using Two-Way RM ANOVA followed by Bonferroni’s post-test (**P < 0.01).

During host-pathogen interaction, *T. rubrum* encounters a stressing environment, and to stablish the infection the fungus employs metabolic reprogramming to make productive use of the keratin from the host as a nutrient source (Martinez-Rossi et al., 2016b; Mercer and Stewart, 2019; Monod and Mehul, 2019). In order to determine the importance of *hacA* to nutritional versatility different media were tested like standard media for fungal growth such as Sabouraud, MEA, and PDA (representing rich substrates of pre-digested proteins), or Cove’s medium (Cove, 1966) (as a restrictive substrates source), or Cove’s medium supplemented with keratin (representing a complex protein source that mimics the dermatophyte infection). Although both strains showed equivalent growth rates on less complex substrates, consistent differences were observed for growth on the keratin substrate. The growth of mutant strain on keratin was impaired (Figure 5). Notably, differences in pigmentation were found between the wild type and the mutant colonies during growth on Sabouraud, MEA, and PDA (Supplementary Figure S2). These results hinted that a compromise in pathways related to keratin utilization exists, and also identified changes in secondary metabolite production.

### *hacA* Regulates Membrane Homeostasis and Cell Wall Composition

**FIGURE 6:**
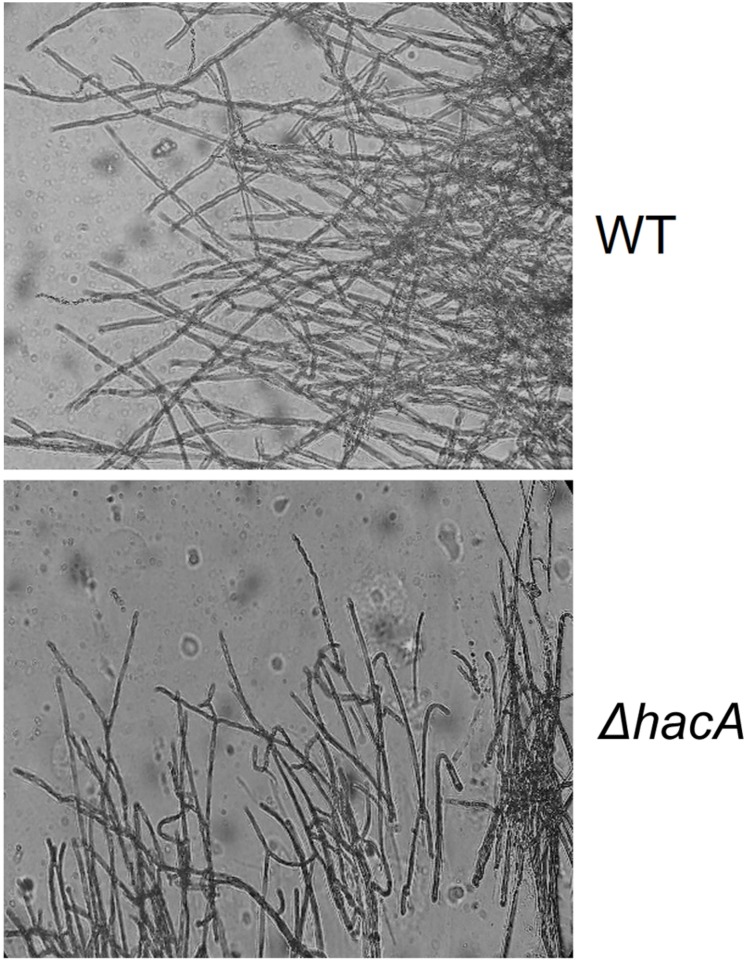
*hacA* supports hyphae directionality in *T. rubrum*. A microculture from both strains after 6 days at 28°C under microscopy light at magnification ×200.

**FIGURE 7:**
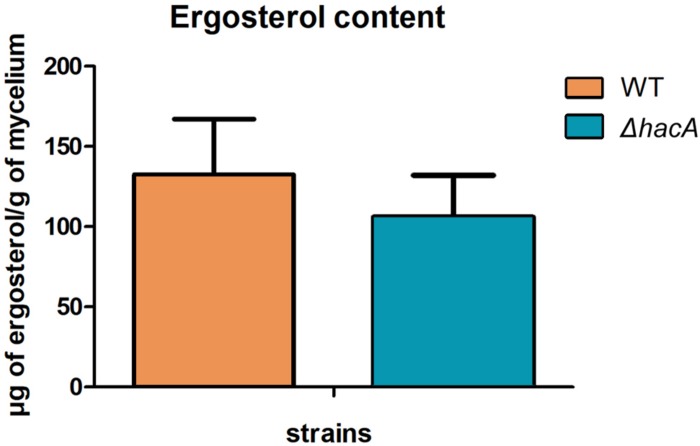
*hacA* contributes to ergosterol biosynthesis in *T. rubrum*. Ergosterol content in wild type and mutant strains assessed per gram of mycelium dry weight.

The cell wall is the primary interface between fungus and host. As described previously in this work, Δ*hacA* showed differences in the growth rate in comparison to the wild type after exposure to the cell wall stressing agents. Further, under microscopic observation of the *T. rubrum* strains, a marked curling of the hyphae was observed for the mutant strain which suggested a defect in the hyphae directionality (Figure 6). Additionally, a marked impairment in protoplast regeneration was assessed for the Δ*hacA* mutant (data not shown). As ergosterol is the principal sterol within the cellular membrane with roles in fluidity, membrane protein assembly (Lv et al., 2016), and also in cell wall composition, we assessed the ergosterol content in both strains. The results showed a slight reduction in this content for the mutant strain (Figure 7).

### *hacA* Participates in Fungus-Host Interaction

**FIGURE 8:**
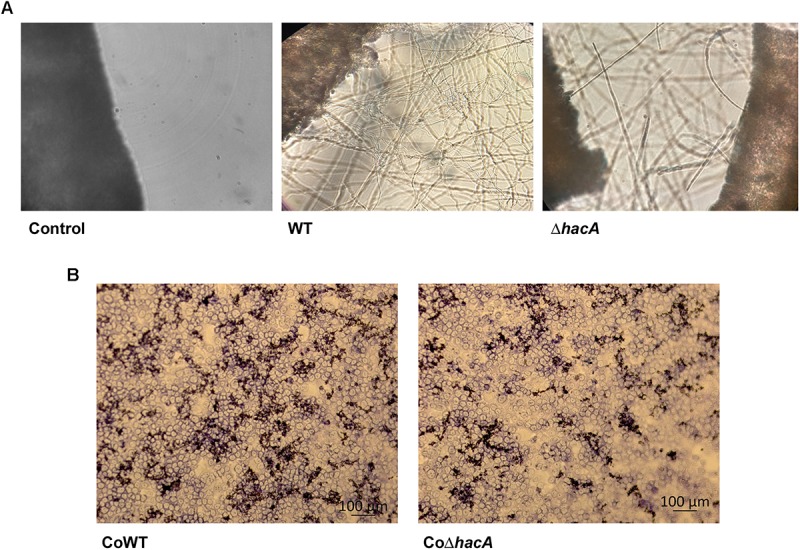
*hacA* contributes to host-fungi interaction. Effect of the *hacA* gene deletion on the growth of *T. rubrum* on human molecules. **(A)** Conidia from wild type and mutant strain were incubated on human nails for 72 h at 28°C. Fungal growth was observed by light microscopy. The black objects seen on the left side are related to nail fragments. **(B)** Coculture of *T. rubrum* conidia from both strains with keratinocyte cell type HaCaT for 24 h at 37°C.

**FIGURE 9:**
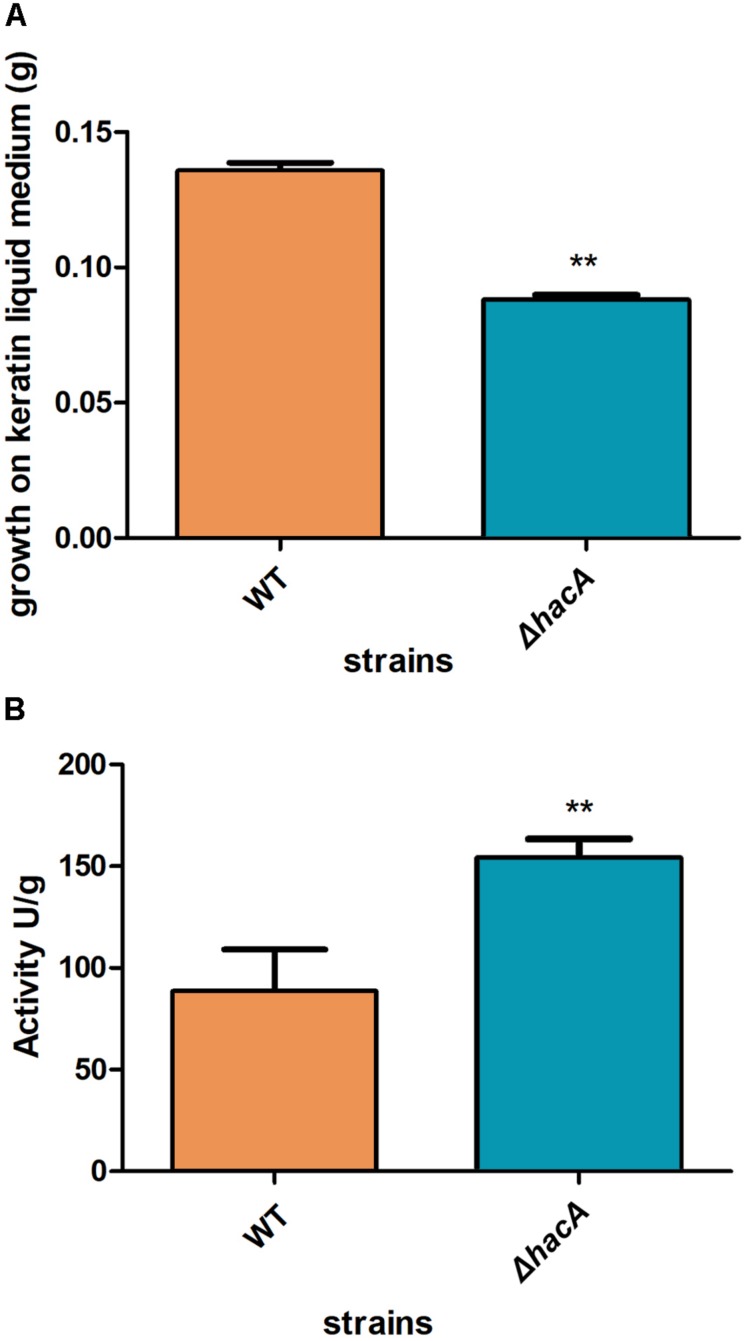
*hacA* supports keratinolytic feature of *T. rubrum*. **(A)** Dry weight mycelium from wild type and mutant strains expressed per gram of dry weight mycelium. **(B)** Keratinolytic activity from both strains was determined as mycelium-specific activities in units per gram of dry weight mycelium. Statistical significance determined using Unpaired *t*-test (**P < 0.01).

We performed an interaction between conidia from both strains, wild type, and Δ*hacA*, with human nail fragments as well as in co-cultures with keratinocytes to assess virulence traits. These results showed decreased ability of the *hacA* mutant to grow on human nail fragments. Further, the directionality of hyphae seemed to be impaired, and the curling hyphae were evidenced (Figure 8A and Supplementary Figure S3). Moreover, the co-culture assay demonstrated a decrease in hyphal development for the Δ*hacA* mutant in comparison to the wild type (Figure 8B), which correlated with reduction of growth on the keratin media (Figure 9A). Otherwise, the activity of the secreted keratinolytic proteases was increased in the *hacA* mutant in comparison to the wild-type when both strains were cultivated on keratin powder (150 and 90 Units/g dry weight of mycelium, respectively (Figure 9B). This uncorrelated data might be due to a metabolic rearrangement as an attempt to use this complex protein substrate. Previous work reported that *T. rubrum* under undecanoic acid exposure presented a decrease in keratin growth and an increase in keratinolytic activity, which might be a consequence of changes in enzymes by post-translation modifications (Mendes et al., 2018).

**FIGURE 10:**
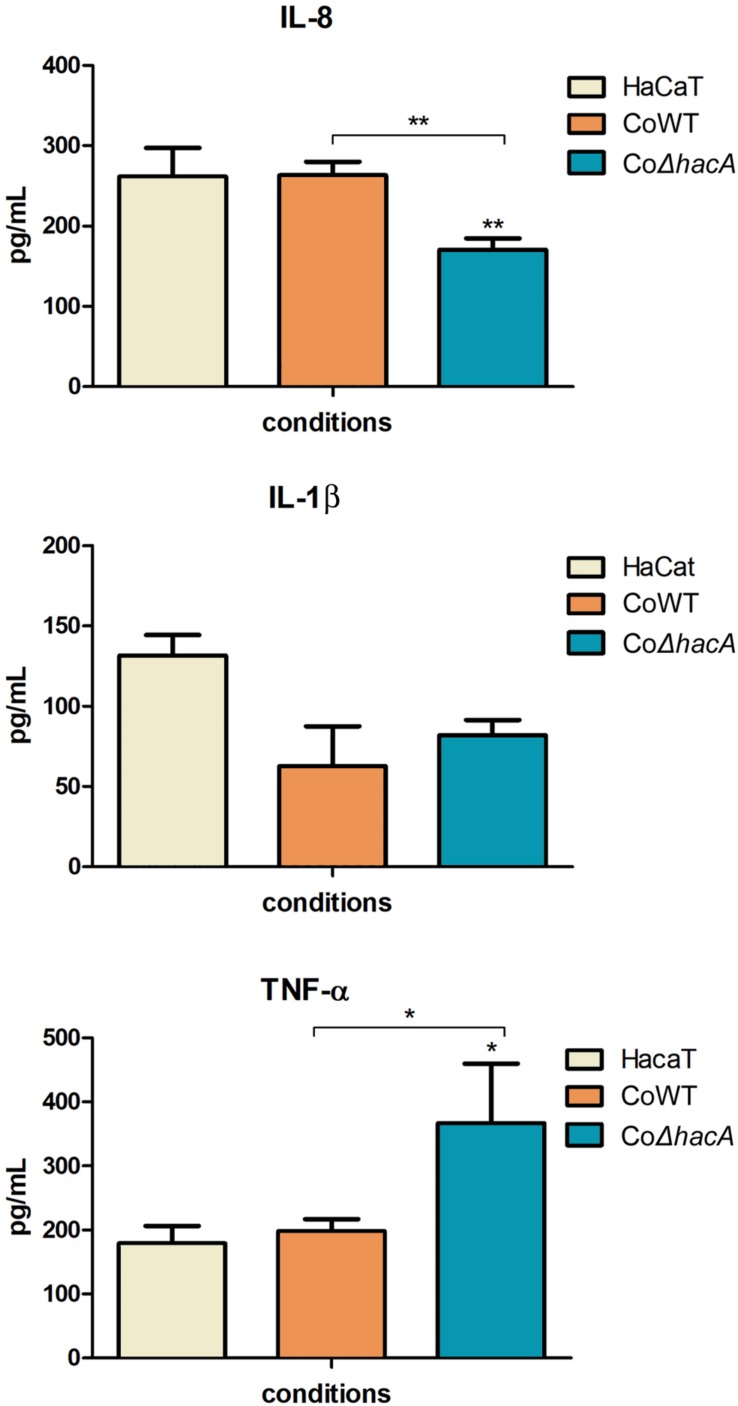
*hacA* participates in immune modulation in keratinocyte cells. Levels of pro-inflammatory cytokines output by HaCaT after coculture with conidia from the wild type and mutant strain for 24 h. Comparison of IL-8, IL-1β, and TNF-α secretion by HaCaT. Significantly different values are shown by asterisks, and were determined using ANOVA followed by Tukey’s *ad hoc* test (*P < 0.05; **P < 0.01).

During fungus-host interaction, the fungal cell wall was the first line of contact with the host. Thus, the composition of the cell wall offers a wide array of molecules that act as patterns for recognition by host immune system and profoundly impact the relationship between host and pathogen. The receptors activated by these molecules are called pattern recognition receptors (PRRs), and their activation triggers intracellular signaling that leads to the production of pro-inflammatory mediators, like cytokines. In this work, we assessed the levels of IL-1β, IL-8, and TNFα after 24 h of co-culture of the human keratinocyte line (HaCaT) with conidia from both strains, the *hacA* mutant promoted differences in cytokine production, were related to an increase in TNFα and a decrease in IL-8; however, no significant differences were assessed for IL-1β (Figure 10). This result highlights the differences in cell wall patterns for the mutant strain.

### *hacA* Regulates the Expression of Genes Belonging to Different Biological Processes

**FIGURE 11:**
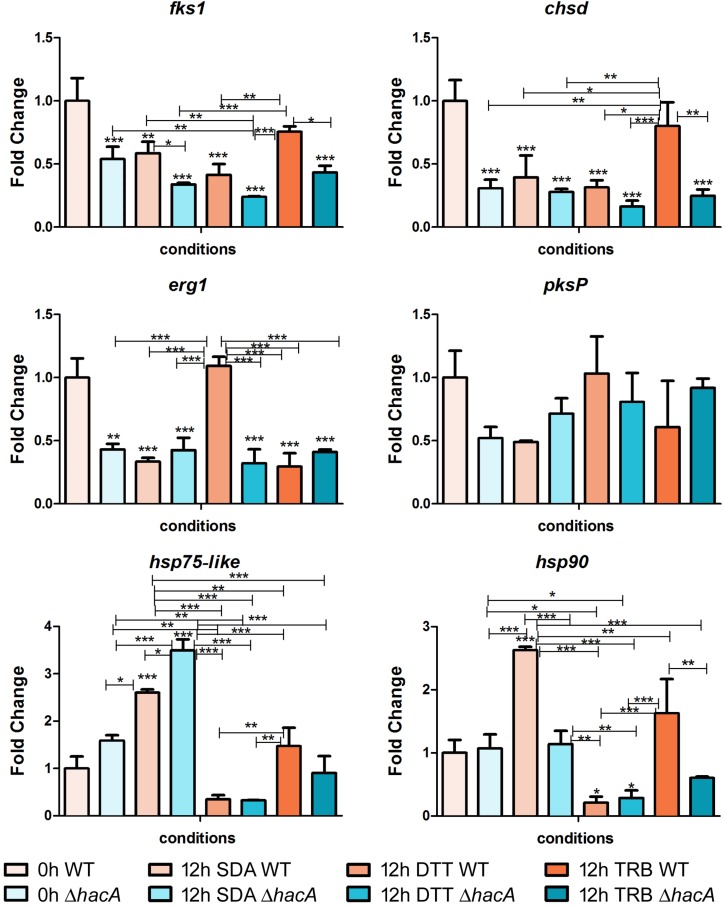
*hacA* regulates genes belonging to the different metabolic processes. Transcriptional levels of encoding genes of *chsD*, *fks1*, *hsp75-like*, *hsp90, erg1*, and *pkP* evaluated by qPCR for 12 h of both *T. rubrum* strains (wild type and Δ*hacA*) growth on Sabouraud (SDA), or SDA with DTT (10 mM), or Terbinafine (TRB in 0.014 μg/mL) compared to 0 h (control). Significantly different values are shown by asterisks, and were determined using ANOVA followed by Tukey’s *ad hoc* test (*P < 0.05; **P < 0.01; ***P < 0.001). Asterisks close to the bars are related to the comparison with the calibrator (0 h WT).

**FIGURE 12:**
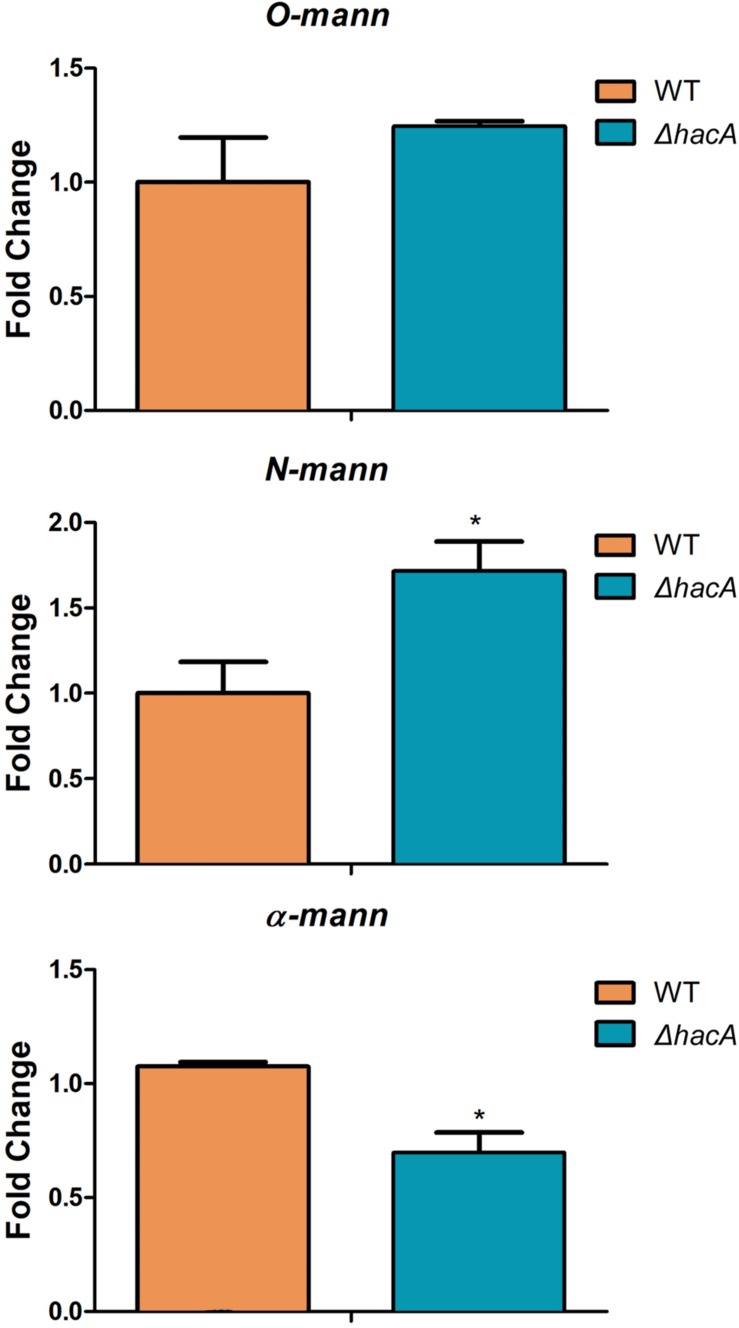
*hacA* regulates genes coding for mannosyltransferase in *T. rubrum*. Transcriptional levels of *N-mann*, *O-mann*, and α*-mann* from *T. rubrum* conidia cocultured with human keratinocyte cell line HaCaT for 24 h. Statistical significance was determined using Unpaired *t*-test (*P < 0.05).

To assess genes that are directly or indirectly regulated by HacA, we evaluated the modulation of genes related to cell wall synthesis, ergosterol biosynthesis, pigmentation, heat shock proteins, and the genes coding for mannosyltransferase enzymes (Figures 11, 12).

Among cell wall encoding enzymes, we assessed the gene modulation of *fks1* and *chsD*, and we observed marked differences in control (0 h) between the wild type and mutant, with a significant decrease in transcript levels for the mutant strain. Further, over time (additional 12 h of fungal growth), the mutant strain exhibited reduced transcription levels of *fks1*, compared to the wild type, whereas no differences in transcript levels were shown for *chsD* among the strains at the same time point. We also demonstrated that in response to terbinafine exposure, an increase in *chsD* transcriptional levels was assessed for the wild type, probably as an adaptive response leading to cell wall remodeling. Several lines of evidence point to the UPR impact on the fungal wall and cell membrane, suggesting potential connections between cell wall integrity (CWI) pathway and UPR (Malavazi et al., 2014). Moreover, previous work showed the relevance of *chsB* and *chsD* genes for the cell to cope with cell wall stress caused by *pkcA* deletion in *A. fumigates* (Rocha et al., 2015).

The *erg1* transcript levels showed a marked decrease for the mutant in comparison to the wild type control (0 h). Chemical treatment with DTT promoted an up-regulation of *erg1* from the wild type strain, while no significant differences were observed for the mutant. DTT induces an ER stress and as this organelle is linked to lipid synthesis; thus, could be possible that induction on the *erg1* transcript levels was an attempt to cope with the ER perturbation. Conversely, no differences were found in the transcript levels of *erg1* were verified for the TRB treatment at this time point.

The analyses of gene modulation for genes coding for Hsp90 and Hsp75-like exhibited different profiles among the studied strains. After 12 h of fungal growth, the wild type showed a prominent induction at the *hsp90* levels, whereas at this same time point the mutant strain prompted a rise in the *hsp75-like* levels. These results might reflect a compensatory modulation in the Δ*hacA* strain. However, when exposed to terbinafine, only the wild type maintained elevated transcript levels for both Hsp encoding genes. It is already known that Hsp is involved in the sensing and adaptation of pathogens to thermal conditions as well as diverse conditions. In this context, previous reports have suggested Hsp90 as a potential therapeutic target as it is an abundant Hsp (corresponding to approximately 2% of the protein repertoire) which contributes to cell wall integrity, germination, pigmentation of conidia, drug resistance, and virulence (Jacob et al., 2015; Martinez-Rossi et al., 2016a, 2018). Remarkably, impairment of regulation in the mutant strain might be correlated with a compromise in cell wall composition, thermal tolerance, drug resistance, and changes in colony pigmentation previously described in this work.

In an attempt to analyze differences in pigmentation, the *pksP* gene modulation was assessed, and a slight difference in regulation was verified for the control (0 h) between strains. Notwithstanding, the DHN-melanin pathway consists of other genes that could be directly or indirectly regulated by HacA like *pksP*, *arp1*, and *arp2*. Another study has also reported that differences in the melanin of *A. fumigatus* within the conidial cell wall was found to be related to both stress tolerance and virulence (Hagiwara et al., 2017).

We also assessed the modulation of *alpha-mannosyltranferase*, *N*-mannosyltransferase, and *O*-mannosyltransferase genes because these results would be linked to differences in the immune responses triggered by each strain as was described previously in this article. We evaluated their modulation using RNA extracted from co-cultures. These results revealed significant changes in expression levels of the *N*-mannosyltranferase and the alpha-mannosyltranferase encoded genes within the Δ*hacA* mutant (Figure 12).

**FIGURE 13:**
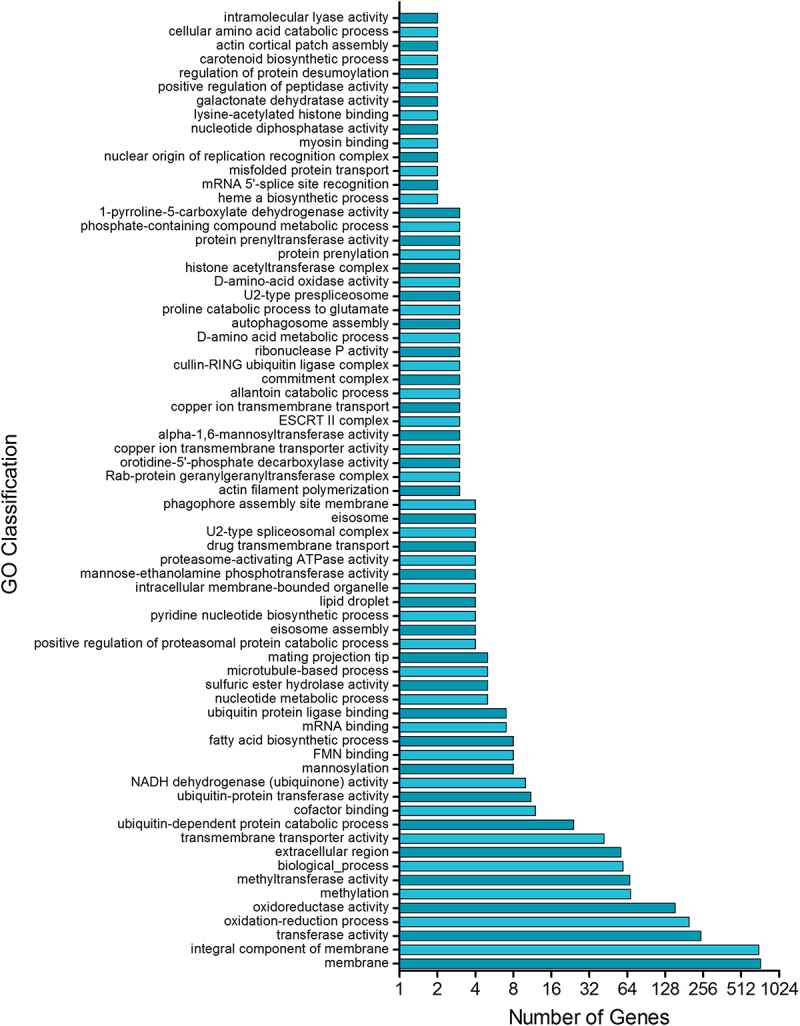
Putative HacA target genes in *T. rubrum* genome. Functional enrichment of genes in *T. rubrum* genome with a recognition site for UPRE-1 or UPRE-2 or UPR-3 motif consensus.

Together, these results are consistent with the analysis of the promoter region of the *T. rubrum* genes which matched the consensus motif of UPRE-1, UPRE-2, or UPRE-3, which are known as unfolded protein response elements. Our analysis showed that approximately 25% of *T. rubrum* genome might be potentially regulated by HacA (Supplementary Table S2). Among them are the mannosyltransferase enzymes, Hsps, fatty acid biosynthetic enzymes, cell wall enzymes, and proteases (Supplementary Table S2). The functional enrichment of these genes showed some main categories, such as fatty acid biosynthetic processes, methyltransferase activity, membrane composition and transport, oxidoreductase, and pyrimidine nucleotide biosynthetic processes (Figure 13).

## Discussion

Endoplasmic reticulum (ER) is the gateway for the secretory pathway and is the center for post-translational modification, accurate folding, and assembly of up to 30% of the cellular proteome (Schwarz and Blower, 2016). Fungi are organisms specialized for secretion and the utilization of this mechanism is entwined to the ability to sense environmental stress, thus leading to adequate responses (Krishnan and Askew, 2014). As homeostasis in the ER secretory capacity is followed by UPR activation, and current research has reported that the UPR genes are important vulnerability points to be exploited in fungal therapy, we decided to address the scope of *hacA* in the dermatophyte *T. rubrum*.

A non-canonical splicing of *hacA* mRNA mediates the activated form of HacA through IreA, The RNase domain of IreA is responsible for catalyzing the intron removal from *hacA* in a spliceosome independent manner, which in turn leads to a shift in the open reading frame, and the arisen of bZIP domain in activated HacA (Mori et al., 2000). Here, we demonstrated that the fragment which is removed from *T. rubrum hacA* mRNA is part of exon-2 and part of intron-2, and removal of this fragment prompt a shift in the open reading frame and the arisen in the DNA binding site, and then this activated form would be ready to go to the nucleus to bind with the target genes.

Noteworthy, peculiarities in this pathway were described herein, and they were related to differences in the *hacA* gene sequence arrangement since within this dermatophyte this gene is composed of 2 introns whereas in *A. nidulans*, *A. fumigatus*, and *N. crassa* present only one intron. In addition, features of the induced form of HacA from *T. rubrum* are also different in comparison to these other proteins from *Aspergillus* spp. and *N. crassa*. In these species, the changes in induced form occur in the 3′ portion of the protein, with no differences observed in the DNA binding site or in the dimer interface residues (Figures 1D,E). Remarkably, particularities in this well-characterized process were previously described (Montenegro-Montero et al., 2015), and they strengthen the importance of investigating different systems to address its involvement in fungal lifestyle and niche association preferences.

In filamentous fungi such as *A. fumigatus*, approximately 10% of the whole genome may be potentially regulated by IreA-HacA, whereas in *Saccharomyces cerevisiae* it is estimated that HacA might have regulated 5% through the UPRE-1 recognition sites (Fordyce et al., 2012; Krishnan and Askew, 2014). In this sense, another work showed that although all known bZIP transcription factors adopted, in general, one binding mode, HacA might present more binding sites, which correlated with the vast target repertoire (Fordyce et al., 2012). Indeed, it is assumed that normal growth conditions also require UPR activation. Bioinformatic analyses carried out here included searches for UPREs within the target gene promoters of *T. rubrum* identified a total of 2,178 target genes. These genes belong to diverse categories such as genes coding for the cell wall components, enzymes involved in fatty acid synthesis, Hsps, proteases, mannosyltransferase enzymes, and genes related to transcription and translation processes (Supplementary Table S2).

Thereafter, we hypothesized the requirement of UPR is dependent on HacA under different stressors, and we verified its traits of thermotolerance and in drug susceptibility. The results exhibited a marked sensitivity for the mutant strain during fungal growth at 37°C, and the most prominent effect occurred at 42°C. Although dermatophytes typically infect the skin, hair, and nails, there are some case reports of invasion into the dermis, subcutaneous tissue, or internal tissues (Marconi et al., 2010; Dai et al., 2019), which require adaptation to higher temperatures. Further, differences in the pigmentation of the colonies may be correlated to increased thermal sensitivity, since there is a link between melanin biosynthesis, thermal environment, and stress tolerance as was demonstrated for conidia from *A. fumigatus* (Hagiwara et al., 2017). In addition, this thermal sensitivity could be related to a reduction in ergosterol content, but we did not find compelling evidence for the *T. rubrum* mutant strain. Nonetheless, even a small reduction in ergosterol may destabilize the cellular membrane, and high temperatures can affect membrane fluidity and permeability (Simonin et al., 2007). Herein, we assumed that both correlated conditions contribute to inability of conidia survival under thermal stress. Also, changes in ergosterol levels and impairment in the cellular membrane might be entwined to sensibilization of the mutant strain toward ketoconazole. In *A. fumigatus* mutants (Δ*ireA* and Δ*hacA)*, an increase in azole susceptibility was also assessed (Feng et al., 2011).

In regard to cell wall impairment, there was evidence that this effect correlated with defects in the cellular membrane composition, and potentially a link between impairment of membrane composition and a decrease in the protoplast regeneration ability (Bitencourt et al., 2013). Indeed, the deletion of *hacA* in *T. rubrum* affected the hyphae directionality, compromised protoplast regeneration, and enhanced its susceptibility to cell wall inhibitors. The involvement of UPR in hyphae formation and cell wall integrity pathways were previously described (Krysan, 2009; Rocha et al., 2015). Beyond this, differences in cell wall patterns in the mutants for UPR were intimately linked to differences in virulence and host-pathogen interactions in other fungi (Cheon et al., 2011; Feng et al., 2011; Richie et al., 2011). These traits might be related to the difficulty of these mutants to grow at 37°C, as well as due to the involvement of UPR in host attachment. Indeed, our data showed a decrease in Δ*hacA* growth at the HaCaT cell line and human nail fragments. Further, we also demonstrated differences in the production of pro-inflammatory cytokines after infection with the wild type and Δ*hacA* strains. In summary, our data suggest a different pattern in cell wall organization, which might contribute to different pro-inflammatory cytokine release profiles. Previous works have shown that post-translation modification conducted in molecules from the cell wall had prompted recognition by different PRRs, and finally triggered differences in the immune response pattern via the keratinocyte surface (Figueiredo et al., 2011; Snarr et al., 2017). Furthermore, differences in cytokine release by keratinocytes are reportedly associated with the acute or chronic lesions caused by zoophilic or anthropophilic dermatophytes, respectively (Hau et al., 2015). While zoophilic species lead to a broad spectrum of cytokine release, including members from Th1, Th2, and Th17 response, anthropophilic species only induced the expression of IL-6, IL-8, IL-1β, and eotaxin-2 (Shiraki et al., 2006).

Our findings demonstrated that the mutant strain from *T. rubrum* showed decreased growth on keratin as the sole substrate. Previous studies have described the role of the *UPR* genes in supporting the growth of *A. fumigatus* in the lungs and that of *A. niger* in maltose as the growth substrate (Jorgensen et al., 2009; Feng et al., 2011). Involvement of the integrated pathways ERAD and UPR in the utilization of complex substrates was confirmed in the Δ*hacA*/derA mutant from *A. fumigatus* grown on lung and skim milk (Richie et al., 2011). Other studies have reported that the growth of filamentous fungi on complex substrates required high activity from the secretory pathway for this condition needed to elicit higher amounts of extracellular enzymes, which was accompanied by activation of UPR (Feng et al., 2011; Richie et al., 2011). Curiously, enhanced keratinolytic activity was exhibited for the Δ*hacA* mutant strain. We hypothesized that this strategy was due to a metabolic rearranging as an attempt to compensate for difficulties in the utilization of this complex protein substrate.

## Conclusion

Our data unveil for the first time the involvement of HacA in dermatophytes physiology, response to stress, and host-pathogen interaction. Notwithstanding, a deeper understanding of the complex cross-talking of HacA with different metabolic pathways is paramount in importance to widen the knowledge about this potent transcriptional regulation. Regardless of how this will be addressed in the future, this work underscores the critical roles of HacA in *T. rubrum* virulence and adaptive responses. Together, these results provide valuable information about the efficacy of the use of HacA as a potential molecular target for novel antifungal therapy.

## Data Availability Statement

All datasets generated for this study are included in the article/Supplementary Material.

## ETHICS STATEMENT

The Committee of Ethics in Human Research of the Ribeirão Preto Medical School at the University of São Paulo approved all experiments involving the use of human nail fragments provided by healthy adults (Protocol No. 8330/2009). The patients/participants provided their written informed consent to participate in this study.

## Author Contributions

NM-R, TB, and AR conceived the study and wrote the manuscript. TB performed the experimental design and laboratory experiments. AF and EL contributed with deletion cassette construction. PS performed the bioinformatics analysis. NP assisted in the immunological assays. VO assisted in the microbiological assays.

## Conflict of Interest

The authors declare that the research was conducted in the absence of any commercial or financial relationships that could be construed as a potential conflict of interest.
