# Supplementary material for: HacA Governs Virulence Traits and Adaptive Stress Responses in Trichophyton rubrum
Source: Front Microbiol. 2020 Feb 20;11:193. doi: 10.3389/fmicb.2020.00193 (PMC7044415; doi:10.3389/fmicb.2020.00193)
Supplement: Supplementary file 1 [file Data_Sheet_1.PDF]

Supplementary Material

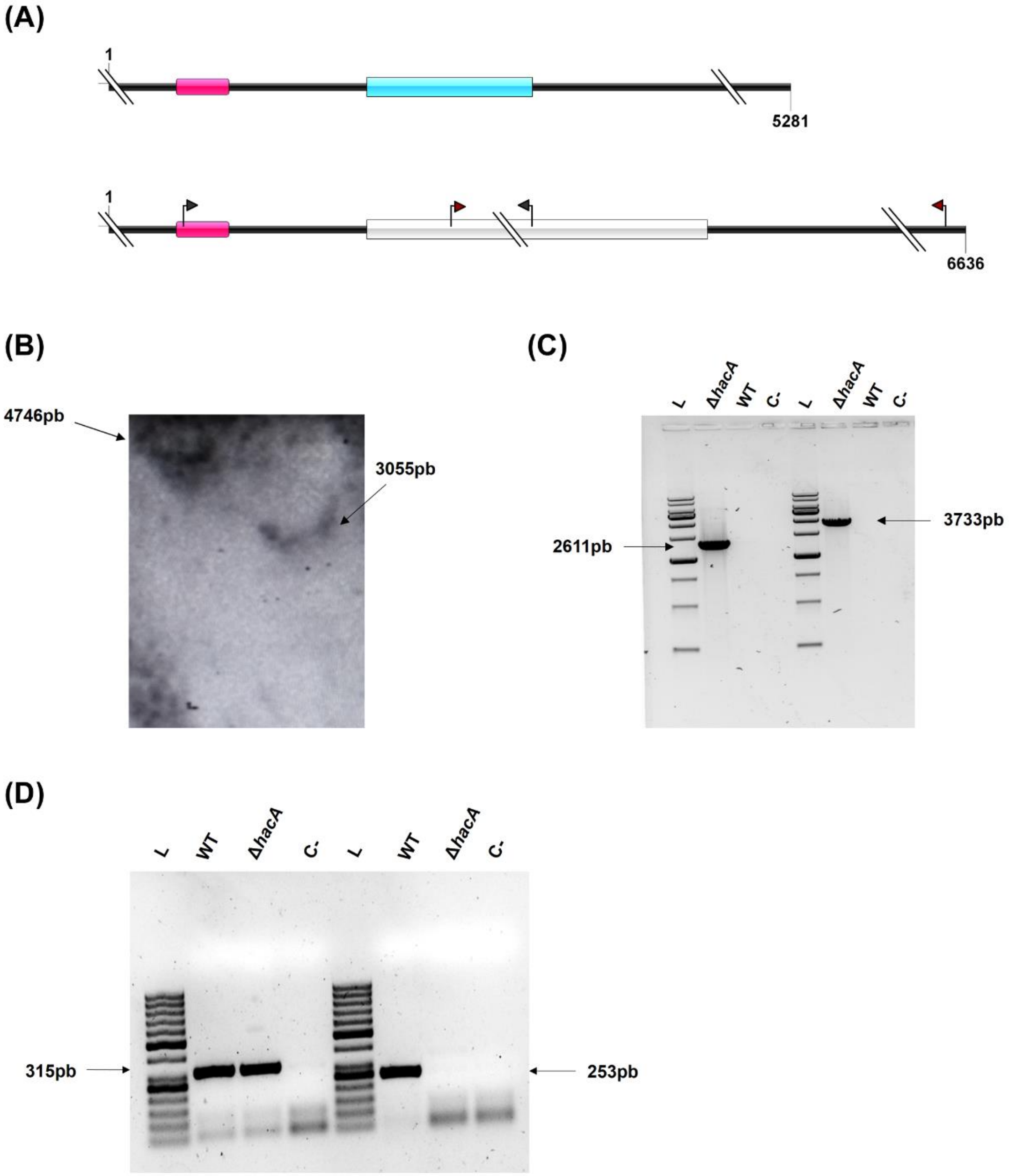

Supplementary Figure S1. Schematic representation of the Southern blot assay. (**A**) Overview of *hacA* gene: coding sequence indicated by a light blue rectangle, and 5′ UTR and 3′ UTR regions depicted with black rectangles. The deletion cassette is represented by the removal of the coding sequence and the insertion of a hygromycin resistance gene (light gray rectangle). Restriction sites for BtgZI are represented by cut lines, and the *hacA* fragment which was used as probe is indicated by a pink box. Arrows are used to represent primers used for PCR reactions carried out to gene deletion confirmation. (**B**) Southern blot of the digested DNAs with BTgZI was probed with a *hacA* fragment. The expected fragments are depicted in the figure as 4746 pb for WT and 3055 pb for mutant strain. (**C**) Amplification with primers flanking the region of 5’UTR*hacA* and *hph* or 3’UTR*hacA* and *hph* from Δ*hacA* strain. (**D**) Amplification of a 253 bp internal region of the *hacA* gene from wild type strain. Amplification with primers for ß-tubulin (315 pb) was used as a control.

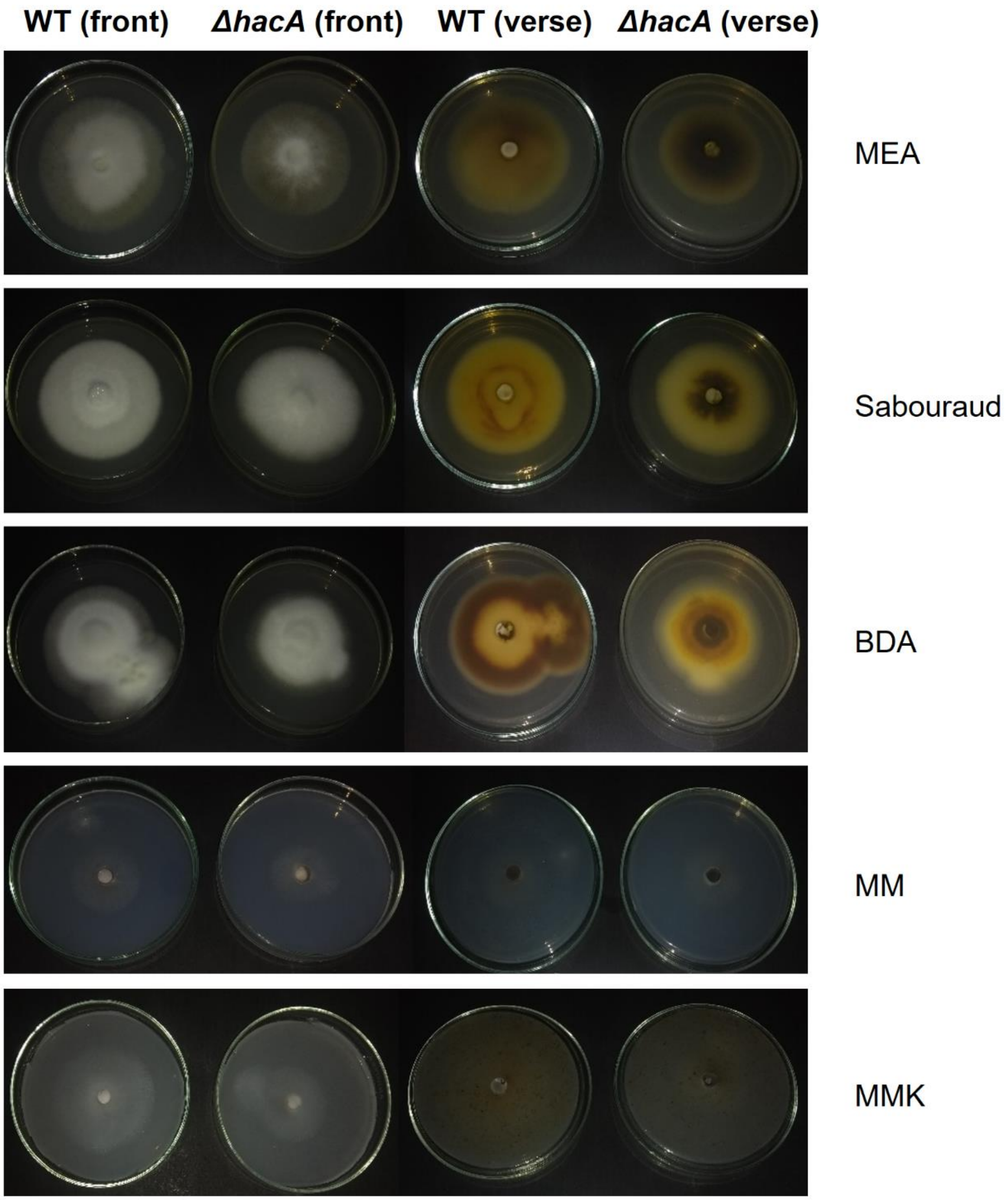

Supplementary Figure S2. *hacA* is involved in *T. rubrum* pigmentation during growth in different cultures.

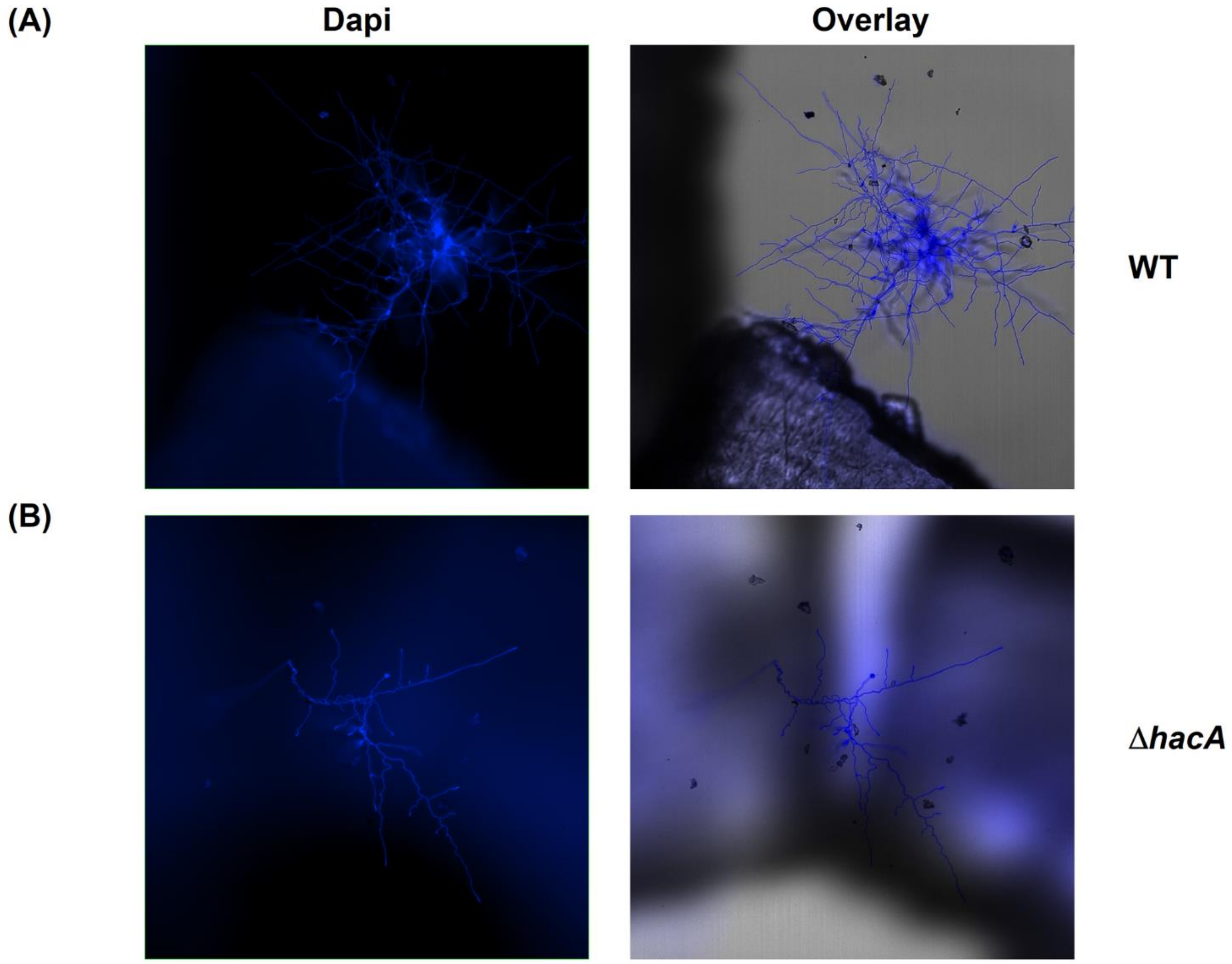

Supplementary Figure S3. Analysis of *hacA* gene deletion on the growth of *T. rubrum* on human nail fragments after staining with CFW 2 µg/ml for 5 min. Conidia (approximately 10^4) from (**A**) wild type and (**B**) mutant strain were incubated on human nails for 72 h at 28 ºC. Stained fungal cells was visualized under DAPI channel using ImageXpress Micro XLS system at magnification ×100.

Supplementary Table S1. Set of primers used for the *hacA* deletion cassette.

ID	Sequence 5’-3’	pb
hacA 5’UTR	F: GACGACTTGGGTCTGCATAT<br>R: ACTGGCCGTCGTTTTACGGTGTGGCTGTCTTCTGTCAAA	2012
hacA 3’UTR	F: GTCATAGCTGTTTCCTGGAGTGCAATGCTCGGATC<br>R: ATCACAAGCGGGTAACATCCAC	1994
hph marker	F: GTAAAACGACGGCCAGT<br>R: CAGGAAACAGCTATGAC	2636
5’ hacA split marker	F: CGGCCTGGAACTGGTATTATT<br>*hph* R: GATGTTGGCGACCTCGTATT	2611
3’hacA split marker	*hph* F: CTGCCTGAAACCGAACTGC<br>R: GGGAACACATCCACAGGTATTG	3733

Supplementary Table S2. *In silico* prediction of DNA-binding sites for the HacA consensus (UPRE-1 CAGNGTG, UPRE-2 TACGTG ,UPRE-3 AGGACAAC).

ID	Gene Product Name	Consensus	Position	Type
TERG_00004	alanine aminotransferase (*T. tonsurans*)	CAGTGTG	710	UPRE-1
TERG_00014	hypothetical protein	CACCCTG (rc)	190	UPRE-1
TERG_00015	stromal membrane-associated protein (*T. equinum*)	CACTCTG (rc)	209	UPRE-1
TERG_00026	flavin-binding monooxygenase, putative (*T. verrucosum*)	CAGGGTG	330	UPRE-1
TERG_00033	chromosomal organization and DNA repair protein Mms21, putative (*T. verrucosum*)	CACTCTG (rc)	992	UPRE-1
TERG_00034	mRNA cap guanine-N7 methyltransferase	CACTCTG (rc)	685	UPRE-1
TERG_00041	hypothetical protein	CAGGGTG	267	UPRE-1
TERG_00056	IBR domain-containing protein (*M. gypseum*)	CACCCTG (rc)	170	UPRE-1
TERG_00059	hypothetical protein	CACCCTG (rc)	150	UPRE-1
TERG_00060	GPI anchored cell wall protein, putative (*A. benhamiae*)	CAGGGTG	647	UPRE-1
TERG_00061	GDP-mannose pyrophosphorylase A (*T. equinum*)	CACCCTG (rc)	392	UPRE-1
TERG_00062	dynamin GTPase, putative (*T. verrucosum*)	CAGGGTG	623	UPRE-1
TERG_00078	nicotinate (nicotinamide) nucleotide adenylyltransferase	CACGCTG (rc)	820	UPRE-1
TERG_00084	chromosome segregation protein (Pcs1), putative (*T. verrucosum*)	CACGCTG (rc)	953	UPRE-1
TERG_00092	ubiquinone biosynthesis methyltransferase coq5 (*T. equinum*)	CACGCTG (rc)	365	UPRE-1
TERG_00099	cytochrome P450 family protein (*T. equinum*)	CAGTGTG	951	UPRE-1
TERG_00100	flavin-containing monooxygenase (*T. equinum*)	CACACTG (rc)	557	UPRE-1
TERG_00103	hypothetical protein	CAGAGTG	323	UPRE-1
TERG_00107	ubiquitin-conjugating enzyme E2 N	CACCCTG (rc)	805	UPRE-1
TERG_00108	WD repeat protein (*T. equinum*)	CAGGGTG	933	UPRE-1
TERG_00109	thioesterase family protein (*A. benhamiae*)	CACTCTG (rc)	526	UPRE-1
TERG_00110	ubiquitin C-terminal hydrolase (*T. tonsurans*)	CACGCTG (rc)	164	UPRE-1
TERG_00111	ARP2/3 complex 20 kDa subunit (*T. tonsurans*)	CAGTGTG	411	UPRE-1
TERG_00112	hypothetical protein	CAGCGTG	167	UPRE-1
TERG_00114	hypothetical protein	CAGAGTG	849	UPRE-1
TERG_00119	CAMK/CAMKL/GIN4 protein kinase	CACCCTG (rc)	930	UPRE-1
TERG_00132	signal peptide peptidase (*T. tonsurans*)	CAGTGTG	194	UPRE-1
TERG_00151	hypothetical protein	CAGTGTG	277	UPRE-1
TERG_00153	actin associated protein Wsp1 (*T. equinum*)	CACACTG (rc)	209	UPRE-1
TERG_00160	GNAT family acetyltransferase, putative (*A. benhamiae*)	CACGCTG (rc)	389	UPRE-1

TERG_00163	siderochrome-iron transporter, putative (*A. benhamiae*)	CACACTG (rc)	934	UPRE-1
TERG_00164	FAD dependent sulfhydryl oxidase Erv2 (*T. tonsurans*)	CAGTGTG	281	UPRE-1
TERG_00172	hypothetical protein	CACGCTG (rc)	737	UPRE-1
TERG_00176	hydroxyisourate hydrolase	CAGCGTG	208	UPRE-1
TERG_00184	ribose-phosphate pyrophosphokinase (*T. tonsurans*)	CACCCTG (rc)	277	UPRE-1
TERG_00190	ribosomal protein S15	CAGTGTG	311	UPRE-1
TERG_00191	hypothetical protein	CAGCGTG	57	UPRE-1
TERG_00191	hypothetical protein	CAGCGTG	470	UPRE-1
TERG_00194	Rad2-like endonuclease, putative (*T. verrucosum*)	CACGCTG (rc)	680	UPRE-1
TERG_00195	hypothetical protein	CAGCGTG	828	UPRE-1
TERG_00196	hypothetical protein	CAGAGTG	720	UPRE-1
TERG_00197	aldose 1-epimerase family protein, putative (*T. verrucosum*)	CACACTG (rc)	447	UPRE-1
TERG_00198	AT DNA binding protein, putative (*T. verrucosum*)	CAGTGTG	339	UPRE-1
TERG_00211	proteasome subunit alpha type 6 (*T. equinum*)	CACCCTG (rc)	988	UPRE-1
TERG_00212	proteasome subunit alpha type-6 (*T. equinum*)	CAGTGTG	453	UPRE-1
TERG_00212	proteasome subunit alpha type-6 (*T. equinum*)	CAGGGTG	541	UPRE-1
TERG_00221	hypothetical protein	CACACTG (rc)	127	UPRE-1
TERG_00228	ThiJ/PfpI family protein (*A. benhamiae*)	CACACTG (rc)	439	UPRE-1
TERG_00230	RINT-1 family protein (*T. equinum*)	CAGTGTG	294	UPRE-1
TERG_00245	PHD finger domain protein, putative (*T. verrucosum*)	CACACTG (rc)	341	UPRE-1
TERG_00255	trimethyllysine dioxygenase	CAGCGTG	988	UPRE-1
TERG_00279	pre-mRNA splicing factor (*T. equinum*)	CAGGGTG	95	UPRE-1
TERG_00280	dihydroneopterin aldolase domain protein (*A. benhamiae*)	CAGCGTG	396	UPRE-1
TERG_00285	hypothetical protein	CAGTGTG	934	UPRE-1
TERG_00289	hypothetical protein	CACACTG (rc)	299	UPRE-1
TERG_00290	Ccr4-Not transcription complex subunit NOT1 (*T. tonsurans*)	CACCCTG (rc)	211	UPRE-1
TERG_00293	DASH complex subunit Duo1, putative (*T. verrucosum*)	CACACTG (rc)	655	UPRE-1
TERG_00296	hypothetical protein	CAGCGTG	164	UPRE-1
TERG_00297	galactokinase	CACGCTG (rc)	171	UPRE-1
TERG_00299	acetolactate synthase, large subunit, biosynthetic type	CAGCGTG	684	UPRE-1
TERG_00310	dolichol-phosphate mannosyltransferase (*T. tonsurans*)	CACACTG (rc)	628	UPRE-1
TERG_00310	dolichol-phosphate mannosyltransferase (*T. tonsurans*)	CACTCTG (rc)	720	UPRE-1
TERG_00311	hypothetical protein	CAGAGTG	707	UPRE-1
TERG_00311	hypothetical protein	CAGTGTG	799	UPRE-1
TERG_00357	SNARE-dependent exocytosis protein (*T. tonsurans*)	CAGAGTG	422	UPRE-1

TERG_00362	glutaminase (*T. equinum*)	CACTCTG (rc)	954	UPRE-1
TERG_00366	hypothetical protein	CAGCGTG	129	UPRE-1
TERG_00377	hypothetical protein	CAGGGTG	480	UPRE-1
TERG_00392	integral membrane protein (*T. equinum*)	CACACTG (rc)	490	UPRE-1
TERG_00412	CMGC/SRPK protein kinase (*T. tonsurans*)	CAGAGTG	619	UPRE-1
TERG_00416	phenylacetaldoxime dehydratase family protein, putative (*A. benhamiae*)	CAGAGTG	174	UPRE-1
TERG_00421	TBC domain protein, putative (*T. verrucosum*)	CAGCGTG	544	UPRE-1
TERG_00447	hypothetical protein	CACGCTG (rc)	130	UPRE-1
TERG_00449	mandelate racemase/muconate lactonizing enzyme family protein (*T. equinum*)	CAGCGTG	221	UPRE-1
TERG_00460	protein phosphatase PP2A regulatory subunit A (*T. equinum*)	CACGCTG (rc)	334	UPRE-1
TERG_00464	respiratory complex assembly protein Rmp1, putative (*T. verrucosum*)	CACTCTG (rc)	329	UPRE-1
TERG_00471	hypothetical protein	CAGAGTG	342	UPRE-1
TERG_00477	fungal specific transcription factor, putative (*T. verrucosum*)	CACGCTG (rc)	733	UPRE-1
TERG_00480	class I alpha-mannosidase (*T. tonsurans*)	CAGCGTG	510	UPRE-1
TERG_00483	hypothetical protein	CACTCTG (rc)	403	UPRE-1
TERG_00488	inositol phospholipid biosynthesis protein Scs3, putative (*A. benhamiae*)	CAGCGTG	44	UPRE-1
TERG_00512	hypothetical protein	CAGTGTG	712	UPRE-1
TERG_00524	trans-sialidase (*M. canis*)	CACTCTG (rc)	586	UPRE-1
TERG_00525	NAK protein kinase	CAGAGTG	494	UPRE-1
TERG_00528	hypothetical protein	CAGAGTG	11	UPRE-1
TERG_00528	hypothetical protein	CAGCGTG	291	UPRE-1
TERG_00528	hypothetical protein	CAGGGTG	607	UPRE-1
TERG_00535	calcium-translocating P-type ATPase, PMCA-type	CACACTG (rc)	970	UPRE-1
TERG_00539	F-box and WD40 domain-containing protein (*T. tonsurans*)	CAGAGTG	207	UPRE-1
TERG_00546	glutaredoxin	CAGTGTG	270	UPRE-1
TERG_00548	elongation factor 1-alpha	CACACTG (rc)	64	UPRE-1
TERG_00548	elongation factor 1-alpha	CACCCTG (rc)	632	UPRE-1
TERG_00553	amino acid permease (*T. equinum*)	CACTCTG (rc)	345	UPRE-1
TERG_00555	hypothetical protein	CAGAGTG	910	UPRE-1
TERG_00574	MFS multidrug transporter, putative (*T. verrucosum*)	CACCCTG (rc)	822	UPRE-1
TERG_00604	nucleolar protein (*T. equinum*)	CACTCTG (rc)	702	UPRE-1
TERG_00605	imidazoleglycerol-phosphate dehydratase (*T. equinum*)	CAGAGTG	446	UPRE-1

TERG_00606	alpha-1,6 mannosyltransferase subunit Mnn9 (*T. tonsurans*)	CACGCTG (rc)	229	UPRE-1
TERG_00606	alpha-1,6 mannosyltransferase subunit Mnn9 (*T. tonsurans*)	CACTCTG (rc)	879	UPRE-1
TERG_00613	hypothetical protein	CAGTGTG	774	UPRE-1
TERG_00615	HLH transcription factor, putative (*A. benhamiae*)	CAGGGTG	798	UPRE-1
TERG_00625	glycolipid anchored surface protein (GAS1) (*T. equinum*)	CAGGGTG	872	UPRE-1
TERG_00631	urate oxidase	CAGCGTG	108	UPRE-1
TERG_00632	ribosomal protein L13	CACGCTG (rc)	608	UPRE-1
TERG_00639	hypothetical protein	CACCCTG (rc)	726	UPRE-1
TERG_00639	hypothetical protein	CACACTG (rc)	856	UPRE-1
TERG_00649	DUF408 domain protein (*A. benhamiae*)	CAGCGTG	402	UPRE-1
TERG_00658	hypothetical protein	CACGCTG (rc)	684	UPRE-1
TERG_00669	C-5 sterol desaturase (*T. tonsurans*)	CACGCTG (rc)	614	UPRE-1
TERG_00674	actin (*T. tonsurans*)	CAGAGTG	286	UPRE-1
TERG_00683	hypothetical protein	CACTCTG (rc)	218	UPRE-1
TERG_00684	ubiquinol-cytochrome C chaperone (*T. equinum*)	CAGAGTG	670	UPRE-1
TERG_00694	glutamate 5-kinase	CACTCTG (rc)	765	UPRE-1
TERG_00696	WEE/WEE-UNCLASSIFIED protein kinase	CACGCTG (rc)	77	UPRE-1
TERG_00701	cytochrome b5, putative (*T. verrucosum*)	CAGCGTG	354	UPRE-1
TERG_00703	nuclear migration protein, putative (*A. benhamiae*)	CACACTG (rc)	503	UPRE-1
TERG_00707	GPI anchored serine-threonine rich protein (*T. verrucosum*)	CACGCTG (rc)	520	UPRE-1
TERG_00730	phospholipid metabolism enzyme regulator, putative (*T. verrucosum*)	CACCCTG (rc)	885	UPRE-1
TERG_00734	dipeptidase (*T. tonsurans*)	CACTCTG (rc)	375	UPRE-1
TERG_00734	dipeptidase (*T. tonsurans*)	CACTCTG (rc)	515	UPRE-1
TERG_00741	V-ATPase in vacuolar membrane protein 1 regulator (*T. equinum*)	CAGCGTG	581	UPRE-1
TERG_00754	hypothetical protein	CAGCGTG	649	UPRE-1
TERG_00763	hypothetical protein	CAGAGTG	327	UPRE-1
TERG_00776	MFS transporter (*T. tonsurans*)	CAGGGTG	136	UPRE-1
TERG_00783	guanine nucleotide-binding protein subunit beta-like protein	CACACTG (rc)	820	UPRE-1
TERG_00793	calponin domain-containing protein (*T. tonsurans*)	CAGTGTG	462	UPRE-1
TERG_00796	ORM1 (*M. gypseum*)	CACGCTG (rc)	946	UPRE-1
TERG_00798	zinc knuckle domain protein (*T. verrucosum*)	CAGCGTG	627	UPRE-1
TERG_00818	aldehyde dehydrogenase 3B1 (*T. equinum*)	CACACTG (rc)	728	UPRE-1
TERG_00828	hypothetical protein	CAGTGTG	414	UPRE-1

TERG_00857	polyketide synthase (*T. equinum*)	CAGTGTG	762	UPRE-1
TERG_00875	glutamate-cysteine ligase	CAGTGTG	690	UPRE-1
TERG_00881	zinc-containing alcohol dehydrogenase, putative (*T. verrucosum*)	CAGAGTG	127	UPRE-1
TERG_00892	hypothetical protein	CAGAGTG	167	UPRE-1
TERG_00896	hypothetical protein	CACTCTG (rc)	238	UPRE-1
TERG_00897	hypothetical protein	CAGAGTG	827	UPRE-1
TERG_00898	hypothetical protein	CACCCTG (rc)	326	UPRE-1
TERG_00906	cAMP-dependent protein kinase regulatory subunit	CACTCTG (rc)	268	UPRE-1
TERG_00908	extensin (*M. canis*)	CAGAGTG	691	UPRE-1
TERG_00914	hypothetical protein	CAGGGTG	92	UPRE-1
TERG_00916	MFS sugar permease, putative (*A. benhamiae*)	CAGCGTG	728	UPRE-1
TERG_00929	N-acetylglucosaminyl transferase component Gpi1 (*T. tonsurans*)	CACGCTG (rc)	335	UPRE-1
TERG_00936	integral membrane protein (*T. equinum*)	CACCCTG (rc)	214	UPRE-1
TERG_00938	RNA-binding protein (Nab3), putative (*A. benhamiae*)	CACTCTG (rc)	553	UPRE-1
TERG_00939	peptidyl-tRNA hydrolase domain protein (*T. verrucosum*)	CAGAGTG	916	UPRE-1
TERG_00949	extracellular proline rich protein (*T. verrucosum*)	CAGAGTG	682	UPRE-1
TERG_00958	amino acid permease (*T. tonsurans*)	CACCCTG (rc)	110	UPRE-1
TERG_00959	RNA binding protein, putative (*A. benhamiae*)	CACACTG (rc)	100	UPRE-1
TERG_00959	RNA binding protein, putative (*A. benhamiae*)	CACCCTG (rc)	378	UPRE-1
TERG_00960	histone acetyltransferase (*T. equinum*)	CAGGGTG	988	UPRE-1
TERG_00981	hypothetical protein	CAGAGTG	924	UPRE-1
TERG_00983	eukaryotic translation initiation factor 3 subunit K	CAGGGTG	233	UPRE-1
TERG_00999	hypothetical protein	CACCCTG (rc)	331	UPRE-1
TERG_01000	hypothetical protein	CACCCTG (rc)	985	UPRE-1
TERG_01001	GDP-mannose transporter	CAGGGTG	698	UPRE-1
TERG_01004	extracellular sialidase/neuraminidase (*T. equinum*)	CAGAGTG	15	UPRE-1
TERG_01016	hypothetical protein	CACACTG (rc)	120	UPRE-1
TERG_01016	hypothetical protein	CACCCTG (rc)	751	UPRE-1
TERG_01017	cytoplasm to vacuole targeting Vps64 (*T. equinum*)	CAGGGTG	754	UPRE-1
TERG_01050	metal homeostatis protein bsd2 (*T. tonsurans*)	CACCCTG (rc)	315	UPRE-1
TERG_01051	hypothetical protein	CAGTGTG	259	UPRE-1
TERG_01053	MFS monocarboxylate transporter, putative (*T. verrucosum*)	CAGAGTG	95	UPRE-1
TERG_01053	MFS monocarboxylate transporter, putative (*T. verrucosum*)	CAGTGTG	201	UPRE-1
TERG_01056	PH domain-containing protein (*T. equinum*)	CAGCGTG	702	UPRE-1

TERG_01081	hypothetical protein	CAGAGTG	411	UPRE-1
TERG_01095	hypothetical protein	CACGCTG (rc)	892	UPRE-1
TERG_01101	hypothetical protein	CAGAGTG	186	UPRE-1
TERG_01107	anthranilate synthase component I (*T. equinum*)	CACCCTG (rc)	590	UPRE-1
TERG_01107	anthranilate synthase component I (*T. equinum*)	CACCCTG (rc)	895	UPRE-1
TERG_01108	hypothetical protein	CAGGGTG	730	UPRE-1
TERG_01119	BolA domain-containing protein (*T. tonsurans*)	CACACTG (rc)	101	UPRE-1
TERG_01125	SprT family metallopeptidase, putative (*T. verrucosum*)	CACACTG (rc)	635	UPRE-1
TERG_01126	1,3-beta-glucanosyltransferase, putative (*T. verrucosum*)	CACGCTG (rc)	31	UPRE-1
TERG_01130	hypothetical protein	CACTCTG (rc)	277	UPRE-1
TERG_01134	metalloprotease MEP1 (*A. benhamiae*)	CAGAGTG	116	UPRE-1
TERG_01134	metalloprotease MEP1 (*A. benhamiae*)	CAGAGTG	845	UPRE-1
TERG_01150	hypothetical protein	CACACTG (rc)	639	UPRE-1
TERG_01151	26S proteasome non-ATPase regulatory subunit 13 (*T. equinum*)	CAGTGTG	604	UPRE-1
TERG_01156	integral membrane protein (*T. equinum*)	CAGTGTG	326	UPRE-1
TERG_01159	hypothetical protein	CACGCTG (rc)	654	UPRE-1
TERG_01160	ubiquitin C-terminal hydrolase Ubp8, putative (*A. benhamiae*)	CAGCGTG	293	UPRE-1
TERG_01164	beta-alanine synthase, putative (*T. verrucosum*)	CAGAGTG	219	UPRE-1
TERG_01175	LovB-like polyketide synthase, putative (*A. benhamiae*)	CACTCTG (rc)	705	UPRE-1
TERG_01183	RNase3 domain-containing protein (*T. equinum*)	CACGCTG (rc)	253	UPRE-1
TERG_01183	RNase3 domain-containing protein (*T. equinum*)	CACCCTG (rc)	283	UPRE-1
TERG_01183	RNase3 domain-containing protein (*T. equinum*)	CACCCTG (rc)	782	UPRE-1
TERG_01186	SWI/SNF family DNA-dependent ATPase	CAGTGTG	443	UPRE-1
TERG_01191	NADPH oxidase (*T. equinum*)	CACTCTG (rc)	553	UPRE-1
TERG_01193	PRO41 protein (*T. equinum*)	CAGAGTG	784	UPRE-1
TERG_01197	nitrilase (*T. tonsurans*)	CACTCTG (rc)	968	UPRE-1
TERG_01198	pre-mRNA-splicing factor rse1	CACGCTG (rc)	251	UPRE-1
TERG_01203	75k gamma secalin (*M. canis*)	CACTCTG (rc)	868	UPRE-1
TERG_01204	acetyl-CoA acetyltransferase (*T. tonsurans*)	CACCCTG (rc)	684	UPRE-1
TERG_01208	phosphoethanolamine transferase class O (*T. equinum*)	CACGCTG (rc)	683	UPRE-1
TERG_01226	GAS2 domain-containing protein (*T. equinum*)	CACCCTG (rc)	239	UPRE-1
TERG_01227	cell cycle checkpoint protein (*T. tonsurans*)	CAGGGTG	869	UPRE-1
TERG_01228	COPII vesicle coat protein Sec16, putative (*A. benhamiae*)	CACACTG (rc)	564	UPRE-1
TERG_01240	secreted protein (*T. equinum*)	CACCCTG (rc)	833	UPRE-1
TERG_01249	hypothetical protein	CACTCTG (rc)	572	UPRE-1

TERG_01265	HhH-GPD family base excision DNA repair protein (*T. tonsurans*)	CACTCTG (rc)	388	UPRE-1
TERG_01269	2-methylcitrate dehydratase	CAGTGTG	26	UPRE-1
TERG_01273	alpha-1,2-mannosidase, putative subfamily (*T. verrucosum*)	CACTCTG (rc)	928	UPRE-1
TERG_01275	hypothetical protein	CACTCTG (rc)	758	UPRE-1
TERG_01290	N-acetyl-gamma-glutamyl-phosphate reductase	CAGGGTG	627	UPRE-1
TERG_01310	nascent polypeptide-associated complex subunit beta	CACCCTG (rc)	453	UPRE-1
TERG_01318	hypothetical protein	CACCCTG (rc)	315	UPRE-1
TERG_01323	WW domain binding protein (*T. equinum*)	CACACTG (rc)	250	UPRE-1
TERG_01325	hypothetical protein	CAGCGTG	913	UPRE-1
TERG_01326	hypothetical protein	CAGAGTG	603	UPRE-1
TERG_01328	metalloreductase transmembrane component, putative (*A. benhamiae*)	CACTCTG (rc)	457	UPRE-1
TERG_01340	hypothetical protein	CAGTGTG	110	UPRE-1
TERG_01347	TIGR01456 family HAD hydrolase	CAGTGTG	282	UPRE-1
TERG_01355	DUF1713 domain-containing protein (*T. equinum*)	CACACTG (rc)	182	UPRE-1
TERG_01359	transketolase	CACGCTG (rc)	372	UPRE-1
TERG_01363	SET domain-containing protein (*T. equinum*)	CACACTG (rc)	236	UPRE-1
TERG_01364	hypothetical protein	CAGTGTG	573	UPRE-1
TERG_01369	ubiquinol-cytochrome C reductase complex subunit UcrQ (*T. tonsurans*)	CACTCTG (rc)	517	UPRE-1
TERG_01377	hypothetical protein	CACCCTG (rc)	169	UPRE-1
TERG_01379	serine/threonine-protein phosphatase 2A activator 2 (*T. equinum*)	CAGGGTG	182	UPRE-1
TERG_01383	dienelactone hydrolase (*T. tonsurans*)	CACCCTG (rc)	737	UPRE-1
TERG_01408	hypothetical protein	CACTCTG (rc)	516	UPRE-1
TERG_01409	F-box and WD domain-containing protein (*T. equinum*)	CAGAGTG	602	UPRE-1
TERG_01447	bcp1 (*T. equinum*)	CAGAGTG	529	UPRE-1
TERG_01448	chorismate mutase	CACGCTG (rc)	284	UPRE-1
TERG_01448	chorismate mutase	CACTCTG (rc)	894	UPRE-1
TERG_01449	mitochondrial division protein 1	CAGAGTG	248	UPRE-1
TERG_01449	mitochondrial division protein 1	CAGCGTG	858	UPRE-1
TERG_01473	Pro-apoptotic serine protease NMA111	CAGAGTG	183	UPRE-1
TERG_01473	Pro-apoptotic serine protease NMA111	CAGTGTG	452	UPRE-1
TERG_01473	Pro-apoptotic serine protease NMA111	CAGAGTG	589	UPRE-1
TERG_01481	MFS multidrug transporter, putative (*A. benhamiae*)	CACCCTG (rc)	432	UPRE-1
TERG_01488	hypothetical protein	CACCCTG (rc)	560	UPRE-1

TERG_01502	U3 small nucleolar ribonucleoprotein Lcp5 (*T. equinum*)	CACTCTG (rc)	443	UPRE-1
TERG_01503	DUF895 domain membrane protein (*T. verrucosum*)	CACTCTG (rc)	484	UPRE-1
TERG_01509	hypothetical protein	CACGCTG (rc)	752	UPRE-1
TERG_01514	beta-1,4-mannosyltransferase (*T. tonsurans*)	CACGCTG (rc)	47	UPRE-1
TERG_01515	CDF zinc transporter (Msc2), putative (*T. verrucosum*)	CAGCGTG	853	UPRE-1
TERG_01519	hypothetical protein	CAGTGTG	393	UPRE-1
TERG_01543	s-adenosylmethionine (SAM)-dependent methyltransferase (*M. gypseum*)	CAGCGTG	508	UPRE-1
TERG_01549	N-acetyltransferase 9 (*T. tonsurans*)	CAGGGTG	379	UPRE-1
TERG_01565	clathrin heavy chain 1 (*T. equinum*)	CACTCTG (rc)	488	UPRE-1
TERG_01569	hypothetical protein	CACCCTG (rc)	75	UPRE-1
TERG_01588	MAK11 (*T. equinum*)	CACTCTG (rc)	606	UPRE-1
TERG_01589	MYB and HSA domain protein (*T. verrucosum*)	CAGAGTG	909	UPRE-1
TERG_01590	pre-mRNA-splicing factor spp2 (*T. equinum*)	CAGTGTG	697	UPRE-1
TERG_01602	proteasome component PUP3	CACCCTG (rc)	83	UPRE-1
TERG_01606	peptidase, putative (*A. benhamiae*)	CAGAGTG	966	UPRE-1
TERG_01627	hypothetical protein	CAGTGTG	50	UPRE-1
TERG_01645	mitochondrial carrier protein (*T. tonsurans*)	CACTCTG (rc)	895	UPRE-1
TERG_01646	proteasome component (Ecm29), putative (*A. benhamiae*)	CAGAGTG	847	UPRE-1
TERG_01649	lipase, putative (*A. benhamiae*)	CACGCTG (rc)	897	UPRE-1
TERG_01650	Arp2/3 complex chain sop2 (*T. equinum*)	CACCCTG (rc)	24	UPRE-1
TERG_01651	U1 small nuclear ribonucleoprotein A (*T. equinum*)	CAGTGTG	81	UPRE-1
TERG_01652	Atg10p (*M. gypseum*)	CACCCTG (rc)	365	UPRE-1
TERG_01665	pre-rRNA-processing protein PNO1	CACCCTG (rc)	593	UPRE-1
TERG_01677	glutamine-fructose-6-phosphate transaminase (isomerizing)	CACTCTG (rc)	305	UPRE-1
TERG_01694	ankyrin repeat-containing protein (*T. equinum*)	CACACTG (rc)	559	UPRE-1
TERG_01708	ARF GTPase activator (*T. tonsurans*)	CACCCTG (rc)	188	UPRE-1
TERG_01709	hypothetical protein	CAGAGTG	236	UPRE-1
TERG_01734	hypothetical protein	CACCCTG (rc)	969	UPRE-1
TERG_01737	NSDC (*T. equinum*)	CAGCGTG	79	UPRE-1
TERG_01738	hypothetical protein	CACCCTG (rc)	402	UPRE-1
TERG_01755	hypothetical protein	CACCCTG (rc)	724	UPRE-1
TERG_01781	endo-1,3(4)-beta-glucanase, putative (*A. benhamiae*)	CAGAGTG	467	UPRE-1
TERG_01801	hypothetical protein	CAGCGTG	662	UPRE-1
TERG_01815	origin recognition complex subunit 3, putative (*A. benhamiae*)	CAGTGTG	643	UPRE-1

TERG_01816	translocation protein (*T. tonsurans*)	CACGCTG (rc)	855	UPRE-1
TERG_01817	AAA family ATPase (*T. tonsurans*)	CACCCTG (rc)	421	UPRE-1
TERG_01824	DNA-binding protein SMUBP-2 (*T. equinum*)	CACGCTG (rc)	25	UPRE-1
TERG_01824	DNA-binding protein SMUBP-2 (*T. equinum*)	CACGCTG (rc)	172	UPRE-1
TERG_01826	ubiquitin-conjugating enzyme (*T. tonsurans*)	CAGCGTG	226	UPRE-1
TERG_01826	ubiquitin-conjugating enzyme (*T. tonsurans*)	CAGCGTG	373	UPRE-1
TERG_01833	peroxisomal membrane protein (*T. tonsurans*)	CAGTGTG	564	UPRE-1
TERG_01837	glycosyl hydrolase (*T. equinum*)	CACCCTG (rc)	11	UPRE-1
TERG_01838	LIM domain protein (*A. benhamiae*)	CACACTG (rc)	515	UPRE-1
TERG_01844	hypothetical protein	CAGCGTG	511	UPRE-1
TERG_01845	amidase family protein, putative (*T. verrucosum*)	CACCCTG (rc)	290	UPRE-1
TERG_01848	integral membrane protein (*M. gypseum*)	CACACTG (rc)	380	UPRE-1
TERG_01867	hypothetical protein	CACTCTG (rc)	207	UPRE-1
TERG_01867	hypothetical protein	CACGCTG (rc)	527	UPRE-1
TERG_01867	hypothetical protein	CACTCTG (rc)	649	UPRE-1
TERG_01872	polyketide synthase, putative (*T. verrucosum*)	CACTCTG (rc)	499	UPRE-1
TERG_01889	RING finger domain-containing protein (*T. tonsurans*)	CACGCTG (rc)	24	UPRE-1
TERG_01892	alanyl-tRNA synthetase	CACCCTG (rc)	866	UPRE-1
TERG_01893	hypothetical protein	CAGGGTG	554	UPRE-1
TERG_01894	hypothetical protein	CACTCTG (rc)	804	UPRE-1
TERG_01896	hypothetical protein	CACACTG (rc)	3	UPRE-1
TERG_01897	oryzin (*T. equinum*)	CAGTGTG	412	UPRE-1
TERG_01905	AMP deaminase	CAGTGTG	527	UPRE-1
TERG_01912	MFS transporter (*T. equinum*)	CAGCGTG	74	UPRE-1
TERG_01915	O-methyltransferase (*A. benhamiae*)	CACACTG (rc)	283	UPRE-1
TERG_01915	O-methyltransferase (*A. benhamiae*)	CACACTG (rc)	597	UPRE-1
TERG_01916	TdiA protein (*T. equinum*)	CACTCTG (rc)	993	UPRE-1
TERG_01917	O-methyltransferase, putative (*A. benhamiae*)	CAGAGTG	457	UPRE-1
TERG_01925	hypothetical protein	CACTCTG (rc)	674	UPRE-1
TERG_01937	short chain dehydrogenase (*T. tonsurans*)	CAGGGTG	386	UPRE-1
TERG_01950	serine/threonine protein kinase (*T. tonsurans*)	CACTCTG (rc)	18	UPRE-1
TERG_01950	serine/threonine protein kinase (*T. tonsurans*)	CACTCTG (rc)	689	UPRE-1
TERG_01951	SET and MYND domain protein, putative (*A. benhamiae*)	CAGAGTG	211	UPRE-1
TERG_01951	SET and MYND domain protein, putative (*A. benhamiae*)	CAGAGTG	882	UPRE-1
TERG_01959	RNA polymerase Rpb1 C-terminal repeat domain-containing protein (*T. equinum*)	CACGCTG (rc)	45	UPRE-1

TERG_01967	GPI anchored protein, putative (*T. verrucosum*)	CAGGGTG	267	UPRE-1
TERG_01973	mRNA cleavage and polyadenylation specificity factor complex subunit (Pta1), putative (*A. benhamiae*)	CAGTGTG	185	UPRE-1
TERG_01980	hypothetical protein	CAGCGTG	724	UPRE-1
TERG_01980	hypothetical protein	CAGCGTG	775	UPRE-1
TERG_01981	hypothetical protein	CACGCTG (rc)	125	UPRE-1
TERG_02004	ureidoglycolate hydrolase, putative (*T. verrucosum*)	CACTCTG (rc)	780	UPRE-1
TERG_02005	catalase (*T. tonsurans*)	CAGAGTG	574	UPRE-1
TERG_02008	hypothetical protein	CACTCTG (rc)	8	UPRE-1
TERG_02009	short chain oxidoreductase (CsgA), putative (*T. verrucosum*)	CAGAGTG	746	UPRE-1
TERG_02055	hypothetical protein	CACCCTG (rc)	279	UPRE-1
TERG_02058	ribosome biogenesis protein RPF2 (*T. equinum*)	CACTCTG (rc)	827	UPRE-1
TERG_02074	26S protease regulatory subunit 7	CACGCTG (rc)	230	UPRE-1
TERG_02077	hypothetical protein	CAGGGTG	412	UPRE-1
TERG_02085	hypothetical protein	CAGGGTG	232	UPRE-1
TERG_02086	exocyst complex component Sec10 (*T. tonsurans*)	CACGCTG (rc)	146	UPRE-1
TERG_02092	hypothetical protein	CAGGGTG	950	UPRE-1
TERG_02099	uracil permease (*T. equinum*)	CAGCGTG	103	UPRE-1
TERG_02132	5-histidylcysteine sulfoxide synthase	CACGCTG (rc)	486	UPRE-1
TERG_02133	fluG protein (*T. equinum*)	CAGCGTG	205	UPRE-1
TERG_02139	eukaryotic translation initiation factor 3 subunit D	CACCCTG (rc)	859	UPRE-1
TERG_02140	bZIP transcription factor (*T. tonsurans*)	CACTCTG (rc)	57	UPRE-1
TERG_02140	bZIP transcription factor (*T. tonsurans*)	CACGCTG (rc)	789	UPRE-1
TERG_02147	3-demethylubiquinone-9 3-methyltransferase, putative (*T. verrucosum*)	CACACTG (rc)	451	UPRE-1
TERG_02151	hypothetical protein	CACGCTG (rc)	559	UPRE-1
TERG_02152	hypothetical protein	CAGCGTG	966	UPRE-1
TERG_02155	type I phosphodiesterase/nucleotide pyrophosphatase (*T. tonsurans*)	CACGCTG (rc)	221	UPRE-1
TERG_02164	indoleamine 2,3-dioxygenase (*T. equinum*)	CACTCTG (rc)	96	UPRE-1
TERG_02167	phosphotransferase enzyme family protein (*T. equinum*)	CACTCTG (rc)	209	UPRE-1
TERG_02168	hypothetical protein	CACCCTG (rc)	139	UPRE-1
TERG_02169	carboxylesterase, putative (*A. benhamiae*)	CACTCTG (rc)	465	UPRE-1
TERG_02172	cytochrome P450 monooxygenase (*T. equinum*)	CACGCTG (rc)	277	UPRE-1
TERG_02172	cytochrome P450 monooxygenase (*T. equinum*)	CACCCTG (rc)	322	UPRE-1
TERG_02185	hypothetical protein	CAGTGTG	318	UPRE-1
TERG_02188	mechanosensitive ion channel family protein (*T. tonsurans*)	CACACTG (rc)	543	UPRE-1

TERG_02194	exonuclease (*T. tonsurans*)	CAGGGTG	305	UPRE-1
TERG_02202	NADH-ubiquinone oxidoreductase 12 kDa subunit, mitochondrial	CAGTGTG	453	UPRE-1
TERG_02212	short chain dehydrogenase/reductase family protein (*T. verrucosum*)	CAGGGTG	757	UPRE-1
TERG_02214	carboxypeptidase 2	CAGCGTG	362	UPRE-1
TERG_02226	DUF453 domain-containing protein (*T. equinum*)	CAGAGTG	216	UPRE-1
TERG_02243	hypothetical protein	CACACTG (rc)	522	UPRE-1
TERG_02244	MFS transporter, putative (*A. benhamiae*)	CAGTGTG	285	UPRE-1
TERG_02250	cyclopropane-fatty-acyl-phospholipid synthase (*T. tonsurans*)	CACTCTG (rc)	41	UPRE-1
TERG_02250	cyclopropane-fatty-acyl-phospholipid synthase (*T. tonsurans*)	CACCCTG (rc)	564	UPRE-1
TERG_02275	hypothetical protein	CACACTG (rc)	633	UPRE-1
TERG_02282	hypothetical protein	CAGAGTG	419	UPRE-1
TERG_02287	calcium dependent mitochondrial carrier protein (*T. tonsurans*)	CACACTG (rc)	173	UPRE-1
TERG_02297	TRAF-like signal transducer, putative (*A. benhamiae*)	CAGCGTG	886	UPRE-1
TERG_02299	HET-C domain-containing protein (*T. tonsurans*)	CACGCTG (rc)	685	UPRE-1
TERG_02300	hypothetical protein	CAGCGTG	116	UPRE-1
TERG_02314	cofactor for methionyl- and glutamyl-tRNA synthetases, putative (*T. verrucosum*)	CACGCTG (rc)	47	UPRE-1
TERG_02326	WD domain-containing protein (*T. tonsurans*)	CACGCTG (rc)	97	UPRE-1
TERG_02336	hypothetical protein	CACCCTG (rc)	143	UPRE-1
TERG_02336	hypothetical protein	CACTCTG (rc)	178	UPRE-1
TERG_02337	multidomain presynaptic cytomatrix related protein (*T. verrucosum*)	CAGAGTG	908	UPRE-1
TERG_02337	multidomain presynaptic cytomatrix related protein (*T. verrucosum*)	CAGGGTG	943	UPRE-1
TERG_02341	DEAD box helicase (*T. tonsurans*)	CACCCTG (rc)	800	UPRE-1
TERG_02357	amino acid permease (*T. equinum*)	CACCCTG (rc)	913	UPRE-1
TERG_02361	5'-nucleotidase, putative (*A. benhamiae*)	CACGCTG (rc)	96	UPRE-1
TERG_02362	hypothetical protein	CACGCTG (rc)	536	UPRE-1
TERG_02362	hypothetical protein	CACGCTG (rc)	992	UPRE-1
TERG_02363	succinate-semialdehyde dehydrogenase (*T. tonsurans*)	CAGCGTG	45	UPRE-1
TERG_02367	hypothetical protein	CAGGGTG	94	UPRE-1
TERG_02367	hypothetical protein	CAGAGTG	762	UPRE-1
TERG_02368	extracellular developmental signal biosynthesis protein FluG (*A. benhamiae*)	CAGCGTG	315	UPRE-1
TERG_02380	hypothetical protein	CACCCTG (rc)	656	UPRE-1

TERG_02384	hypothetical protein	CAGCGTG	523	UPRE-1
TERG_02404	40S ribosomal protein S4	CAGCGTG	578	UPRE-1
TERG_02405	mating locus protein, putative (*T. verrucosum*)	CACTCTG (rc)	160	UPRE-1
TERG_02408	DNA lyase (*T. tonsurans*)	CACACTG (rc)	459	UPRE-1
TERG_02409	ABcox13, cytochrome c oxidase subunit VIa, putative (*A. benhamiae*)	CAGAGTG	238	UPRE-1
TERG_02413	hypothetical protein	CACCCTG (rc)	293	UPRE-1
TERG_02417	anaphase-promoting complex protein (*T. tonsurans*)	CAGCGTG	577	UPRE-1
TERG_02418	translation initiation factor SUI1	CAGTGTG	376	UPRE-1
TERG_02424	rho2 (*T. equinum*)	CAGCGTG	822	UPRE-1
TERG_02438	hypothetical protein	CACTCTG (rc)	77	UPRE-1
TERG_02438	hypothetical protein	CACGCTG (rc)	134	UPRE-1
TERG_02444	hypothetical protein	CACCCTG (rc)	190	UPRE-1
TERG_02451	lipoyl synthase, mitochondrial	CACGCTG (rc)	3	UPRE-1
TERG_02456	siroheme synthase (*T. tonsurans*)	CACCCTG (rc)	377	UPRE-1
TERG_02467	pyridoxamine phosphate oxidase (*T. tonsurans*)	CAGGGTG	414	UPRE-1
TERG_02468	ubiquitin thiolesterase (OtuB1), putative (*T. verrucosum*)	CACTCTG (rc)	219	UPRE-1
TERG_02492	ubiquitin-conjugating enzyme E2-16 kDa	CAGAGTG	233	UPRE-1
TERG_02498	CMGC/SRPK protein kinase	CAGGGTG	45	UPRE-1
TERG_02500	phosphatidylinositol-4-phosphate 5-kinase (*T. tonsurans*)	CAGCGTG	322	UPRE-1
TERG_02505	MFS transporter, putative (*A. benhamiae*)	CAGTGTG	194	UPRE-1
TERG_02507	transcription factor Rba50 (*T. tonsurans*)	CAGTGTG	684	UPRE-1
TERG_02512	hypothetical protein	CAGTGTG	659	UPRE-1
TERG_02513	TAM domain methyltransferase (*T. equinum*)	CACACTG (rc)	601	UPRE-1
TERG_02515	STE/STE7/MEK1 protein kinase	CACGCTG (rc)	989	UPRE-1
TERG_02526	hypothetical protein	CAGCGTG	756	UPRE-1
TERG_02527	WD40 repeat protein (*A. benhamiae*)	CAGAGTG	754	UPRE-1
TERG_02527	WD40 repeat protein (*A. benhamiae*)	CAGTGTG	858	UPRE-1
TERG_02535	nicotinate-nucleotide diphosphorylase (carboxylating)	CAGCGTG	178	UPRE-1
TERG_02541	FAD-dependent monooxygenase, putative (*T. verrucosum*)	CACTCTG (rc)	469	UPRE-1
TERG_02541	FAD-dependent monooxygenase, putative (*T. verrucosum*)	CACGCTG (rc)	734	UPRE-1
TERG_02550	ZZ type zinc finger domain protein (*T. verrucosum*)	CACCCTG (rc)	689	UPRE-1
TERG_02563	esterase family protein (*A. benhamiae*)	CAGTGTG	821	UPRE-1
TERG_02566	hypothetical protein	CAGTGTG	172	UPRE-1
TERG_02567	phytanoyl-CoA dioxygenase (*T. tonsurans*)	CAGCGTG	114	UPRE-1

TERG_02567	phytanoyl-CoA dioxygenase (*T. tonsurans*)	CAGGGTG	980	UPRE-1
TERG_02578	nuclear protein (*T. tonsurans*)	CACCCTG (rc)	611	UPRE-1
TERG_02584	hypothetical protein	CACCCTG (rc)	264	UPRE-1
TERG_02584	hypothetical protein	CACACTG (rc)	475	UPRE-1
TERG_02588	hypothetical protein	CAGAGTG	194	UPRE-1
TERG_02590	peptidyl-prolyl cis-trans isomerase D	CACCCTG (rc)	435	UPRE-1
TERG_02593	C2H2 finger domain protein, putative (*T. verrucosum*)	CAGTGTG	60	UPRE-1
TERG_02601	Phosphoglucomutase (*T. tonsurans*)	CACCCTG (rc)	470	UPRE-1
TERG_02605	CCCH zinc finger domain-containing protein (*T. equinum*)	CACCCTG (rc)	875	UPRE-1
TERG_02609	actin patches distal protein 1 (*T. equinum*)	CAGCGTG	379	UPRE-1
TERG_02615	oxidoreductase, 2OG-Fe(II) oxygenase family, putative (*A. benhamiae*)	CAGTGTG	510	UPRE-1
TERG_02625	hypothetical protein	CACCCTG (rc)	228	UPRE-1
TERG_02626	RING finger domain protein (*A. benhamiae*)	CAGGGTG	357	UPRE-1
TERG_02626	RING finger domain protein (*A. benhamiae*)	CAGTGTG	707	UPRE-1
TERG_02634	C2H2 finger domain protein, putative (*A. benhamiae*)	CACCCTG (rc)	512	UPRE-1
TERG_02635	hypothetical protein	CAGGGTG	682	UPRE-1
TERG_02636	F-box protein (*T. tonsurans*)	CACCCTG (rc)	544	UPRE-1
TERG_02657	phosphoglycerate mutase (*T. equinum*)	CACACTG (rc)	26	UPRE-1
TERG_02658	CAMK/CAMKL/AMPK protein kinase	CAGTGTG	935	UPRE-1
TERG_02659	non-classical export protein Nce102, putative (*A. benhamiae*)	CAGCGTG	585	UPRE-1
TERG_02659	non-classical export protein Nce102, putative (*A. benhamiae*)	CAGCGTG	668	UPRE-1
TERG_02665	FunK1 protein kinase (*M. gypseum*)	CAGCGTG	312	UPRE-1
TERG_02669	pre-mRNA splicing factor (*T. equinum*)	CACCCTG (rc)	379	UPRE-1
TERG_02688	hypothetical protein	CAGTGTG	992	UPRE-1
TERG_02696	hypothetical protein	CAGCGTG	387	UPRE-1
TERG_02699	protein kinase, putative (*T. verrucosum*)	CAGGGTG	797	UPRE-1
TERG_02750	protoporphyrinogen oxidase	CAGGGTG	515	UPRE-1
TERG_02753	E3 ubiquitin-protein ligase ubr1 (*T. equinum*)	CAGCGTG	429	UPRE-1
TERG_02772	DNA polymerase V (*T. tonsurans*)	CACGCTG (rc)	192	UPRE-1
TERG_02773	tartrate dehydrogenase	CAGCGTG	105	UPRE-1
TERG_02778	SCF ubiquitin ligase subunit CulC (*T. tonsurans*)	CACGCTG (rc)	200	UPRE-1
TERG_02780	HEAT repeat protein (*T. tonsurans*)	CACACTG (rc)	92	UPRE-1
TERG_02798	DUF895 domain membrane protein (*T. equinum*)	CACGCTG (rc)	949	UPRE-1
TERG_02801	hypothetical protein	CACGCTG (rc)	22	UPRE-1

TERG_02803	3-oxoacyl-(acyl-carrier-protein) reductase (*T. verrucosum*)	CAGGGTG	578	UPRE-1
TERG_02816	dienelactone hydrolase (*T. equinum*)	CACACTG (rc)	238	UPRE-1
TERG_02817	hypothetical protein	CAGGGTG	443	UPRE-1
TERG_02854	dynein light chain (*T. tonsurans*)	CACCCTG (rc)	172	UPRE-1
TERG_02863	protein kinase activator (*T. tonsurans*)	CACACTG (rc)	572	UPRE-1
TERG_02863	protein kinase activator (*T. tonsurans*)	CACCCTG (rc)	764	UPRE-1
TERG_02864	hypothetical protein	CAGGGTG	782	UPRE-1
TERG_02864	hypothetical protein	CAGTGTG	974	UPRE-1
TERG_02867	ribosome biogenesis ATPase RIX7 (*T. tonsurans*)	CACTCTG (rc)	28	UPRE-1
TERG_02869	DUF1000 domain-containing protein (*T. equinum*)	CACGCTG (rc)	97	UPRE-1
TERG_02878	eukaryotic translation initiation factor 3 subunit F	CAGAGTG	516	UPRE-1
TERG_02879	transcription initiation factor TFIID subunit 13 (*T. equinum*)	CACCCTG (rc)	399	UPRE-1
TERG_02887	aminotransferase (*T. tonsurans*)	CAGAGTG	247	UPRE-1
TERG_02901	N-acetyltransferase complex ARD1 subunit (*T. tonsurans*)	CAGGGTG	19	UPRE-1
TERG_02906	PAB1 binding protein (*T. tonsurans*)	CACCCTG (rc)	581	UPRE-1
TERG_02907	actin-interacting protein (*T. tonsurans*)	CAGGGTG	567	UPRE-1
TERG_02915	ESCRT-II complex component (*T. tonsurans*)	CAGGGTG	373	UPRE-1
TERG_02922	phosphoserine phosphatase (*T. tonsurans*)	CACACTG (rc)	99	UPRE-1
TERG_02923	protein kinase subdomain-containing protein (*T. equinum*)	CAGCGTG	989	UPRE-1
TERG_02927	hypothetical protein	CACTCTG (rc)	86	UPRE-1
TERG_02938	vacuolar membrane protein (*T. equinum*)	CACGCTG (rc)	933	UPRE-1
TERG_02939	Ser/Thr protein phosphatase (*T. tonsurans*)	CAGCGTG	178	UPRE-1
TERG_02941	hypothetical protein	CAGGGTG	19	UPRE-1
TERG_02949	54S ribosomal protein L6 (*T. equinum*)	CAGAGTG	161	UPRE-1
TERG_02951	VHS domain protein (*A. benhamiae*)	CAGAGTG	543	UPRE-1
TERG_02953	hypothetical protein	CACGCTG (rc)	172	UPRE-1
TERG_02959	hypothetical protein	CAGGGTG	779	UPRE-1
TERG_02966	PHD finger domain protein, putative (*A. benhamiae*)	CAGAGTG	694	UPRE-1
TERG_02966	PHD finger domain protein, putative (*A. benhamiae*)	CAGAGTG	992	UPRE-1
TERG_02973	morphogenesis protein (Msb1), putative (*T. verrucosum*)	CAGTGTG	994	UPRE-1
TERG_02987	mitochondrial import inner membrane translocase subunit tim-17	CAGCGTG	278	UPRE-1
TERG_02988	asparaginase (*T. tonsurans*)	CAGGGTG	527	UPRE-1
TERG_02996	hypothetical protein	CAGGGTG	106	UPRE-1
TERG_03003	autophagy protein Apg12 (*T. equinum*)	CAGTGTG	160	UPRE-1

TERG_03011	MFS transporter, putative (*A. benhamiae*)	CACACTG (rc)	172	UPRE-1
TERG_03013	pyridoxal-5'-phosphate-dependent enzyme (*T. equinum*)	CACTCTG (rc)	546	UPRE-1
TERG_03014	ER membrane protein Wsc4 (*T. equinum*)	CACCCTG (rc)	985	UPRE-1
TERG_03024	hypothetical protein	CAGTGTG	195	UPRE-1
TERG_03026	WD repeat protein (*T. tonsurans*)	CAGGGTG	690	UPRE-1
TERG_03027	hypothetical protein	CACTCTG (rc)	683	UPRE-1
TERG_03028	hypothetical protein	CAGAGTG	250	UPRE-1
TERG_03028	hypothetical protein	CAGAGTG	662	UPRE-1
TERG_03033	BTB domain and ankyrin repeat protein (*A. benhamiae*)	CAGCGTG	185	UPRE-1
TERG_03048	adenylate cyclase (*T. tonsurans*)	CACGCTG (rc)	928	UPRE-1
TERG_03053	serine/threonine protein kinase (*T. tonsurans*)	CAGTGTG	151	UPRE-1
TERG_03066	MFS peptide transporter, putative (*T. verrucosum*)	CACCCTG (rc)	789	UPRE-1
TERG_03078	cytochrome P450 oxidoreductase OrdA-like, putative (*T. verrucosum*)	CACTCTG (rc)	649	UPRE-1
TERG_03081	intracellular protein transporter (*T. equinum*)	CACGCTG (rc)	96	UPRE-1
TERG_03081	intracellular protein transporter (*T. equinum*)	CACCCTG (rc)	727	UPRE-1
TERG_03090	hypothetical protein	CAGCGTG	355	UPRE-1
TERG_03102	sterol 24-C-methyltransferase (*T. equinum*)	CAGAGTG	409	UPRE-1
TERG_03105	hypothetical protein	CAGGGTG	654	UPRE-1
TERG_03120	conserved glutamic acid-rich protein (*A. benhamiae*)	CACGCTG (rc)	842	UPRE-1
TERG_03131	alpha-1,2-mannosyltransferase (*T. equinum*)	CACTCTG (rc)	368	UPRE-1
TERG_03132	hypothetical protein	CAGCGTG	398	UPRE-1
TERG_03139	hypothetical protein	CACTCTG (rc)	108	UPRE-1
TERG_03151	import inner membrane translocase subunit tim-21 (*T. equinum*)	CACTCTG (rc)	988	UPRE-1
TERG_03152	arf GTPase-activating protein (*T. equinum*)	CAGAGTG	854	UPRE-1
TERG_03153	dihydropteroate synthase	CACGCTG (rc)	826	UPRE-1
TERG_03154	2-nitropropane dioxygenase (*M. gypseum*)	CAGCGTG	202	UPRE-1
TERG_03160	hypothetical protein	CAGCGTG	871	UPRE-1
TERG_03179	NlpC/P60-like cell-wall peptidase, putative (*A. benhamiae*)	CAGCGTG	372	UPRE-1
TERG_03201	CMGC/DYRK/YAK protein kinase (*T. tonsurans*)	CAGCGTG	193	UPRE-1
TERG_03205	DNA polymerase alpha catalytic subunit (*T. equinum*)	CAGAGTG	184	UPRE-1
TERG_03206	hsp7-like protein	CACACTG (rc)	56	UPRE-1
TERG_03214	hypothetical protein	CAGCGTG	927	UPRE-1
TERG_03225	betaine aldehyde dehydrogenase, putative (*A. benhamiae*)	CAGAGTG	880	UPRE-1
TERG_03230	hypothetical protein	CACACTG (rc)	575	UPRE-1

TERG_03231	cytochrome P450 52A12 (*T. tonsurans*)	CAGTGTG	947	UPRE-1
TERG_03240	transmembrane efflux protein (*T. tonsurans*)	CACTCTG (rc)	206	UPRE-1
TERG_03241	hypothetical protein	CAGGGTG	211	UPRE-1
TERG_03248	extracellular metalloproteinase 3	CAGAGTG	631	UPRE-1
TERG_03254	hypothetical protein	CAGTGTG	25	UPRE-1
TERG_03256	carbonate dehydratase (*T. equinum*)	CAGAGTG	169	UPRE-1
TERG_03256	carbonate dehydratase (*T. equinum*)	CAGTGTG	187	UPRE-1
TERG_03261	pre-mRNA splicing factor prp1 (*T. tonsurans*)	CACGCTG (rc)	84	UPRE-1
TERG_03267	oligomeric Golgi complex component 4 (*T. equinum*)	CACGCTG (rc)	18	UPRE-1
TERG_03269	hexokinase	CAGCGTG	5	UPRE-1
TERG_03292	NADH-ubiquinone dehydrogenase 24 kDa subunit (*T. tonsurans*)	CACTCTG (rc)	512	UPRE-1
TERG_03313	fucose-specific lectin FleA (*A. benhamiae*)	CAGTGTG	740	UPRE-1
TERG_03314	TfdA family oxidoreductase, putative (*T. verrucosum*)	CACCCTG (rc)	79	UPRE-1
TERG_03316	hypothetical protein	CACGCTG (rc)	903	UPRE-1
TERG_03318	N(5)-glutamine methyltransferase MTQ2 (*T. equinum*)	CAGCGTG	53	UPRE-1
TERG_03319	small nuclear ribonucleoprotein Sm D1 (*T. tonsurans*)	CACACTG (rc)	713	UPRE-1
TERG_03320	hypothetical protein	CAGTGTG	212	UPRE-1
TERG_03327	ER membrane protein (Pkr1), putative (*T. verrucosum*)	CAGCGTG	987	UPRE-1
TERG_03331	F-box protein (*T. equinum*)	CACTCTG (rc)	307	UPRE-1
TERG_03352	tyrosine decarboxylase (*T. equinum*)	CACTCTG (rc)	371	UPRE-1
TERG_03369	fungal specific transcription factor domain-containing protein (*T. equinum*)	CACCCTG (rc)	514	UPRE-1
TERG_03389	orotidine 5'-phosphate decarboxylase	CAGAGTG	664	UPRE-1
TERG_03397	hypothetical protein	CACTCTG (rc)	480	UPRE-1
TERG_03398	chitinase (*M. canis*)	CAGGGTG	570	UPRE-1
TERG_03401	high affinity nickel transporter (*T. tonsurans*)	CACACTG (rc)	581	UPRE-1
TERG_03402	zinc carboxypeptidase, putative (*A. benhamiae*)	CACGCTG (rc)	786	UPRE-1
TERG_03414	acetyl-CoA carboxylase (*T. equinum*)	CAGTGTG	460	UPRE-1
TERG_03426	hypothetical protein	CACTCTG (rc)	64	UPRE-1
TERG_03428	peptidyl-prolyl cis-trans isomerase (*T. tonsurans*)	CAGAGTG	297	UPRE-1
TERG_03438	MFS monocarboxylate transporter (*T. equinum*)	CACACTG (rc)	947	UPRE-1
TERG_03441	hypothetical protein	CAGAGTG	428	UPRE-1
TERG_03452	HEAT repeat protein (*T. equinum*)	CAGAGTG	510	UPRE-1
TERG_03458	hypothetical protein	CACACTG (rc)	577	UPRE-1
TERG_03458	hypothetical protein	CACTCTG (rc)	739	UPRE-1
TERG_03458	hypothetical protein	CACTCTG (rc)	887	UPRE-1
TERG_03464	stress response protein (*T. equinum*)	CACCCTG (rc)	547	UPRE-1

TERG_03465	pre-mRNA-splicing factor cwc15	CACCCTG (rc)	269	UPRE-1
TERG_03477	DUF1674 domain-containing protein (*T. equinum*)	CACACTG (rc)	955	UPRE-1
TERG_03478	class III aminotransferase, putative (*A. benhamiae*)	CAGTGTG	744	UPRE-1
TERG_03481	hypothetical protein	CAGCGTG	663	UPRE-1
TERG_03485	NADPH-adrenodoxin reductase (*T. tonsurans*)	CAGAGTG	864	UPRE-1
TERG_03493	hypothetical protein	CACGCTG (rc)	36	UPRE-1
TERG_03493	hypothetical protein	CACTCTG (rc)	191	UPRE-1
TERG_03509	hypothetical protein	CACGCTG (rc)	599	UPRE-1
TERG_03510	peroxisomal membrane anchor protein, putative (*A. benhamiae*)	CAGCGTG	94	UPRE-1
TERG_03511	calcineurin binding protein (*T. equinum*)	CACTCTG (rc)	315	UPRE-1
TERG_03511	calcineurin binding protein (*T. equinum*)	CACTCTG (rc)	645	UPRE-1
TERG_03512	hypothetical protein	CAGGGTG	887	UPRE-1
TERG_03526	hypothetical protein	CACCCTG (rc)	735	UPRE-1
TERG_03528	cytochrome c	CACCCTG (rc)	345	UPRE-1
TERG_03530	3' exoribonuclease (*T. tonsurans*)	CACCCTG (rc)	79	UPRE-1
TERG_03530	3' exoribonuclease (*T. tonsurans*)	CACGCTG (rc)	478	UPRE-1
TERG_03556	transcriptional regulator Medusa (*A. benhamiae*)	CACGCTG (rc)	178	UPRE-1
TERG_03562	hypothetical protein	CAGCGTG	503	UPRE-1
TERG_03569	RING finger domain-containing protein (*T. equinum*)	CACGCTG (rc)	678	UPRE-1
TERG_03571	kinetochore protein spc24 (*M. canis*)	CAGGGTG	200	UPRE-1
TERG_03571	kinetochore protein spc24 (*M. canis*)	CAGCGTG	623	UPRE-1
TERG_03577	GATA transcription factor (*T. tonsurans*)	CAGAGTG	300	UPRE-1
TERG_03578	nitrogen regulatory protein DAL80	CAGAGTG	764	UPRE-1
TERG_03582	hypothetical protein	CACTCTG (rc)	192	UPRE-1
TERG_03582	hypothetical protein	CACTCTG (rc)	432	UPRE-1
TERG_03604	replication factor C subunit 5	CAGTGTG	179	UPRE-1
TERG_03605	ribosomal protein L16	CACACTG (rc)	733	UPRE-1
TERG_03606	replication factor C protein (*T. tonsurans*)	CAGTGTG	236	UPRE-1
TERG_03609	endoplasmic reticulum protein (*M. canis*)	CAGCGTG	692	UPRE-1
TERG_03631	sister chromatid cohesion factor (*T. equinum*)	CACACTG (rc)	657	UPRE-1
TERG_03632	hypothetical protein	CAGTGTG	519	UPRE-1
TERG_03636	ankyrin repeat protein (*T. equinum*)	CACACTG (rc)	678	UPRE-1
TERG_03636	ankyrin repeat protein (*T. equinum*)	CACGCTG (rc)	691	UPRE-1
TERG_03640	peptidase M28 (*T. tonsurans*)	CAGAGTG	9	UPRE-1
TERG_03647	hypothetical protein	CACCCTG (rc)	232	UPRE-1
TERG_03652	chromosome segregation protein sudA	CAGCGTG	133	UPRE-1

TERG_03661	hypothetical protein	CACGCTG (rc)	320	UPRE-1
TERG_03661	hypothetical protein	CACTCTG (rc)	701	UPRE-1
TERG_03661	hypothetical protein	CACTCTG (rc)	943	UPRE-1
TERG_03663	hypothetical protein	CACCCTG (rc)	36	UPRE-1
TERG_03672	hypothetical protein	CAGCGTG	254	UPRE-1
TERG_03674	EBP domain-containing protein (*T. tonsurans*)	CACACTG (rc)	131	UPRE-1
TERG_03674	EBP domain-containing protein (*T. tonsurans*)	CACTCTG (rc)	471	UPRE-1
TERG_03695	pyrroline-5-carboxylate reductase	CACGCTG (rc)	737	UPRE-1
TERG_03696	CTD kinase subunit gamma (*T. equinum*)	CAGCGTG	817	UPRE-1
TERG_03704	integral membrane protein Pth11-like, putative (*T. verrucosum*)	CACGCTG (rc)	292	UPRE-1
TERG_03706	trichodiene oxygenase (*T. equinum*)	CACGCTG (rc)	734	UPRE-1
TERG_03707	geranylgeranyl diphosphate synthase, putative (*A. benhamiae*)	CAGGGTG	3	UPRE-1
TERG_03707	geranylgeranyl diphosphate synthase, putative (*A. benhamiae*)	CAGCGTG	299	UPRE-1
TERG_03709	hypothetical protein	CACGCTG (rc)	696	UPRE-1
TERG_03710	hypothetical protein	CAGGGTG	393	UPRE-1
TERG_03710	hypothetical protein	CAGCGTG	972	UPRE-1
TERG_03711	histone acetyltransferase type B catalytic subunit (*T. equinum*)	CACTCTG (rc)	485	UPRE-1
TERG_03711	histone acetyltransferase type B catalytic subunit (*T. equinum*)	CACGCTG (rc)	958	UPRE-1
TERG_03715	zinc transporter SLC39A9 (*T. equinum*)	CAGCGTG	211	UPRE-1
TERG_03719	MFS sugar transporter (*T. tonsurans*)	CACACTG (rc)	727	UPRE-1
TERG_03724	RING finger domain-containing protein (*T. equinum*)	CACCCTG (rc)	272	UPRE-1
TERG_03739	hypothetical protein	CAGCGTG	128	UPRE-1
TERG_03740	beta-1,6 glucan synthetase (*T. tonsurans*)	CAGAGTG	587	UPRE-1
TERG_03744	hypothetical protein	CAGCGTG	367	UPRE-1
TERG_03750	small nuclear ribonucleoprotein (*T. tonsurans*)	CACCCTG (rc)	498	UPRE-1
TERG_03775	fructose-2,6-bisphosphatase (*T. tonsurans*)	CACGCTG (rc)	198	UPRE-1
TERG_03789	glutamine-serine rich protein MS8, putative (*A. benhamiae*)	CAGGGTG	221	UPRE-1
TERG_03790	DUF1275 domain-containing protein (*T. equinum*)	CAGCGTG	136	UPRE-1
TERG_03796	sorting nexin 41 (*T. tonsurans*)	CACCCTG (rc)	708	UPRE-1
TERG_03800	hypothetical protein	CAGCGTG	139	UPRE-1
TERG_03801	CAMK protein kinase	CAGAGTG	654	UPRE-1
TERG_03807	hypothetical protein	CACCCTG (rc)	187	UPRE-1
TERG_03811	actin-binding protein (*T. equinum*)	CACTCTG (rc)	57	UPRE-1
TERG_03812	kinesin motor domain-containing protein (*T. tonsurans*)	CAGAGTG	80	UPRE-1

TERG_03833	isochorismatase family protein (*A. benhamiae*)	CACCCTG (rc)	921	UPRE-1
TERG_03834	ketol-acid reductoisomerase, mitochondrial	CAGGGTG	179	UPRE-1
TERG_03840	bromodomain associated domain protein (*A. benhamiae*)	CACGCTG (rc)	199	UPRE-1
TERG_03850	hypothetical protein	CAGAGTG	733	UPRE-1
TERG_03854	laccase (*T. tonsurans*)	CAGGGTG	299	UPRE-1
TERG_03865	actin cytoskeleton-regulatory complex protein END3	CACCCTG (rc)	741	UPRE-1
TERG_03879	cell wall biogenesis protein Mhp1, putative (*T. verrucosum*)	CACGCTG (rc)	126	UPRE-1
TERG_03879	cell wall biogenesis protein Mhp1, putative (*T. verrucosum*)	CACCCTG (rc)	322	UPRE-1
TERG_03892	CAMK/CAMKL protein kinase (*T. tonsurans*)	CAGAGTG	508	UPRE-1
TERG_03895	lipase/thioesterase family protein (*A. benhamiae*)	CACGCTG (rc)	984	UPRE-1
TERG_03899	hypothetical protein	CACGCTG (rc)	166	UPRE-1
TERG_03903	DUF6 domain-containing protein (*T. equinum*)	CACACTG (rc)	796	UPRE-1
TERG_03905	tubulin gamma chain	CAGAGTG	634	UPRE-1
TERG_03907	neutral amino acid permease (*T. tonsurans*)	CAGAGTG	754	UPRE-1
TERG_03916	geranylgeranyl pyrophosphate synthetase (*T. tonsurans*)	CACTCTG (rc)	196	UPRE-1
TERG_03917	carotene cyclase (*T. equinum*)	CAGAGTG	984	UPRE-1
TERG_03919	phytoene dehydrogenase (*T. equinum*)	CAGAGTG	991	UPRE-1
TERG_03933	ABC metal ion transporter (*T. equinum*)	CACCCTG (rc)	166	UPRE-1
TERG_03935	hypothetical protein	CACACTG (rc)	811	UPRE-1
TERG_03936	CAMK protein kinase	CAGTGTG	567	UPRE-1
TERG_03938	hypothetical protein	CAGGGTG	567	UPRE-1
TERG_03938	hypothetical protein	CAGGGTG	852	UPRE-1
TERG_03944	hypothetical protein	CACCCTG (rc)	34	UPRE-1
TERG_03949	equisetin synthetase (*T. equinum*)	CAGCGTG	630	UPRE-1
TERG_03959	myo-inositol oxygenase (*T. tonsurans*)	CACACTG (rc)	381	UPRE-1
TERG_03963	mannosyl phosphorylinositol ceramide synthase SUR1 (*T. equinum*)	CAGGGTG	631	UPRE-1
TERG_03973	zinc finger protein gcs1 (*T. equinum*)	CACGCTG (rc)	475	UPRE-1
TERG_03977	hypothetical protein	CACGCTG (rc)	474	UPRE-1
TERG_03985	hypothetical protein	CACGCTG (rc)	818	UPRE-1
TERG_03989	cyclin (*T. tonsurans*)	CACACTG (rc)	951	UPRE-1
TERG_03991	phosphotransferase enzyme family protein (*T. verrucosum*)	CACCCTG (rc)	216	UPRE-1
TERG_03992	26S protease regulatory subunit 4 (*M. gypseum*)	CAGAGTG	77	UPRE-1
TERG_04001	NADH-ubiquinone oxidoreductase B12 subunit (*T. tonsurans*)	CAGCGTG	307	UPRE-1

TERG_04002	ribosome assembly protein SQT1 (*T. equinum*)	CACCCTG (rc)	389	UPRE-1
TERG_04006	Rho1 guanine nucleotide exchange factor 3 (*T. equinum*)	CAGAGTG	69	UPRE-1
TERG_04023	hypothetical protein	CACACTG (rc)	290	UPRE-1
TERG_04031	BAR domain protein (*A. benhamiae*)	CACACTG (rc)	487	UPRE-1
TERG_04036	adenosine kinase (*T. tonsurans*)	CAGTGTG	389	UPRE-1
TERG_04044	vacuolar protein sorting protein DigA (*T. tonsurans*)	CACTCTG (rc)	540	UPRE-1
TERG_04045	SNF2 family helicase/ATPase (*T. tonsurans*)	CAGAGTG	207	UPRE-1
TERG_04065	filamentation protein (Rhf1), putative (*T. verrucosum*)	CACACTG (rc)	394	UPRE-1
TERG_04069	hypothetical protein	CAGTGTG	792	UPRE-1
TERG_04072	SNF2 family helicase/ATPase, putative (*T. verrucosum*)	CACTCTG (rc)	212	UPRE-1
TERG_04073	glutathione synthetase	CAGAGTG	507	UPRE-1
TERG_04080	transcription elongation factor spt4	CACTCTG (rc)	68	UPRE-1
TERG_04096	SUMO activating enzyme (*T. tonsurans*)	CAGCGTG	396	UPRE-1
TERG_04098	Swi5 domain-containing protein (*T. equinum*)	CAGTGTG	458	UPRE-1
TERG_04101	G-protein coupled receptor (*T. equinum*)	CACCCTG (rc)	201	UPRE-1
TERG_04102	hypothetical protein	CAGGGTG	740	UPRE-1
TERG_04104	autophagocytosis protein (*T. tonsurans*)	CACCCTG (rc)	828	UPRE-1
TERG_04115	aldo-keto reductase, putative (*T. verrucosum*)	CACGCTG (rc)	329	UPRE-1
TERG_04115	aldo-keto reductase, putative (*T. verrucosum*)	CACGCTG (rc)	655	UPRE-1
TERG_04116	hypothetical protein	CAGCGTG	386	UPRE-1
TERG_04116	hypothetical protein	CAGCGTG	712	UPRE-1
TERG_04123	hypothetical protein	CAGAGTG	158	UPRE-1
TERG_04124	hypothetical protein	CAGAGTG	228	UPRE-1
TERG_04126	dual specificity phosphatase (*T. equinum*)	CACCCTG (rc)	524	UPRE-1
TERG_04127	hypothetical protein	CAGGGTG	766	UPRE-1
TERG_04132	hypothetical protein	CAGAGTG	320	UPRE-1
TERG_04133	major allergen and cytotoxin AspF1 (*A. benhamiae*)	CACACTG (rc)	947	UPRE-1
TERG_04143	Cytochrome c oxidase polypeptide V (*T. tonsurans*)	CAGCGTG	529	UPRE-1
TERG_04146	FAD/FMN-containing isoamyl alcohol oxidase MreA-like, putative (*A. benhamiae*)	CAGTGTG	697	UPRE-1
TERG_04151	GTP-binding protein ypt3 (*T. tonsurans*)	CAGCGTG	390	UPRE-1
TERG_04154	hypothetical protein	CACACTG (rc)	37	UPRE-1
TERG_04156	hypothetical protein	CAGGGTG	754	UPRE-1
TERG_04178	1,3-beta-glucan biosynthesis protein (*T. tonsurans*)	CACCCTG (rc)	417	UPRE-1
TERG_04184	CorA family metal ion transporter, putative (*A. benhamiae*)	CAGAGTG	841	UPRE-1
TERG_04200	hypothetical protein	CAGCGTG	440	UPRE-1

TERG_04201	histone-lysine N-methyltransferase, H3 lysine-36 specific	CACCCTG (rc)	705	UPRE-1
TERG_04208	HAL protein kinase	CACGCTG (rc)	143	UPRE-1
TERG_04221	H/ACA ribonucleoprotein complex subunit 3	CAGCGTG	543	UPRE-1
TERG_04222	art-4 protein (*M. canis*)	CACGCTG (rc)	253	UPRE-1
TERG_04233	hypothetical protein	CAGCGTG	643	UPRE-1
TERG_04234	hydrophobin, putative (*T. verrucosum*)	CACACTG (rc)	712	UPRE-1
TERG_04235	WW domain-containing protein (*T. tonsurans*)	CAGTGTG	534	UPRE-1
TERG_04258	histone acetylase complex subunit Paf400 (*T. equinum*)	CAGGGTG	263	UPRE-1
TERG_04259	mRNA splicing protein (*T. tonsurans*)	CACTCTG (rc)	173	UPRE-1
TERG_04263	hypothetical protein	CAGCGTG	714	UPRE-1
TERG_04281	woronin body major protein	CAGTGTG	581	UPRE-1
TERG_04291	urea amidolyase, putative (*A. benhamiae*)	CACCCTG (rc)	895	UPRE-1
TERG_04292	hypothetical protein	CAGGGTG	787	UPRE-1
TERG_04294	methylcrotonoyl-CoA carboxylase subunit beta (*T. tonsurans*)	CAGTGTG	94	UPRE-1
TERG_04294	methylcrotonoyl-CoA carboxylase subunit beta (*T. tonsurans*)	CAGTGTG	201	UPRE-1
TERG_04294	methylcrotonoyl-CoA carboxylase subunit beta (*T. tonsurans*)	CAGGGTG	491	UPRE-1
TERG_04297	ab-hydrolase associated lipase, putative (*A. benhamiae*)	CACCCTG (rc)	232	UPRE-1
TERG_04297	ab-hydrolase associated lipase, putative (*A. benhamiae*)	CACCCTG (rc)	359	UPRE-1
TERG_04299	dolichyl-diphosphooligosaccharide-protein glycotransferase (*T. tonsurans*)	CACGCTG (rc)	814	UPRE-1
TERG_04300	NADH-ubiquinone oxidoreductase 9.5 kDa subunit (*T. tonsurans*)	CAGCGTG	732	UPRE-1
TERG_04301	transcription factor RfeG (*T. tonsurans*)	CACCCTG (rc)	90	UPRE-1
TERG_04303	hypothetical protein	CACTCTG (rc)	613	UPRE-1
TERG_04309	multidrug resistance protein (*T. equinum*)	CACACTG (rc)	904	UPRE-1
TERG_04311	mitochondrial phosphate carrier protein (*T. tonsurans*)	CAGGGTG	833	UPRE-1
TERG_04318	hypothetical protein	CACTCTG (rc)	853	UPRE-1
TERG_04325	ubiquitin conjugating enzyme, putative (*T. verrucosum*)	CAGCGTG	649	UPRE-1
TERG_04330	DUF1620 domain-containing protein (*T. equinum*)	CAGTGTG	934	UPRE-1
TERG_04332	phosphatidylinositol transporter (*T. tonsurans*)	CACTCTG (rc)	116	UPRE-1
TERG_04334	fungal specific transcription factor, putative (*A. benhamiae*)	CACTCTG (rc)	597	UPRE-1
TERG_04346	HSP70 family protein (*A. benhamiae*)	CACCCTG (rc)	981	UPRE-1
TERG_04350	hypothetical protein	CACTCTG (rc)	978	UPRE-1
TERG_04360	RNA binding protein (*T. equinum*)	CAGCGTG	190	UPRE-1

TERG_04362	condensin complex component cnd3 (*T. equinum*)	CACCCTG (rc)	118	UPRE-1
TERG_04364	sulfite oxidase (*T. equinum*)	CACTCTG (rc)	480	UPRE-1
TERG_04367	hypothetical protein	CACCCTG (rc)	976	UPRE-1
TERG_04369	hypothetical protein	CAGGGTG	105	UPRE-1
TERG_04375	D-lactate dehydrogenase (*T. tonsurans*)	CACACTG (rc)	400	UPRE-1
TERG_04386	hypothetical protein	CACTCTG (rc)	498	UPRE-1
TERG_04396	a-pheromone processing metallopeptidase Ste23 (*T. equinum*)	CACCCTG (rc)	198	UPRE-1
TERG_04406	heat shock transcription factor Hsf1, putative (*T. verrucosum*)	CACCCTG (rc)	195	UPRE-1
TERG_04415	CMGC/SRPK protein kinase	CAGCGTG	47	UPRE-1
TERG_04418	tRNA (adenine-N(1)-)-methyltransferase non-catalytic subunit trm6	CAGTGTG	4	UPRE-1
TERG_04424	MYB DNA-binding domain protein (*A. benhamiae*)	CAGGGTG	845	UPRE-1
TERG_04438	GDP-mannose transporter 1	CAGGGTG	648	UPRE-1
TERG_04446	eukaryotic peptide chain release factor subunit 1	CACTCTG (rc)	683	UPRE-1
TERG_04446	eukaryotic peptide chain release factor subunit 1	CACGCTG (rc)	830	UPRE-1
TERG_04447	protein kinase subdomain-containing protein (*T. equinum*)	CAGCGTG	670	UPRE-1
TERG_04447	protein kinase subdomain-containing protein (*T. equinum*)	CAGAGTG	817	UPRE-1
TERG_04448	CMGC/DYRK protein kinase (*M. gypseum*)	CAGCGTG	14	UPRE-1
TERG_04448	CMGC/DYRK protein kinase (*M. gypseum*)	CAGAGTG	161	UPRE-1
TERG_04456	DUF1671 domain-containing protein (*T. equinum*)	CAGGGTG	832	UPRE-1
TERG_04457	POS9-activating factor FAP7 (*T. tonsurans*)	CAGGGTG	706	UPRE-1
TERG_04461	P-type ATPase (*T. equinum*)	CAGCGTG	975	UPRE-1
TERG_04469	D-lactate dehydrogenase, putative (*T. verrucosum*)	CACGCTG (rc)	157	UPRE-1
TERG_04480	C6 transcription factor, putative (*A. benhamiae*)	CAGCGTG	417	UPRE-1
TERG_04483	Mitochondrial ribosomal subunit S27 (*T. tonsurans*)	CAGTGTG	250	UPRE-1
TERG_04487	hypothetical protein	CACGCTG (rc)	284	UPRE-1
TERG_04490	NADH-ubiquinone oxidoreductase 78 kDa subunit, mitochondrial	CACACTG (rc)	593	UPRE-1
TERG_04494	hypothetical protein	CACTCTG (rc)	992	UPRE-1
TERG_04501	WD repeat protein (*T. equinum*)	CAGAGTG	528	UPRE-1
TERG_04506	60S ribosomal protein L4-A (*T. tonsurans*)	CAGAGTG	458	UPRE-1
TERG_04518	histone acetyltransferase type B subunit 2	CAGTGTG	785	UPRE-1
TERG_04538	pyruvate dehydrogenase kinase (*T. tonsurans*)	CAGCGTG	289	UPRE-1
TERG_04541	Ulp1 protease family protein (*T. verrucosum*)	CACCCTG (rc)	737	UPRE-1
TERG_04551	hypothetical protein	CAGAGTG	738	UPRE-1
TERG_04558	nucleoside diphosphate kinase	CACGCTG (rc)	696	UPRE-1

TERG_04570	NSFL1 cofactor p47 (*T. equinum*)	CACCCTG (rc)	277	UPRE-1
TERG_04572	mitochondrial export translocase Oxa1, putative (*T. verrucosum*)	CAGGGTG	205	UPRE-1
TERG_04580	NADP-specific glutamate dehydrogenase	CAGAGTG	777	UPRE-1
TERG_04596	SUMO-conjugating enzyme ubc9	CACTCTG (rc)	934	UPRE-1
TERG_04601	hypothetical protein	CACGCTG (rc)	810	UPRE-1
TERG_04608	C6 transcription factor, putative (*A. benhamiae*)	CAGCGTG	681	UPRE-1
TERG_04612	branched-chain amino acid aminotransferase	CACGCTG (rc)	413	UPRE-1
TERG_04622	ankyrin repeat protein nuc-2 (*T. equinum*)	CACGCTG (rc)	117	UPRE-1
TERG_04635	JmjC domain-containing protein (*T. equinum*)	CACGCTG (rc)	937	UPRE-1
TERG_04636	4-hydroxybenzoate polyprenyl transferase	CAGCGTG	458	UPRE-1
TERG_04650	iron sulfur cluster assembly protein 1, mitochondrial	CACCCTG (rc)	614	UPRE-1
TERG_04652	uracil-DNA glycosylase	CACGCTG (rc)	252	UPRE-1
TERG_04653	hypothetical protein	CAGCGTG	123	UPRE-1
TERG_04661	yippee family protein (*T. tonsurans*)	CACGCTG (rc)	106	UPRE-1
TERG_04662	protein disulfide-isomerase domain-containing protein	CAGTGTG	296	UPRE-1
TERG_04666	hypothetical protein	CAGAGTG	269	UPRE-1
TERG_04666	hypothetical protein	CAGCGTG	519	UPRE-1
TERG_04669	hypothetical protein	CAGAGTG	597	UPRE-1
TERG_04670	CHY and RING finger domain protein, putative (*A. benhamiae*)	CAGCGTG	741	UPRE-1
TERG_04677	guanylate kinase	CACGCTG (rc)	319	UPRE-1
TERG_04682	shugoshin family protein (*T. verrucosum*)	CAGTGTG	496	UPRE-1
TERG_04691	nuclear division Rft1 protein, putative (*T. verrucosum*)	CACGCTG (rc)	971	UPRE-1
TERG_04692	replication protein A DNA-binding subunit (*T. tonsurans*)	CAGCGTG	596	UPRE-1
TERG_04703	DUF1715 domain-containing protein (*T. equinum*)	CAGCGTG	139	UPRE-1
TERG_04703	DUF1715 domain-containing protein (*T. equinum*)	CAGTGTG	353	UPRE-1
TERG_04709	glucose-6-phosphate 1-dehydrogenase	CACGCTG (rc)	701	UPRE-1
TERG_04713	cystathionine beta-synthase (*T. tonsurans*)	CACCCTG (rc)	382	UPRE-1
TERG_04746	nucleolar GTP-binding protein (*T. tonsurans*)	CAGAGTG	473	UPRE-1
TERG_04747	phosphatidylglycerol/phosphatidylinositol transfer protein (*T. equinum*)	CAGGGTG	219	UPRE-1
TERG_04758	SAGA complex subunit Ada2 (*T. tonsurans*)	CACTCTG (rc)	467	UPRE-1
TERG_04759	AGC/AKT protein kinase	CAGAGTG	121	UPRE-1
TERG_04765	MFS transporter, putative (*A. benhamiae*)	CAGGGTG	431	UPRE-1
TERG_04771	ribonuclease T2 family, putative (*A. benhamiae*)	CAGGGTG	749	UPRE-1
TERG_04782	37S ribosomal protein Mrp10 (*T. equinum*)	CACCCTG (rc)	445	UPRE-1

TERG_04784	hypothetical protein	CACACTG (rc)	441	UPRE-1
TERG_04785	hypothetical protein	CAGTGTG	737	UPRE-1
TERG_04795	HIT finger domain-containing protein (*T. equinum*)	CAGGGTG	110	UPRE-1
TERG_04806	ABC transporter (*T. tonsurans*)	CAGTGTG	862	UPRE-1
TERG_04819	Fe superoxide dismutase (*T. tonsurans*)	CACCCTG (rc)	233	UPRE-1
TERG_04820	hypothetical protein	CAGGGTG	978	UPRE-1
TERG_04825	hypothetical protein	CACTCTG (rc)	433	UPRE-1
TERG_04826	actin binding protein (*T. equinum*)	CAGGGTG	297	UPRE-1
TERG_04826	actin binding protein (*T. equinum*)	CAGAGTG	982	UPRE-1
TERG_04847	hypothetical protein	CACCCTG (rc)	35	UPRE-1
TERG_04848	ABC transporter, putative (*T. verrucosum*)	CAGGGTG	935	UPRE-1
TERG_04851	Acyl CoA binding protein family protein (*A. benhamiae*)	CACCCTG (rc)	602	UPRE-1
TERG_04856	triosephosphate isomerase (*T. tonsurans*)	CACCCTG (rc)	408	UPRE-1
TERG_04857	ribose 5-phosphate isomerase	CAGGGTG	717	UPRE-1
TERG_04894	alcohol dehydrogenase IV (*T. equinum*)	CAGTGTG	394	UPRE-1
TERG_04920	KH domain-containing protein (*T. tonsurans*)	CACCCTG (rc)	982	UPRE-1
TERG_04927	fermentation associated protein (Csf1), putative (*A. benhamiae*)	CAGCGTG	137	UPRE-1
TERG_04936	meiotic chromosome segregation protein (*T. equinum*)	CAGCGTG	198	UPRE-1
TERG_04948	hypothetical protein	CAGAGTG	117	UPRE-1
TERG_04950	cytochrome P450 monooxygenase (*T. equinum*)	CACTCTG (rc)	304	UPRE-1
TERG_04979	RNA exonuclease 4 (*T. tonsurans*)	CACGCTG (rc)	212	UPRE-1
TERG_04990	deoxyhypusine hydroxylase	CACGCTG (rc)	621	UPRE-1
TERG_04991	NADH-ubiquinone oxidoreductase 64 kDa subunit (*T. tonsurans*)	CAGCGTG	185	UPRE-1
TERG_04998	mitochondrial ATP-dependent RNA helicase (*T. tonsurans*)	CACCCTG (rc)	477	UPRE-1
TERG_04999	Sec1 family protein (*T. tonsurans*)	CAGGGTG	641	UPRE-1
TERG_05002	UDP-glucose 4-epimerase GalE	CACCCTG (rc)	408	UPRE-1
TERG_05003	NmrA family protein (*T. equinum*)	CAGGGTG	223	UPRE-1
TERG_05008	hypothetical protein	CACTCTG (rc)	951	UPRE-1
TERG_05009	Dishevelled, Egl-10, and Pleckstrin domain protein (*A. benhamiae*)	CAGAGTG	529	UPRE-1
TERG_05037	hypothetical protein	CACCCTG (rc)	100	UPRE-1
TERG_05057	DUF775 domain-containing protein (*T. equinum*)	CACGCTG (rc)	213	UPRE-1
TERG_05058	thiamine pyrophosphokinase (*T. tonsurans*)	CAGCGTG	946	UPRE-1
TERG_05067	hypothetical protein	CACCCTG (rc)	942	UPRE-1
TERG_05077	D-amino-acid oxidase (*T. equinum*)	CACACTG (rc)	628	UPRE-1

TERG_05083	adaptin ear-binding coat-associated protein 1 (*M. canis*)	CACTCTG (rc)	564	UPRE-1
TERG_05084	translation initiation factor eIF-2B alpha subunit (*T. tonsurans*)	CACACTG (rc)	209	UPRE-1
TERG_05104	MFS transporter of unknown specificity (*T. verrucosum*)	CAGAGTG	870	UPRE-1
TERG_05105	hypothetical protein	CACTCTG (rc)	364	UPRE-1
TERG_05106	integral membrane protein (*T. equinum*)	CAGAGTG	815	UPRE-1
TERG_05118	actin cytoskeleton organization protein (*T. tonsurans*)	CAGTGTG	52	UPRE-1
TERG_05124	gliotoxin biosynthesis protein GliK (*T. verrucosum*)	CACGCTG (rc)	802	UPRE-1
TERG_05127	aminotransferase GliI (*A. benhamiae*)	CAGGGTG	170	UPRE-1
TERG_05146	protein methyltransferase (*T. tonsurans*)	CACACTG (rc)	261	UPRE-1
TERG_05147	pre-mRNA-splicing factor ATP-dependent RNA helicase PRP43	CAGTGTG	686	UPRE-1
TERG_05150	tRNA wybutosine-synthesizing protein 2 (*T. equinum*)	CACTCTG (rc)	61	UPRE-1
TERG_05154	protein-vacuolar targeting protein Atg18 (*T. tonsurans*)	CACTCTG (rc)	351	UPRE-1
TERG_05154	protein-vacuolar targeting protein Atg18 (*T. tonsurans*)	CACCCTG (rc)	567	UPRE-1
TERG_05155	Leucine carboxyl methyltransferase family (*A. benhamiae*)	CAGGGTG	745	UPRE-1
TERG_05155	Leucine carboxyl methyltransferase family (*A. benhamiae*)	CAGAGTG	961	UPRE-1
TERG_05178	nuclear and cytoplasmic polyadenylated RNA-binding protein pub1 (*T. equinum*)	CAGTGTG	652	UPRE-1
TERG_05180	glucosamine 6-phosphate synthetase (*T. equinum*)	CACTCTG (rc)	283	UPRE-1
TERG_05182	msp1 (*T. equinum*)	CAGAGTG	312	UPRE-1
TERG_05185	RING finger ubiquitin ligase (*T. tonsurans*)	CACACTG (rc)	156	UPRE-1
TERG_05191	hypothetical protein	CAGAGTG	202	UPRE-1
TERG_05195	radical SAM domain-containing protein (*T. tonsurans*)	CACTCTG (rc)	252	UPRE-1
TERG_05225	nuclear localization protein (*T. equinum*)	CAGAGTG	42	UPRE-1
TERG_05236	60S ribosomal protein L35	CACACTG (rc)	746	UPRE-1
TERG_05241	hypothetical protein	CACGCTG (rc)	418	UPRE-1
TERG_05253	chromodomain helicase (*T. tonsurans*)	CACGCTG (rc)	587	UPRE-1
TERG_05270	C6 and C2H2 transcription factor RegA-like, putative (*A. benhamiae*)	CAGAGTG	346	UPRE-1
TERG_05278	microtubule associated protein (*T. tonsurans*)	CAGCGTG	906	UPRE-1
TERG_05285	translation initiation factor 4B (*T. equinum*)	CACGCTG (rc)	711	UPRE-1
TERG_05303	phospholipase PldA, putative (*T. verrucosum*)	CACTCTG (rc)	40	UPRE-1
TERG_05303	phospholipase PldA, putative (*T. verrucosum*)	CACGCTG (rc)	949	UPRE-1
TERG_05304	cyanate hydratase	CAGAGTG	659	UPRE-1
TERG_05305	glutathione-dependent formaldehyde-activating enzyme (*T. tonsurans*)	CACTCTG (rc)	739	UPRE-1

TERG_05307	hypothetical protein	CAGTGTG	392	UPRE-1
TERG_05313	PEK protein kinase	CACTCTG (rc)	589	UPRE-1
TERG_05318	polyphosphoinositide phosphatase (*T. tonsurans*)	CAGAGTG	502	UPRE-1
TERG_05323	hypothetical protein	CACACTG (rc)	139	UPRE-1
TERG_05327	DUF1237 domain-containing protein (*T. equinum*)	CAGTGTG	71	UPRE-1
TERG_05348	nuclear protein SDK3, putative (*A. benhamiae*)	CACCCTG (rc)	145	UPRE-1
TERG_05349	oxidoreductase, 2OG-Fe(II) oxygenase family, putative (*A. benhamiae*)	CACCCTG (rc)	480	UPRE-1
TERG_05352	benzoate 4-monooxygenase cytochrome P450 (*T. equinum*)	CACACTG (rc)	627	UPRE-1
TERG_05357	succinate:fumarate antiporter (*T. tonsurans*)	CACGCTG (rc)	246	UPRE-1
TERG_05358	CAF1 family ribonuclease, putative (*A. benhamiae*)	CACCCTG (rc)	673	UPRE-1
TERG_05360	50S ribosomal subunit L30 (*T. equinum*)	CAGAGTG	311	UPRE-1
TERG_05363	hypothetical protein	CAGTGTG	176	UPRE-1
TERG_05381	sodium/hydrogen antiporter	CACCCTG (rc)	564	UPRE-1
TERG_05389	septum-promoting GTP-binding protein 1	CAGGGTG	18	UPRE-1
TERG_05391	MFS glucose transporter, putative (*T. verrucosum*)	CAGTGTG	539	UPRE-1
TERG_05395	serine/threonine protein kinase (*T. tonsurans*)	CACGCTG (rc)	153	UPRE-1
TERG_05397	beta-lactamase (*T. equinum*)	CACTCTG (rc)	120	UPRE-1
TERG_05407	phosphotransferase enzyme family protein (*T. equinum*)	CACGCTG (rc)	269	UPRE-1
TERG_05408	ThiJ/PfpI family protein (*T. tonsurans*)	CAGCGTG	741	UPRE-1
TERG_05415	TRI7-like toxin biosynthesis protein, putative (*A. benhamiae*)	CAGAGTG	174	UPRE-1
TERG_05416	hypothetical protein	CAGCGTG	43	UPRE-1
TERG_05416	hypothetical protein	CAGGGTG	239	UPRE-1
TERG_05421	alpha-galactosidase A (*M. canis*)	CACACTG (rc)	162	UPRE-1
TERG_05422	hypothetical protein	CAGTGTG	818	UPRE-1
TERG_05424	hypothetical protein	CAGTGTG	191	UPRE-1
TERG_05430	hypothetical protein	CACTCTG (rc)	406	UPRE-1
TERG_05430	hypothetical protein	CACCCTG (rc)	857	UPRE-1
TERG_05431	sterol 24-C-methyltransferase (*T. tonsurans*)	CACACTG (rc)	966	UPRE-1
TERG_05432	pre-mRNA-splicing factor slt11	CAGTGTG	579	UPRE-1
TERG_05434	ribosome associated DnaJ chaperone Zuotin (*T. equinum*)	CAGCGTG	521	UPRE-1
TERG_05443	hypothetical protein	CACTCTG (rc)	604	UPRE-1
TERG_05446	sarcosine oxidase (*T. equinum*)	CACACTG (rc)	330	UPRE-1
TERG_05447	lysophospholipase NTE1	CAGTGTG	291	UPRE-1
TERG_05448	hypothetical protein	CACGCTG (rc)	423	UPRE-1

TERG_05449	viral A-type inclusion protein repeat protein (*T. tonsurans*)	CAGCGTG	486	UPRE-1
TERG_05458	MGMT family protein (*T. tonsurans*)	CACCCTG (rc)	924	UPRE-1
TERG_05459	hypothetical protein	CAGGGTG	449	UPRE-1
TERG_05462	CMGC/SRPK protein kinase	CACTCTG (rc)	129	UPRE-1
TERG_05463	phosphoric ester hydrolase (*T. equinum*)	CAGGGTG	316	UPRE-1
TERG_05467	hypothetical protein	CACTCTG (rc)	480	UPRE-1
TERG_05468	serine/threonine protein kinase	CAGAGTG	438	UPRE-1
TERG_05472	hypothetical protein	CACACTG (rc)	601	UPRE-1
TERG_05474	profilin-A (*T. equinum*)	CAGTGTG	89	UPRE-1
TERG_05479	bZIP transcription factor, putative (*T. verrucosum*)	CACCCTG (rc)	402	UPRE-1
TERG_05488	hypothetical protein	CACTCTG (rc)	68	UPRE-1
TERG_05492	hypothetical protein	CAGAGTG	167	UPRE-1
TERG_05493	hypothetical protein	CAGAGTG	591	UPRE-1
TERG_05511	AT DNA binding protein (*T. equinum*)	CACGCTG (rc)	850	UPRE-1
TERG_05518	oxidoreductase, short chain dehydrogenase/reductase family (*A. benhamiae*)	CACACTG (rc)	83	UPRE-1
TERG_05526	MFS multidrug transporter (*T. tonsurans*)	CACTCTG (rc)	859	UPRE-1
TERG_05528	hypothetical protein	CACGCTG (rc)	510	UPRE-1
TERG_05529	telomere length regulator protein (Rif1), putative (*A. benhamiae*)	CACCCTG (rc)	65	UPRE-1
TERG_05537	MFS multidrug transporter (*T. equinum*)	CACGCTG (rc)	121	UPRE-1
TERG_05537	MFS multidrug transporter (*T. equinum*)	CACCCTG (rc)	578	UPRE-1
TERG_05538	ABC a-pheromone efflux pump AtrD (*T. verrucosum*)	CAGCGTG	108	UPRE-1
TERG_05546	palmitoyltransferase pfa4 (*M. gypseum*)	CACTCTG (rc)	235	UPRE-1
TERG_05560	histone acetyltransferase GCN5 (*T. tonsurans*)	CAGTGTG	427	UPRE-1
TERG_05561	histone acetyltransferase GCN5 (*T. tonsurans*)	CACCCTG (rc)	308	UPRE-1
TERG_05562	2-hydroxychromene-2-carboxylate isomerase, putative (*A. benhamiae*)	CACGCTG (rc)	715	UPRE-1
TERG_05563	hypothetical protein	CAGCGTG	523	UPRE-1
TERG_05565	hypothetical protein	CACGCTG (rc)	55	UPRE-1
TERG_05583	polyketide synthase, putative (*A. benhamiae*)	CACTCTG (rc)	417	UPRE-1
TERG_05583	polyketide synthase, putative (*A. benhamiae*)	CACCCTG (rc)	933	UPRE-1
TERG_05620	RING finger protein (*T. tonsurans*)	CACCCTG (rc)	894	UPRE-1
TERG_05645	vacuolar protein sorting protein Vps36 (*T. tonsurans*)	CACTCTG (rc)	925	UPRE-1
TERG_05646	GANP domain-containing protein (*M. canis*)	CAGAGTG	856	UPRE-1
TERG_05656	thymidylate synthase	CACACTG (rc)	574	UPRE-1
TERG_05659	hypothetical protein	CACGCTG (rc)	412	UPRE-1

TERG_05665	protein kinase, putative (*A. benhamiae*)	CAGCGTG	819	UPRE-1
TERG_05666	YggS family pyridoxal phosphate enzyme	CAGCGTG	510	UPRE-1
TERG_05671	THO complex subunit 1 (*T. equinum*)	CACACTG (rc)	923	UPRE-1
TERG_05672	intron-binding protein aquarius (*T. equinum*)	CAGTGTG	420	UPRE-1
TERG_05673	PaaI-thioesterase (*T. equinum*)	CACCCTG (rc)	806	UPRE-1
TERG_05679	3-hydroxyacyl-CoA dehydrogenase, putative (*A. benhamiae*)	CAGGGTG	625	UPRE-1
TERG_05680	IBR domain containing protein (*T. equinum*)	CACACTG (rc)	195	UPRE-1
TERG_05688	GTP binding protein (*T. equinum*)	CACCCTG (rc)	602	UPRE-1
TERG_05692	HSF-type DNA-binding domain-containing protein (*M. canis*)	CAGCGTG	721	UPRE-1
TERG_05697	short chain dehydrogenase/reductase (*T. tonsurans*)	CACCCTG (rc)	135	UPRE-1
TERG_05702	Atypical/ABC1/ABC1-C protein kinase	CACACTG (rc)	506	UPRE-1
TERG_05705	a-pheromone receptor PreA (*A. benhamiae*)	CACGCTG (rc)	496	UPRE-1
TERG_05707	phosphatase family protein (*T. verrucosum*)	CACGCTG (rc)	118	UPRE-1
TERG_05734	ubiquitin fusion degradation protein (*T. tonsurans*)	CACCCTG (rc)	327	UPRE-1
TERG_05741	DNA-directed RNA polymerase II (*T. tonsurans*)	CAGTGTG	178	UPRE-1
TERG_05744	GTP-binding protein EsdC (*T. equinum*)	CAGTGTG	781	UPRE-1
TERG_05752	serine/threonine-protein phosphatase PP1	CAGCGTG	217	UPRE-1
TERG_05754	hypothetical protein	CACTCTG (rc)	249	UPRE-1
TERG_05754	hypothetical protein	CACCCTG (rc)	838	UPRE-1
TERG_05761	hypothetical protein	CAGCGTG	191	UPRE-1
TERG_05766	hypothetical protein	CAGAGTG	560	UPRE-1
TERG_05774	general stress response phosphoprotein phosphatase Psr1/2, putative (*A. benhamiae*)	CAGAGTG	29	UPRE-1
TERG_05780	hypothetical protein	CACACTG (rc)	147	UPRE-1
TERG_05783	Sin3 complex subunit (Stb2), putative (*T. verrucosum*)	CACCCTG (rc)	888	UPRE-1
TERG_05784	macrophage erythroblast attacher (*M. gypseum*)	CAGGGTG	291	UPRE-1
TERG_05788	60S ribosomal protein L18A (*T. tonsurans*)	CAGAGTG	294	UPRE-1
TERG_05788	60S ribosomal protein L18A (*T. tonsurans*)	CAGGGTG	381	UPRE-1
TERG_05796	transcriptional regulator Ngg1 (*T. equinum*)	CACGCTG (rc)	833	UPRE-1
TERG_05805	multidrug resistance protein fnx1 (*T. equinum*)	CACGCTG (rc)	711	UPRE-1
TERG_05811	40S ribosomal protein S8 (*T. tonsurans*)	CACCCTG (rc)	39	UPRE-1
TERG_05813	1-phosphatidylinositol-3-phosphate 5-kinase (*T. equinum*)	CACACTG (rc)	7	UPRE-1
TERG_05813	1-phosphatidylinositol-3-phosphate 5-kinase (*T. equinum*)	CACTCTG (rc)	343	UPRE-1
TERG_05830	hypothetical protein	CAGGGTG	682	UPRE-1
TERG_05831	hypothetical protein	CAGGGTG	123	UPRE-1

TERG_05837	plasma membrane ammonium transporter (Ato3), putative (*A. benhamiae*)	CAGAGTG	98	UPRE-1
TERG_05844	U3 small nucleolar RNA-associated protein 4 (*T. equinum*)	CACTCTG (rc)	184	UPRE-1
TERG_05859	hypothetical protein	CACCCTG (rc)	829	UPRE-1
TERG_05860	nicotinate phosphoribosyltransferase	CAGGGTG	722	UPRE-1
TERG_05872	thiosulfate sulfurtransferase, putative (*T. verrucosum*)	CAGGGTG	583	UPRE-1
TERG_05873	hypothetical protein	CAGAGTG	902	UPRE-1
TERG_05875	Ser/Thr protein phosphatase (*T. equinum*)	CAGGGTG	919	UPRE-1
TERG_05886	hypothetical protein	CACACTG (rc)	518	UPRE-1
TERG_05896	hypothetical protein	CAGTGTG	6	UPRE-1
TERG_05897	CoA-binding protein (*T. equinum*)	CACGCTG (rc)	261	UPRE-1
TERG_05922	acyl-CoA desaturase	CAGAGTG	775	UPRE-1
TERG_05924	transcription factor TFIIIB complex subunit brf1 (*M. gypseum*)	CAGCGTG	338	UPRE-1
TERG_05951	alpha/beta hydrolase (*T. tonsurans*)	CAGGGTG	50	UPRE-1
TERG_05963	WD repeat protein (*T. tonsurans*)	CAGTGTG	717	UPRE-1
TERG_05973	WD repeat protein (*T. tonsurans*)	CAGGGTG	396	UPRE-1
TERG_05992	translational initiation factor 2 beta (*T. tonsurans*)	CAGAGTG	104	UPRE-1
TERG_05993	DUF124 domain-containing protein (*T. equinum*)	CACTCTG (rc)	803	UPRE-1
TERG_05995	conserved serine-rich protein (*A. benhamiae*)	CAGCGTG	848	UPRE-1
TERG_05996	AT DNA binding protein (*T. equinum*)	CACCCTG (rc)	870	UPRE-1
TERG_06002	NIMA-interacting protein TinC (*T. equinum*)	CACCCTG (rc)	164	UPRE-1
TERG_06008	FAD-linked sulfhydryl oxidase ALR (*T. equinum*)	CACCCTG (rc)	313	UPRE-1
TERG_06021	proteasome regulatory particle subunit (*T. equinum*)	CACGCTG (rc)	195	UPRE-1
TERG_06022	hypothetical protein	CAGTGTG	48	UPRE-1
TERG_06029	UDP-N-acetylglucosamine pyrophosphorylase (*T. equinum*)	CAGCGTG	138	UPRE-1
TERG_06032	ribosomal small subunit assembly protein, putative (*A. benhamiae*)	CAGAGTG	911	UPRE-1
TERG_06036	hypothetical protein	CAGAGTG	202	UPRE-1
TERG_06036	hypothetical protein	CAGAGTG	561	UPRE-1
TERG_06037	OefA protein (*M. gypseum*)	CACTCTG (rc)	523	UPRE-1
TERG_06051	4-dimethylallyltryptophan methyltransferase (*T. tonsurans*)	CACCCTG (rc)	452	UPRE-1
TERG_06054	hypothetical protein	CACTCTG (rc)	17	UPRE-1
TERG_06055	NmrA-like family protein (*A. benhamiae*)	CAGAGTG	190	UPRE-1
TERG_06058	NADH:ubiquinone oxidoreductase 20.1kD subunit (*T. tonsurans*)	CACTCTG (rc)	190	UPRE-1

TERG_06065	conserved glycine-rich protein (*T. verrucosum*)	CAGGGTG	607	UPRE-1
TERG_06066	Mss51p (*M. gypseum*)	CACACTG (rc)	249	UPRE-1
TERG_06066	Mss51p (*M. gypseum*)	CACGCTG (rc)	427	UPRE-1
TERG_06081	hypothetical protein	CAGAGTG	281	UPRE-1
TERG_06087	mRNA export factor mex67 (*T. equinum*)	CACTCTG (rc)	451	UPRE-1
TERG_06087	mRNA export factor mex67 (*T. equinum*)	CACTCTG (rc)	687	UPRE-1
TERG_06088	PH domain-containing protein (*T. equinum*)	CAGAGTG	46	UPRE-1
TERG_06091	DNA-directed RNA polymerase I (*T. tonsurans*)	CAGAGTG	94	UPRE-1
TERG_06093	MFS monocarboxylate transporter, putative (*T. verrucosum*)	CAGGGTG	607	UPRE-1
TERG_06116	indoleamine 2,3-dioxygenase (*T. equinum*)	CAGGGTG	263	UPRE-1
TERG_06121	wd and tetratricopeptide repeat protein (*T. tonsurans*)	CAGGGTG	655	UPRE-1
TERG_06125	cell division control protein 16 (*T. equinum*)	CACACTG (rc)	487	UPRE-1
TERG_06131	hypothetical protein	CAGGGTG	919	UPRE-1
TERG_06148	protein vts1	CACGCTG (rc)	338	UPRE-1
TERG_06157	ATP synthase F1 epsilon subunit (*T. equinum*)	CACTCTG (rc)	309	UPRE-1
TERG_06166	hypothetical protein	CACGCTG (rc)	626	UPRE-1
TERG_06196	hypothetical protein	CACTCTG (rc)	270	UPRE-1
TERG_06197	cytochrome c oxidase assembly protein (*T. tonsurans*)	CACTCTG (rc)	412	UPRE-1
TERG_06199	lipase (*T. tonsurans*)	CAGAGTG	39	UPRE-1
TERG_06209	UTR2 protein (*M. gypseum*)	CAGTGTG	786	UPRE-1
TERG_06212	vacuolar ABC heavy metal transporter (Hmt1) (*T. tonsurans*)	CACGCTG (rc)	266	UPRE-1
TERG_06220	hypothetical protein	CAGTGTG	224	UPRE-1
TERG_06222	MYB DNA-binding domain-containing protein (*T. equinum*)	CAGGGTG	602	UPRE-1
TERG_06225	hypothetical protein	CACTCTG (rc)	495	UPRE-1
TERG_06235	hypothetical protein	CAGGGTG	857	UPRE-1
TERG_06237	dual specificity phosphatase catalytic domain-containing protein (*T. equinum*)	CACTCTG (rc)	744	UPRE-1
TERG_06242	glucanase, putative (*T. verrucosum*)	CACGCTG (rc)	692	UPRE-1
TERG_06247	DUF821 domain-containing protein (*T. equinum*)	CAGGGTG	728	UPRE-1
TERG_06249	hypothetical protein	CACTCTG (rc)	498	UPRE-1
TERG_06249	hypothetical protein	CACTCTG (rc)	531	UPRE-1
TERG_06254	pre-mRNA splicing factor ATP-dependent RNA helicase prp16 (*T. equinum*)	CAGAGTG	375	UPRE-1
TERG_06264	steroid monooxygenase, putative (*T. verrucosum*)	CAGCGTG	314	UPRE-1
TERG_06267	hypothetical protein	CAGCGTG	241	UPRE-1
TERG_06269	hypothetical protein	CACCCTG (rc)	447	UPRE-1

TERG_06270	hypothetical protein	CACTCTG (rc)	134	UPRE-1
TERG_06283	cyclohexanone 1,2-monooxygenase (*T. tonsurans*)	CACGCTG (rc)	776	UPRE-1
TERG_06287	cellular morphogenesis protein (*T. equinum*)	CACTCTG (rc)	698	UPRE-1
TERG_06288	FACT complex subunit pob3	CAGAGTG	699	UPRE-1
TERG_06325	autophagy protein 16 (*T. equinum*)	CACTCTG (rc)	31	UPRE-1
TERG_06327	integral membrane protein (*T. equinum*)	CAGAGTG	522	UPRE-1
TERG_06337	hypothetical protein	CAGAGTG	923	UPRE-1
TERG_06339	hypothetical protein	CACCCTG (rc)	279	UPRE-1
TERG_06340	AGC/PKC protein kinase	CAGGGTG	988	UPRE-1
TERG_06342	beta-tubulin cofactor d (*T. tonsurans*)	CAGTGTG	353	UPRE-1
TERG_06351	WD repeat protein (*T. tonsurans*)	CAGGGTG	26	UPRE-1
TERG_06377	metalloproteinase, putative (*A. benhamiae*)	CACGCTG (rc)	414	UPRE-1
TERG_06379	transcription factor RfeF (*T. tonsurans*)	CAGAGTG	874	UPRE-1
TERG_06399	ATP-dependent permease MDL2 (*T. equinum*)	CAGTGTG	10	UPRE-1
TERG_06420	RNA polymerase II Elongator subunit (*T. tonsurans*)	CACACTG (rc)	661	UPRE-1
TERG_06421	hypothetical protein	CAGTGTG	482	UPRE-1
TERG_06429	sterol delta 5,6-desaturase, putative (*T. verrucosum*)	CACGCTG (rc)	582	UPRE-1
TERG_06457	hypothetical protein	CACTCTG (rc)	152	UPRE-1
TERG_06459	SWIRM domain-containing protein (*T. tonsurans*)	CAGCGTG	591	UPRE-1
TERG_06465	O-mannosyl transferase pmtA-Aspergillus niger (*T. equinum*)	CAGTGTG	91	UPRE-1
TERG_06470	hypothetical protein	CACACTG (rc)	610	UPRE-1
TERG_06474	hypothetical protein	CAGCGTG	520	UPRE-1
TERG_06478	peroxisomal membrane protein pex16 (*T. equinum*)	CAGAGTG	228	UPRE-1
TERG_06481	cell polarity protein (*T. tonsurans*)	CACTCTG (rc)	530	UPRE-1
TERG_06489	hypothetical protein	CAGGGTG	65	UPRE-1
TERG_06493	mRNA cleavage factor complex component Pcf11 (*T. equinum*)	CACCCTG (rc)	268	UPRE-1
TERG_06493	mRNA cleavage factor complex component Pcf11 (*T. equinum*)	CACTCTG (rc)	481	UPRE-1
TERG_06494	hypothetical protein	CAGGGTG	38	UPRE-1
TERG_06494	hypothetical protein	CAGTGTG	968	UPRE-1
TERG_06524	hypothetical protein	CACGCTG (rc)	625	UPRE-1
TERG_06525	plasma membrane antiporter, putative (*T. verrucosum*)	CAGCGTG	620	UPRE-1
TERG_06526	NAD binding Rossmann fold oxidoreductase (*T. tonsurans*)	CACACTG (rc)	593	UPRE-1
TERG_06542	cyclin (*T. tonsurans*)	CAGTGTG	353	UPRE-1
TERG_06542	cyclin (*T. tonsurans*)	CAGAGTG	593	UPRE-1

TERG_06543	phosphatidate cytidylyltransferase (*T. tonsurans*)	CACTCTG (rc)	472	UPRE-1
TERG_06544	DNA repair protein Rad26 (*T. equinum*)	CAGAGTG	742	UPRE-1
TERG_06548	hypothetical protein	CAGCGTG	217	UPRE-1
TERG_06552	aspartic-type endopeptidase (OpsB), putative (*T. verrucosum*)	CACGCTG (rc)	639	UPRE-1
TERG_06560	pyruvate dehydrogenase (acetyl-transferring) E1 component, alpha subunit	CAGGGTG	518	UPRE-1
TERG_06561	sex determining protein (*M. canis*)	CACTCTG (rc)	458	UPRE-1
TERG_06563	30S ribosomal subunit S4 (*T. tonsurans*)	CACTCTG (rc)	642	UPRE-1
TERG_06563	30S ribosomal subunit S4 (*T. tonsurans*)	CACGCTG (rc)	917	UPRE-1
TERG_06564	alpha/beta hydrolase, putative (*T. verrucosum*)	CAGCGTG	312	UPRE-1
TERG_06564	alpha/beta hydrolase, putative (*T. verrucosum*)	CAGAGTG	587	UPRE-1
TERG_06576	hypothetical protein	CAGAGTG	794	UPRE-1
TERG_06592	E3 ubiquitin-protein ligase bre1 (*T. tonsurans*)	CACTCTG (rc)	792	UPRE-1
TERG_06593	transcription factor TFIIIB component (*T. equinum*)	CAGAGTG	533	UPRE-1
TERG_06615	hypothetical protein	CACGCTG (rc)	34	UPRE-1
TERG_06624	HATPase-c domain-containing protein (*T. equinum*)	CAGAGTG	967	UPRE-1
TERG_06631	vacuolar protein sorting-associated protein 55 (*T. tonsurans*)	CAGCGTG	341	UPRE-1
TERG_06634	hsp70-like protein (*T. equinum*)	CACGCTG (rc)	251	UPRE-1
TERG_06638	endochitinase (*T. equinum*)	CACACTG (rc)	167	UPRE-1
TERG_06645	alpha/beta hydrolase (*T. equinum*)	CACACTG (rc)	378	UPRE-1
TERG_06646	arrestin (*T. equinum*)	CAGTGTG	57	UPRE-1
TERG_06647	alpha/beta superfamily hydrolase, putative (*A. benhamiae*)	CACGCTG (rc)	369	UPRE-1
TERG_06652	Amt4 (*T. equinum*)	CACCCTG (rc)	504	UPRE-1
TERG_06653	conserved serine-rich protein (*T. verrucosum*)	CACACTG (rc)	899	UPRE-1
TERG_06665	serine carboxypeptidase (*T. tonsurans*)	CACGCTG (rc)	422	UPRE-1
TERG_06665	serine carboxypeptidase (*T. tonsurans*)	CACCCTG (rc)	430	UPRE-1
TERG_06673	pachytene checkpoint component Pch2 (*T. tonsurans*)	CAGGGTG	348	UPRE-1
TERG_06680	DUF221 domain-containing protein (*T. equinum*)	CACACTG (rc)	87	UPRE-1
TERG_06684	molybdenum cofactor biosynthetic protein (*T. equinum*)	CAGAGTG	417	UPRE-1
TERG_06686	hypothetical protein	CACGCTG (rc)	735	UPRE-1
TERG_06689	succinate dehydrogenase [ubiquinone] iron-sulfur subunit, mitochondrial	CACGCTG (rc)	731	UPRE-1
TERG_06695	hypothetical protein	CACGCTG (rc)	675	UPRE-1
TERG_06698	hypothetical protein	CAGTGTG	21	UPRE-1
TERG_06720	lipase/esterase, putative (*A. benhamiae*)	CAGAGTG	541	UPRE-1
TERG_06726	O-methylsterigmatocystin oxidoreductase, putative (*A. benhamiae*)	CAGAGTG	675	UPRE-1

TERG_06732	peroxisomal membrane protein receptor Pex19, putative (*A. benhamiae*)	CACGCTG (rc)	747	UPRE-1
TERG_06748	hypothetical protein	CAGCGTG	556	UPRE-1
TERG_06759	C2H2 transcription factor (Seb1), putative (*A. benhamiae*)	CAGGGTG	135	UPRE-1
TERG_06765	ULK/ULK protein kinase	CAGTGTG	6	UPRE-1
TERG_06771	FAD binding domain protein (*A. benhamiae*)	CAGAGTG	377	UPRE-1
TERG_06777	hypothetical protein	CACCCTG (rc)	267	UPRE-1
TERG_06780	hypothetical protein	CACTCTG (rc)	724	UPRE-1
TERG_06785	cytochrome P450 monooxygenase (*T. equinum*)	CAGGGTG	91	UPRE-1
TERG_06787	hypothetical protein	CACACTG (rc)	38	UPRE-1
TERG_06789	CDK-activating kinase assembly factor MAT1	CAGAGTG	307	UPRE-1
TERG_06790	hypothetical protein	CACTCTG (rc)	212	UPRE-1
TERG_06790	hypothetical protein	CACACTG (rc)	369	UPRE-1
TERG_06790	hypothetical protein	CACGCTG (rc)	630	UPRE-1
TERG_06791	short chain dehydrogenase/reductase, putative (*T. verrucosum*)	CAGGGTG	742	UPRE-1
TERG_06793	PEX11 domain protein (*A. benhamiae*)	CACACTG (rc)	768	UPRE-1
TERG_06794	minor allergen Alt a 7	CAGTGTG	492	UPRE-1
TERG_06795	nuclear protein localization protein 4	CAGAGTG	763	UPRE-1
TERG_06806	hypothetical protein	CACCCTG (rc)	33	UPRE-1
TERG_06806	hypothetical protein	CACTCTG (rc)	91	UPRE-1
TERG_06806	hypothetical protein	CACCCTG (rc)	881	UPRE-1
TERG_06808	RNA-binding protein 8A (*T. tonsurans*)	CAGTGTG	578	UPRE-1
TERG_06816	14-3-3 family protein (*T. tonsurans*)	CACCCTG (rc)	26	UPRE-1
TERG_06817	hypothetical protein	CAGGGTG	612	UPRE-1
TERG_06824	60S ribosomal protein L23	CACACTG (rc)	506	UPRE-1
TERG_06829	hypothetical protein	CACTCTG (rc)	346	UPRE-1
TERG_06831	F-box domain protein (*A. benhamiae*)	CAGGGTG	498	UPRE-1
TERG_06837	membrane transporter (*T. tonsurans*)	CACCCTG (rc)	157	UPRE-1
TERG_06849	SH3 domain-containing protein (*T. tonsurans*)	CACCCTG (rc)	416	UPRE-1
TERG_06850	monocarboxylate permease (*T. equinum*)	CAGGGTG	273	UPRE-1
TERG_06851	hypothetical protein	CACGCTG (rc)	828	UPRE-1
TERG_06865	hypothetical protein	CACGCTG (rc)	779	UPRE-1
TERG_06866	isochorismatase family hydrolase (*T. equinum*)	CAGCGTG	887	UPRE-1
TERG_06880	MFS transporter Fmp42, putative (*T. verrucosum*)	CACCCTG (rc)	810	UPRE-1
TERG_06882	hypothetical protein	CACCCTG (rc)	790	UPRE-1
TERG_06918	hypothetical protein	CACGCTG (rc)	871	UPRE-1

TERG_06919	hypothetical protein	CAGCGTG	768	UPRE-1
TERG_06920	hypothetical protein	CACCCTG (rc)	40	UPRE-1
TERG_06925	class V chitinase (*T. tonsurans*)	CAGTGTG	183	UPRE-1
TERG_06929	chitinase (*T. equinum*)	CACACTG (rc)	28	UPRE-1
TERG_06929	chitinase (*T. equinum*)	CACCCTG (rc)	552	UPRE-1
TERG_06932	serine/threonine protein kinase	CAGGGTG	145	UPRE-1
TERG_06932	serine/threonine protein kinase	CAGTGTG	669	UPRE-1
TERG_06937	hypothetical protein	CACCCTG (rc)	703	UPRE-1
TERG_06938	zinc metallopeptidase (*T. tonsurans*)	CAGGGTG	169	UPRE-1
TERG_06945	WD domain-containing protein (*T. equinum*)	CACCCTG (rc)	744	UPRE-1
TERG_06946	ARP2/3 complex 34 kDa subunit (*T. tonsurans*)	CAGTGTG	102	UPRE-1
TERG_06946	ARP2/3 complex 34 kDa subunit (*T. tonsurans*)	CAGGGTG	956	UPRE-1
TERG_06955	hypothetical protein	CACTCTG (rc)	475	UPRE-1
TERG_06956	RNA polymerase I-specific transcription initiation factor rrn3 (*M. gypseum*)	CAGAGTG	318	UPRE-1
TERG_06958	aspartate-tRNA(Asn) ligase	CAGCGTG	293	UPRE-1
TERG_06964	NTF2 domain-containing protein (*T. tonsurans*)	CACGCTG (rc)	895	UPRE-1
TERG_06967	rnapii degradation factor def1 (*T. tonsurans*)	CAGTGTG	351	UPRE-1
TERG_06969	CMGC/CDK/CDK5 protein kinase	CACCCTG (rc)	789	UPRE-1
TERG_06989	C6 finger domain protein, putative (*T. verrucosum*)	CACGCTG (rc)	551	UPRE-1
TERG_06989	C6 finger domain protein, putative (*T. verrucosum*)	CACTCTG (rc)	795	UPRE-1
TERG_07001	hydroxyisocaproate dehydrogenase (*T. tonsurans*)	CAGCGTG	214	UPRE-1
TERG_07005	PAB-dependent poly(A)-specific ribonuclease subunit PAN3 (*T. tonsurans*)	CAGGGTG	199	UPRE-1
TERG_07008	diaminopropionate ammonia-lyase (*T. equinum*)	CACCCTG (rc)	562	UPRE-1
TERG_07011	conserved fungal protein (*A. benhamiae*)	CAGCGTG	96	UPRE-1
TERG_07021	helix-loop-helix DNA-binding domain-containing protein (*T. tonsurans*)	CACACTG (rc)	370	UPRE-1
TERG_07038	glutamine-tRNA ligase	CACTCTG (rc)	528	UPRE-1
TERG_07045	hypothetical protein	CAGTGTG	175	UPRE-1
TERG_07047	cholestenol delta-isomerase, putative (*T. verrucosum*)	CAGCGTG	182	UPRE-1
TERG_07059	hypothetical protein	CAGGGTG	28	UPRE-1
TERG_07061	CMGC/SRPK protein kinase	CAGCGTG	128	UPRE-1
TERG_07079	C6 transcription factor, putative (*T. verrucosum*)	CAGTGTG	80	UPRE-1
TERG_07085	peptidase (*T. tonsurans*)	CACTCTG (rc)	167	UPRE-1
TERG_07089	GPI-anchored wall transfer protein 1 (*T. tonsurans*)	CACTCTG (rc)	569	UPRE-1
TERG_07094	hypothetical protein	CACCCTG (rc)	386	UPRE-1
TERG_07105	transcription factor TFIIA complex subunit Toa1, putative (*T. verrucosum*)	CACCCTG (rc)	243	UPRE-1

TERG_07108	WD-repeat containing protein slp1 (*T. tonsurans*)	CACTCTG (rc)	162	UPRE-1
TERG_07114	ubiquitin-protein ligase Sel1/Ubx2 (*T. equinum*)	CACTCTG (rc)	416	UPRE-1
TERG_07118	phosphotransferase enzyme family protein (*T. equinum*)	CACGCTG (rc)	189	UPRE-1
TERG_07132	hypothetical protein	CACTCTG (rc)	801	UPRE-1
TERG_07140	serine/threonine protein kinase (*T. tonsurans*)	CACCCTG (rc)	165	UPRE-1
TERG_07140	serine/threonine protein kinase (*T. tonsurans*)	CACTCTG (rc)	724	UPRE-1
TERG_07141	RING and UBP finger domain-containing protein (*T. tonsurans*)	CAGGGTG	520	UPRE-1
TERG_07146	3-demethylubiquinone-9 3-O-methyltransferase	CAGCGTG	983	UPRE-1
TERG_07154	iron donor protein CyaY	CAGCGTG	273	UPRE-1
TERG_07161	hypothetical protein	CAGAGTG	491	UPRE-1
TERG_07178	synaptic glycoprotein SC2 (*T. equinum*)	CACTCTG (rc)	689	UPRE-1
TERG_07188	xanthine dehydrogenase (*T. tonsurans*)	CAGTGTG	524	UPRE-1
TERG_07189	plasma membrane fusion protein PRM1 (*T. equinum*)	CACCCTG (rc)	77	UPRE-1
TERG_07190	hypothetical protein	CACACTG (rc)	217	UPRE-1
TERG_07202	acetyl-coenzyme A synthetase	CAGAGTG	206	UPRE-1
TERG_07207	Sit4-associated protein (*T. tonsurans*)	CACTCTG (rc)	694	UPRE-1
TERG_07222	carbonic anhydrase family protein (*T. verrucosum*)	CAGCGTG	228	UPRE-1
TERG_07228	MOSC domain-containing protein (*T. equinum*)	CACGCTG (rc)	62	UPRE-1
TERG_07229	vacuolar ATP synthase 116kDa subunit (*T. tonsurans*)	CAGAGTG	436	UPRE-1
TERG_07233	acriflavine sensitivity control protein acr-2 (*T. equinum*)	CACACTG (rc)	486	UPRE-1
TERG_07234	hypothetical protein	CAGTGTG	467	UPRE-1
TERG_07241	saccharopine dehydrogenase [NADP+, L-glutamate-forming]	CACGCTG (rc)	591	UPRE-1
TERG_07242	di-trans,poly-cis-decaprenylcistransferase	CACCCTG (rc)	122	UPRE-1
TERG_07242	di-trans,poly-cis-decaprenylcistransferase	CACGCTG (rc)	461	UPRE-1
TERG_07260	hypothetical protein	CAGAGTG	349	UPRE-1
TERG_07264	thermatolerance membrane protein Dlt1, putative (*A. benhamiae*)	CAGAGTG	528	UPRE-1
TERG_07265	sucrase/ferredoxin domain-containing protein (*T. equinum*)	CAGTGTG	302	UPRE-1
TERG_07269	mannose-1-phosphate guanyltransferase	CACTCTG (rc)	237	UPRE-1
TERG_07281	protein kinase subdomain-containing protein (*T. tonsurans*)	CACACTG (rc)	53	UPRE-1
TERG_07294	glyoxylate reductase (*T. tonsurans*)	CAGAGTG	661	UPRE-1
TERG_07308	iron-sulfur cluster assembly associated protein Nar1 (*T. tonsurans*)	CAGCGTG	931	UPRE-1
TERG_07312	metallothionein-I transcription activator (*T. equinum*)	CAGAGTG	543	UPRE-1

TERG_07313	anthranilate phosphoribosyltransferase	CACCCTG (rc)	164	UPRE-1
TERG_07313	anthranilate phosphoribosyltransferase	CACTCTG (rc)	728	UPRE-1
TERG_07314	hypothetical protein	CAGGGTG	70	UPRE-1
TERG_07324	ubiquitin-conjugating enzyme (*T. equinum*)	CAGAGTG	478	UPRE-1
TERG_07326	glutathione S-transferase PARB (*T. equinum*)	CACTCTG (rc)	584	UPRE-1
TERG_07327	pyridoxamine phosphate oxidase (*T. tonsurans*)	CACTCTG (rc)	651	UPRE-1
TERG_07327	pyridoxamine phosphate oxidase (*T. tonsurans*)	CACTCTG (rc)	911	UPRE-1
TERG_07330	chromatin regulatory protein sir2 (*T. tonsurans*)	CAGAGTG	202	UPRE-1
TERG_07335	G2/M-specific cyclin NimE (*T. tonsurans*)	CACGCTG (rc)	160	UPRE-1
TERG_07337	54S ribosomal protein RML2 (*T. equinum*)	CAGCGTG	264	UPRE-1
TERG_07338	C6 transcription factor Prf, putative (*A. benhamiae*)	CAGAGTG	17	UPRE-1
TERG_07343	HLH transcription factor (*T. tonsurans*)	CACGCTG (rc)	132	UPRE-1
TERG_07355	PAP2 domain protein (*A. benhamiae*)	CAGCGTG	413	UPRE-1
TERG_07361	hybrid PKS-NRPS enzyme, putative (*A. benhamiae*)	CACCCTG (rc)	531	UPRE-1
TERG_07363	hypothetical protein	CAGGGTG	380	UPRE-1
TERG_07364	oxidoreductase, zinc-binding dehydrogenase family superfamily (*T. verrucosum*)	CAGGGTG	811	UPRE-1
TERG_07379	leucine rich repeat protein (*T. equinum*)	CAGTGTG	605	UPRE-1
TERG_07382	importin beta-3 subunit (*T. tonsurans*)	CAGTGTG	706	UPRE-1
TERG_07406	alpha-mannosidase (*T. tonsurans*)	CAGAGTG	349	UPRE-1
TERG_07409	amino acid permease (*T. equinum*)	CACCCTG (rc)	99	UPRE-1
TERG_07417	hypothetical protein	CACTCTG (rc)	211	UPRE-1
TERG_07418	MFS multidrug transporter (*T. equinum*)	CACCCTG (rc)	509	UPRE-1
TERG_07425	JmjC domain-containing protein (*T. equinum*)	CAGAGTG	99	UPRE-1
TERG_07442	hypothetical protein	CAGCGTG	742	UPRE-1
TERG_07455	mitochondrial import protein mmp37 (*T. tonsurans*)	CAGTGTG	920	UPRE-1
TERG_07466	hypothetical protein	CAGGGTG	496	UPRE-1
TERG_07475	C6 transcription factor (*T. equinum*)	CACCCTG (rc)	87	UPRE-1
TERG_07477	copper-sulfate regulated protein 1 (*T. equinum*)	CACGCTG (rc)	935	UPRE-1
TERG_07482	ribonuclease H1, putative (*A. benhamiae*)	CACGCTG (rc)	286	UPRE-1
TERG_07489	hypothetical protein	CACCCTG (rc)	50	UPRE-1
TERG_07494	golgi apparatus membrane protein TVP23	CAGTGTG	43	UPRE-1
TERG_07495	acyl-CoA dehydrogenase family member 9 (*T. equinum*)	CACGCTG (rc)	113	UPRE-1
TERG_07495	acyl-CoA dehydrogenase family member 9 (*T. equinum*)	CACCCTG (rc)	232	UPRE-1
TERG_07503	phosphoribosylaminoimidazolecarboxamide formyltransferase/IMP cyclohydrolase	CACCCTG (rc)	352	UPRE-1
TERG_07510	hypothetical protein	CACTCTG (rc)	744	UPRE-1

TERG_07532	ubiquitination network signaling protein (*T. tonsurans*)	CAGGGTG	206	UPRE-1
TERG_07532	ubiquitination network signaling protein (*T. tonsurans*)	CAGGGTG	409	UPRE-1
TERG_07535	methyltransferase domain-containing protein (*T. tonsurans*)	CACCCTG (rc)	169	UPRE-1
TERG_07541	phosphoesterase (*T. tonsurans*)	CAGGGTG	341	UPRE-1
TERG_07553	C2H2 transcription factor Crz1, putative (*T. verrucosum*)	CACCCTG (rc)	606	UPRE-1
TERG_07553	C2H2 transcription factor Crz1, putative (*T. verrucosum*)	CACACTG (rc)	931	UPRE-1
TERG_07558	lipoyltransferase and lipoate-protein ligase (*T. equinum*)	CACTCTG (rc)	76	UPRE-1
TERG_07578	GTP-binding protein rhoA	CAGAGTG	191	UPRE-1
TERG_07582	hypothetical protein	CACCCTG (rc)	295	UPRE-1
TERG_07586	DNA methyltransferase 1-associated protein DMAP1 (*T. tonsurans*)	CAGCGTG	725	UPRE-1
TERG_07589	3-oxo-5-alpha-steroid 4-dehydrogenase family protein (*T. verrucosum*)	CACCCTG (rc)	758	UPRE-1
TERG_07597	SOK1 (*T. equinum*)	CACACTG (rc)	318	UPRE-1
TERG_07603	DNA helicase (*T. tonsurans*)	CAGCGTG	216	UPRE-1
TERG_07622	UBX domain-containing protein (*T. equinum*)	CAGGGTG	260	UPRE-1
TERG_07640	mannan polymerase II complex ANP1 subunit (*T. equinum*)	CACACTG (rc)	168	UPRE-1
TERG_07643	ubiquinol-cytochrome c reductase complex protein (*T. equinum*)	CACACTG (rc)	809	UPRE-1
TERG_07644	mitochondrial carrier protein (*T. tonsurans*)	CAGTGTG	549	UPRE-1
TERG_07647	hypothetical protein	CAGAGTG	372	UPRE-1
TERG_07650	membrane-tethered transcription factor (SPT23), putative (*A. benhamiae*)	CAGCGTG	757	UPRE-1
TERG_07652	Delta(12) fatty acid desaturase (*T. equinum*)	CAGGGTG	316	UPRE-1
TERG_07652	Delta(12) fatty acid desaturase (*T. equinum*)	CAGAGTG	885	UPRE-1
TERG_07664	rho GTPase activator Rga (*T. equinum*)	CACACTG (rc)	832	UPRE-1
TERG_07672	GABA permease (*T. equinum*)	CAGTGTG	191	UPRE-1
TERG_07683	hypothetical protein	CAGAGTG	272	UPRE-1
TERG_07692	hypothetical protein	CACACTG (rc)	160	UPRE-1
TERG_07692	hypothetical protein	CACACTG (rc)	204	UPRE-1
TERG_07693	mitochondrial carrier protein (*T. tonsurans*)	CAGTGTG	667	UPRE-1
TERG_07693	mitochondrial carrier protein (*T. tonsurans*)	CAGTGTG	711	UPRE-1
TERG_07698	CHL4 family chromosome segregation protein, putative (*T. verrucosum*)	CAGGGTG	475	UPRE-1
TERG_07706	hypothetical protein	CACCCTG (rc)	704	UPRE-1

TERG_07708	hypothetical protein	CACGCTG (rc)	567	UPRE-1
TERG_07708	hypothetical protein	CACGCTG (rc)	992	UPRE-1
TERG_07716	hypothetical protein	CACGCTG (rc)	351	UPRE-1
TERG_07719	hypothetical protein	CACTCTG (rc)	545	UPRE-1
TERG_07721	isopenicillin N-CoA epimerase (*T. equinum*)	CACTCTG (rc)	89	UPRE-1
TERG_07721	isopenicillin N-CoA epimerase (*T. equinum*)	CACCCTG (rc)	601	UPRE-1
TERG_07731	2Fe-2S iron-sulfur cluster binding domain-containing protein (*T. tonsurans*)	CACTCTG (rc)	42	UPRE-1
TERG_07731	2Fe-2S iron-sulfur cluster binding domain-containing protein (*T. tonsurans*)	CACGCTG (rc)	75	UPRE-1
TERG_07737	hypothetical protein	CACCCTG (rc)	98	UPRE-1
TERG_07737	hypothetical protein	CACTCTG (rc)	113	UPRE-1
TERG_07737	hypothetical protein	CACACTG (rc)	805	UPRE-1
TERG_07738	hypothetical protein	CAGTGTG	555	UPRE-1
TERG_07753	hypothetical protein	CAGCGTG	206	UPRE-1
TERG_07758	hypothetical protein	CAGGGTG	533	UPRE-1
TERG_07760	integral membrane protein (*T. verrucosum*)	CACTCTG (rc)	637	UPRE-1
TERG_07761	hypothetical protein	CAGAGTG	269	UPRE-1
TERG_07763	hypothetical protein	CACTCTG (rc)	94	UPRE-1
TERG_07764	protein transporter SEC61 subunit alpha	CAGAGTG	771	UPRE-1
TERG_07769	hypothetical protein	CACTCTG (rc)	367	UPRE-1
TERG_07773	tubulin alpha chain	CAGAGTG	551	UPRE-1
TERG_07775	hypothetical protein	CACCCTG (rc)	237	UPRE-1
TERG_07781	hypothetical protein	CAGAGTG	540	UPRE-1
TERG_07781	hypothetical protein	CAGGGTG	856	UPRE-1
TERG_07783	MFS peptide transporter, putative (*T. verrucosum*)	CACACTG (rc)	44	UPRE-1
TERG_07797	amino acid permease (*T. equinum*)	CACGCTG (rc)	499	UPRE-1
TERG_07803	HEAT repeat protein (*T. equinum*)	CACGCTG (rc)	706	UPRE-1
TERG_07803	HEAT repeat protein (*T. equinum*)	CACGCTG (rc)	730	UPRE-1
TERG_07804	PBSP domain-containing protein (*T. tonsurans*)	CAGCGTG	853	UPRE-1
TERG_07804	PBSP domain-containing protein (*T. tonsurans*)	CAGCGTG	877	UPRE-1
TERG_07806	54S ribosomal protein L3 (*T. equinum*)	CACACTG (rc)	555	UPRE-1
TERG_07823	ATP-dependent DNA helicase (*T. tonsurans*)	CAGTGTG	991	UPRE-1
TERG_07838	YjeF family domain-containing protein	CAGAGTG	154	UPRE-1
TERG_07839	ubiquitin homeostasis protein lub1 (*T. equinum*)	CACTCTG (rc)	658	UPRE-1
TERG_07839	ubiquitin homeostasis protein lub1 (*T. equinum*)	CACTCTG (rc)	666	UPRE-1
TERG_07847	hypothetical protein	CACCCTG (rc)	411	UPRE-1
TERG_07847	hypothetical protein	CACTCTG (rc)	874	UPRE-1

TERG_07856	hypothetical protein	CACGCTG (rc)	414	UPRE-1
TERG_07862	dynein light intermediate chain (*T. tonsurans*)	CACTCTG (rc)	40	UPRE-1
TERG_07862	dynein light intermediate chain (*T. tonsurans*)	CACTCTG (rc)	49	UPRE-1
TERG_07863	U2 snRNP component IST3 (*T. equinum*)	CAGCGTG	104	UPRE-1
TERG_07875	integral membrane protein (*T. equinum*)	CACTCTG (rc)	661	UPRE-1
TERG_07876	hypothetical protein	CACTCTG (rc)	814	UPRE-1
TERG_07891	DUF255 domain-containing protein (*T. equinum*)	CAGGGTG	987	UPRE-1
TERG_07899	hypothetical protein	CAGAGTG	927	UPRE-1
TERG_07902	STE/STE11/SSK protein kinase	CACTCTG (rc)	732	UPRE-1
TERG_07903	nuclear mRNA splicing factor-associated protein (*T. tonsurans*)	CAGAGTG	211	UPRE-1
TERG_07905	1-pyrroline-5-carboxylate dehydrogenase	CACACTG (rc)	228	UPRE-1
TERG_07907	hypothetical protein	CACTCTG (rc)	263	UPRE-1
TERG_07922	glycine cleavage system T protein	CACTCTG (rc)	176	UPRE-1
TERG_07932	vesicle-mediated transport protein Vid24, putative (*T. verrucosum*)	CACTCTG (rc)	848	UPRE-1
TERG_07940	histone-lysine n-methyltransferase (*T. equinum*)	CAGGGTG	842	UPRE-1
TERG_07943	hypothetical protein	CACCCTG (rc)	93	UPRE-1
TERG_07944	60S ribosomal protein L6 (*T. equinum*)	CAGGGTG	91	UPRE-1
TERG_07959	GPI-anchored cell wall beta-1,3-endoglucanase EglC (*A. benhamiae*)	CACTCTG (rc)	666	UPRE-1
TERG_07963	hypothetical protein	CACCCTG (rc)	240	UPRE-1
TERG_07969	acyl-CoA dehydrogenase (*T. tonsurans*)	CAGCGTG	282	UPRE-1
TERG_07971	macrophomate synthase, putative (*A. benhamiae*)	CAGGGTG	834	UPRE-1
TERG_07997	acetamidase (*T. tonsurans*)	CACTCTG (rc)	470	UPRE-1
TERG_07998	hypothetical protein	CACGCTG (rc)	780	UPRE-1
TERG_08001	nucleoside hydrolase, putative (*T. verrucosum*)	CACCCTG (rc)	721	UPRE-1
TERG_08002	hypothetical protein	CAGGGTG	618	UPRE-1
TERG_08002	hypothetical protein	CAGGGTG	977	UPRE-1
TERG_08011	metallocarboxypeptidase A-like protein	CAGGGTG	75	UPRE-1
TERG_08016	calcium transporter (*T. tonsurans*)	CACGCTG (rc)	432	UPRE-1
TERG_08031	PHD finger domain-containing protein (*T. equinum*)	CACACTG (rc)	14	UPRE-1
TERG_08038	polysaccharide deacetylase family protein (*T. equinum*)	CACGCTG (rc)	312	UPRE-1
TERG_08042	hypothetical protein	CAGGGTG	416	UPRE-1
TERG_08062	alpha-glucosidase AgdA, putative (*A. benhamiae*)	CACGCTG (rc)	484	UPRE-1
TERG_08064	choline kinase, putative (*A. benhamiae*)	CACCCTG (rc)	17	UPRE-1
TERG_08065	auxin Efflux Carrier superfamily protein (*T. equinum*)	CACCCTG (rc)	634	UPRE-1
TERG_08078	ubiquitin carboxyl-terminal hydrolase (*T. equinum*)	CAGTGTG	558	UPRE-1

TERG_08080	hypothetical protein	CAGGGTG	911	UPRE-1
TERG_08085	DUF803 domain membrane protein (*T. equinum*)	CACGCTG (rc)	772	UPRE-1
TERG_08090	plasma membrane iron permease (*T. tonsurans*)	CACCCTG (rc)	127	UPRE-1
TERG_08090	plasma membrane iron permease (*T. tonsurans*)	CACTCTG (rc)	958	UPRE-1
TERG_08091	conidial pigment biosynthesis oxidase Abr1/brown (*T. verrucosum*)	CAGGGTG	498	UPRE-1
TERG_08092	orotidine 5'-phosphate decarboxylase (*M. canis*)	CACCCTG (rc)	52	UPRE-1
TERG_08092	orotidine 5'-phosphate decarboxylase (*M. canis*)	CACTCTG (rc)	130	UPRE-1
TERG_08101	TAM domain methyltransferase (*T. equinum*)	CACACTG (rc)	367	UPRE-1
TERG_08103	anaphase promoting complex subunit 10 (APC10), putative (*A. benhamiae*)	CACCCTG (rc)	596	UPRE-1
TERG_08115	hypothetical protein	CACGCTG (rc)	720	UPRE-1
TERG_08115	hypothetical protein	CACTCTG (rc)	765	UPRE-1
TERG_08117	feruloyl esterase, putative (*A. benhamiae*)	CACCCTG (rc)	896	UPRE-1
TERG_08118	hypothetical protein	CACACTG (rc)	768	UPRE-1
TERG_08123	C6 finger domain protein, putative (*A. benhamiae*)	CAGAGTG	191	UPRE-1
TERG_08128	hypothetical protein	CACCCTG (rc)	184	UPRE-1
TERG_08141	bZIP transcription factor (LziP), putative (*A. benhamiae*)	CAGAGTG	901	UPRE-1
TERG_08150	Swr1p complex component (*T. equinum*)	CAGGGTG	375	UPRE-1
TERG_08161	hypothetical protein	CACCCTG (rc)	912	UPRE-1
TERG_08163	hypothetical protein	CAGGGTG	324	UPRE-1
TERG_08164	hypothetical protein	CAGAGTG	566	UPRE-1
TERG_08169	hypothetical protein	CACGCTG (rc)	65	UPRE-1
TERG_08171	C6 finger domain protein, putative (*A. benhamiae*)	CAGCGTG	172	UPRE-1
TERG_08172	hypothetical protein	CACTCTG (rc)	911	UPRE-1
TERG_08173	C6 finger domain protein, putative (*A. benhamiae*)	CAGAGTG	37	UPRE-1
TERG_08197	hypothetical protein	CACCCTG (rc)	816	UPRE-1
TERG_08199	hypothetical protein	CAGGGTG	6	UPRE-1
TERG_08200	hypothetical protein	CACTCTG (rc)	745	UPRE-1
TERG_08201	subtilisin-like protease 5	CAGCGTG	363	UPRE-1
TERG_08203	isopenicillin N synthase	CACTCTG (rc)	897	UPRE-1
TERG_08204	alpha-aminoadypil-cysteinyl-valine synthetase (*T. equinum*)	CAGAGTG	388	UPRE-1
TERG_08218	ADP-ribosylation factor family protein (*T. verrucosum*)	CACGCTG (rc)	17	UPRE-1
TERG_08222	hypothetical protein	CAGCGTG	890	UPRE-1
TERG_08228	sphinganine hydroxylase (*T. tonsurans*)	CAGTGTG	43	UPRE-1
TERG_08229	hypothetical protein	CACGCTG (rc)	137	UPRE-1
TERG_08235	long chain fatty alcohol oxidase (*T. tonsurans*)	CAGCGTG	889	UPRE-1

TERG_08236	hypothetical protein	CACACTG (rc)	133	UPRE-1
TERG_08237	DUF543 domain-containing protein (*T. equinum*)	CAGTGTG	279	UPRE-1
TERG_08240	methyltransferase, putative (*T. verrucosum*)	CACTCTG (rc)	93	UPRE-1
TERG_08250	cation diffusion facilitator family metal ion transporter, putative (*T. verrucosum*)	CACTCTG (rc)	992	UPRE-1
TERG_08264	L-xylulose reductase	CAGCGTG	720	UPRE-1
TERG_08271	RNA lariat debranching enzyme, putative (*A. benhamiae*)	CACACTG (rc)	945	UPRE-1
TERG_08272	hypothetical protein	CAGTGTG	594	UPRE-1
TERG_08282	hypothetical protein	CACTCTG (rc)	749	UPRE-1
TERG_08283	acetamidase (*T. tonsurans*)	CAGAGTG	932	UPRE-1
TERG_08284	DNA polymerase delta subunit 4, putative (*A. benhamiae*)	CAGAGTG	935	UPRE-1
TERG_08288	ATP-citrate synthase subunit 1	CAGAGTG	638	UPRE-1
TERG_08291	nuclear pore complex subunit (*T. equinum*)	CAGGGTG	510	UPRE-1
TERG_08310	XAP5 domain-containing protein (*T. equinum*)	CACTCTG (rc)	315	UPRE-1
TERG_08318	HAD superfamily hydrolase (*T. tonsurans*)	CACCCTG (rc)	675	UPRE-1
TERG_08319	CMGC/SRPK protein kinase	CACCCTG (rc)	59	UPRE-1
TERG_08323	serine/threonine-protein phosphatase PP2A catalytic subunit	CACTCTG (rc)	478	UPRE-1
TERG_08324	hypothetical protein	CACTCTG (rc)	308	UPRE-1
TERG_08327	Ser/Thr protein phosphatase (*T. tonsurans*)	CACTCTG (rc)	163	UPRE-1
TERG_08328	basic proline-rich protein (*M. canis*)	CAGAGTG	807	UPRE-1
TERG_08329	flavin containing amine oxidase (*T. tonsurans*)	CACACTG (rc)	649	UPRE-1
TERG_08333	1-pyrroline-5-carboxylate dehydrogenase	CAGAGTG	509	UPRE-1
TERG_08333	1-pyrroline-5-carboxylate dehydrogenase	CAGAGTG	666	UPRE-1
TERG_08343	hypothetical protein	CACTCTG (rc)	240	UPRE-1
TERG_08344	hypothetical protein	CAGCGTG	336	UPRE-1
TERG_08348	terpene synthase family protein (*A. benhamiae*)	CACACTG (rc)	105	UPRE-1
TERG_08348	terpene synthase family protein (*A. benhamiae*)	CACGCTG (rc)	579	UPRE-1
TERG_08351	hypothetical protein	CACCCTG (rc)	708	UPRE-1
TERG_08352	hypothetical protein	CAGGGTG	975	UPRE-1
TERG_08355	zinc alcohol dehydrogenase, putative (*T. verrucosum*)	CACACTG (rc)	326	UPRE-1
TERG_08355	zinc alcohol dehydrogenase, putative (*T. verrucosum*)	CACGCTG (rc)	959	UPRE-1
TERG_08356	oxidoreductase, 2OG-Fe(II) oxygenase family, putative (*A. benhamiae*)	CAGCGTG	934	UPRE-1
TERG_08377	copper transporter Ctr (*T. tonsurans*)	CACCCTG (rc)	219	UPRE-1
TERG_08381	NUDIX domain-containing protein (*T. equinum*)	CACCCTG (rc)	640	UPRE-1
TERG_08390	hypothetical protein	CAGAGTG	456	UPRE-1

TERG_08394	sugar transporter family protein (*T. verrucosum*)	CACCCTG (rc)	595	UPRE-1
TERG_08395	4-nitrophenylphosphatase (*T. tonsurans*)	CAGGGTG	359	UPRE-1
TERG_08402	protein transport membrane glycoprotein Sec20, putative (*A. benhamiae*)	CAGAGTG	402	UPRE-1
TERG_08403	inorganic pyrophosphatase	CACTCTG (rc)	67	UPRE-1
TERG_08412	TLC domain-containing protein (*T. tonsurans*)	CAGCGTG	362	UPRE-1
TERG_08418	mitochondrial inner membrane translocase subunit TIM44 (*T. tonsurans*)	CACTCTG (rc)	121	UPRE-1
TERG_08433	hypothetical protein	CACTCTG (rc)	116	UPRE-1
TERG_08435	3-oxoacyl-(acyl-carrier-protein) reductase (*M. canis*)	CACTCTG (rc)	119	UPRE-1
TERG_08436	hypothetical protein	CACACTG (rc)	124	UPRE-1
TERG_08466	hypothetical protein	CAGCGTG	153	UPRE-1
TERG_08469	signal peptidase I	CAGTGTG	754	UPRE-1
TERG_08471	50S ribosomal protein L4	CAGTGTG	735	UPRE-1
TERG_08497	mRNA turnover protein 4 (*T. equinum*)	CACTCTG (rc)	261	UPRE-1
TERG_08498	triosephosphate isomerase	CAGAGTG	136	UPRE-1
TERG_08498	triosephosphate isomerase	CAGGGTG	766	UPRE-1
TERG_08501	serine/threonine protein kinase	CAGGGTG	351	UPRE-1
TERG_08504	helicase (*T. equinum*)	CACCCTG (rc)	100	UPRE-1
TERG_08508	hypothetical protein	CAGCGTG	737	UPRE-1
TERG_08511	hypothetical protein	CACTCTG (rc)	286	UPRE-1
TERG_08525	hypothetical protein	CAGAGTG	460	UPRE-1
TERG_08528	fatty acid synthase beta subunit dehydratase (*T. tonsurans*)	CAGGGTG	48	UPRE-1
TERG_08542	GTP-binding protein ypt2	CACCCTG (rc)	771	UPRE-1
TERG_08545	C-4 methyl sterol oxidase Erg25 (*T. equinum*)	CAGCGTG	294	UPRE-1
TERG_08550	tRNA-specific adenosine deaminase subunit Tad2p/ADAT2 (*T. tonsurans*)	CAGGGTG	781	UPRE-1
TERG_08561	FGGY-family carbohydrate kinase (*T. tonsurans*)	CACGCTG (rc)	13	UPRE-1
TERG_08563	hypothetical protein	CACGCTG (rc)	542	UPRE-1
TERG_08589	cysteine-tRNA ligase	CACGCTG (rc)	154	UPRE-1
TERG_08591	multidrug resistance protein (*T. tonsurans*)	CAGTGTG	260	UPRE-1
TERG_08591	multidrug resistance protein (*T. tonsurans*)	CAGTGTG	871	UPRE-1
TERG_08592	short-chain oxidoreductase, putative (*A. benhamiae*)	CAGCGTG	8	UPRE-1
TERG_08598	eukaryotic translation initiation factor 6	CACTCTG (rc)	425	UPRE-1
TERG_08627	C6 transcription factor GliZ2 (*T. equinum*)	CACACTG (rc)	315	UPRE-1
TERG_08648	cell division control protein (Cdc15), putative (*A. benhamiae*)	CAGGGTG	226	UPRE-1
TERG_08655	ADP,ATP carrier protein	CACGCTG (rc)	203	UPRE-1

TERG_08662	hypothetical protein	CACACTG (rc)	840	UPRE-1
TERG_08666	hypothetical protein	CACACTG (rc)	537	UPRE-1
TERG_08672	HFR-3 (*M. canis*)	CACACTG (rc)	811	UPRE-1
TERG_08680	amino acid permease (*T. tonsurans*)	CACTCTG (rc)	49	UPRE-1
TERG_08692	GATA transcriptional activator AreA (*A. benhamiae*)	CAGAGTG	632	UPRE-1
TERG_08711	hypothetical protein	CAGCGTG	475	UPRE-1
TERG_08717	tRNA methyltransferase (*T. tonsurans*)	CAGGGTG	151	UPRE-1
TERG_08724	hypothetical protein	CAGAGTG	672	UPRE-1
TERG_08742	hypothetical protein	CACGCTG (rc)	958	UPRE-1
TERG_08744	hypothetical protein	CAGAGTG	160	UPRE-1
TERG_08749	pH signal transduction protein PalI, putative (*A. benhamiae*)	CACACTG (rc)	933	UPRE-1
TERG_08751	ABC multidrug transporter, putative (*A. benhamiae*)	CACACTG (rc)	705	UPRE-1
TERG_08756	cercosporin toxin biosynthesis protein (*T. equinum*)	CACACTG (rc)	476	UPRE-1
TERG_08771	extracellular serine-threonine rich protein (*T. tonsurans*)	CACCCTG (rc)	418	UPRE-1
TERG_08794	pyruvate dehydrogenase kinase (*T. equinum*)	CACACTG (rc)	941	UPRE-1
TERG_08831	acetyltransferase (*T. tonsurans*)	CAGAGTG	845	UPRE-1
TERG_08839	hypothetical protein	CACCCTG (rc)	609	UPRE-1
TERG_08843	hypothetical protein	CAGTGTG	64	UPRE-1
TERG_08845	pyridoxamine phosphate oxidase (*T. tonsurans*)	CACCCTG (rc)	272	UPRE-1
TERG_08878	hypothetical protein	CACCCTG (rc)	618	UPRE-1
TERG_08883	hypothetical protein	CACTCTG (rc)	299	UPRE-1
TERG_08909	hypothetical protein	CACACTG (rc)	376	UPRE-1
TERG_08919	hypothetical protein	CACCCTG (rc)	15	UPRE-1
TERG_08952	long-chain-fatty-acid-CoA ligase (*T. equinum*)	CAGGGTG	340	UPRE-1
TERG_08954	hypothetical protein	CACCCTG (rc)	699	UPRE-1
TERG_08960	SET domain protein (*T. verrucosum*)	CACGCTG (rc)	367	UPRE-1
TERG_08961	hypothetical protein	CAGCGTG	132	UPRE-1
TERG_08964	hypothetical protein	CAGGGTG	265	UPRE-1
TERG_08975	importin beta-1 subunit (*T. tonsurans*)	CACCCTG (rc)	385	UPRE-1
TERG_08987	hypothetical protein	CACCCTG (rc)	613	UPRE-1
TERG_08992	hypothetical protein	CAGAGTG	467	UPRE-1
TERG_11504	hypothetical protein	CAGGGTG	70	UPRE-1
TERG_11504	hypothetical protein	CAGGGTG	96	UPRE-1
TERG_11504	hypothetical protein	CAGAGTG	825	UPRE-1
TERG_11505	phosphoenolpyruvate carboxykinase [ATP]	CAGAGTG	68	UPRE-1
TERG_11508	C6 finger domain protein, putative (*A. benhamiae*)	CACGCTG (rc)	4	UPRE-1

TERG_11509	hypothetical protein	CAGCGTG	297	UPRE-1
TERG_11511	hypothetical protein	CAGAGTG	870	UPRE-1
TERG_11514	lipase/esterase, putative (*T. verrucosum*)	CAGTGTG	412	UPRE-1
TERG_11517	hypothetical protein	CAGGGTG	458	UPRE-1
TERG_11524	hypothetical protein	CAGCGTG	657	UPRE-1
TERG_11527	tenascin C (*T. equinum*)	CAGTGTG	694	UPRE-1
TERG_11528	tenascin C (*T. equinum*)	CAGTGTG	307	UPRE-1
TERG_11528	tenascin C (*T. equinum*)	CAGTGTG	650	UPRE-1
TERG_11528	tenascin C (*T. equinum*)	CAGTGTG	781	UPRE-1
TERG_11528	tenascin C (*T. equinum*)	CAGTGTG	856	UPRE-1
TERG_11528	tenascin C (*T. equinum*)	CAGTGTG	931	UPRE-1
TERG_11533	hypothetical protein	CAGAGTG	719	UPRE-1
TERG_11535	hypothetical protein	CAGAGTG	933	UPRE-1
TERG_11536	hypothetical protein	CAGGGTG	918	UPRE-1
TERG_11538	3-oxoacyl-(acyl-carrier-protein) reductase (*T. tonsurans*)	CAGGGTG	348	UPRE-1
TERG_11552	hypothetical protein	CAGAGTG	608	UPRE-1
TERG_11556	hypothetical protein	CAGTGTG	726	UPRE-1
TERG_11557	luciferin 4-monooxygenase (*T. equinum*)	CAGCGTG	550	UPRE-1
TERG_11569	uridine nucleosidase (*T. tonsurans*)	CAGTGTG	504	UPRE-1
TERG_11575	phosphotransferase enzyme family protein (*T. equinum*)	CAGAGTG	4	UPRE-1
TERG_11584	hexose transporter protein (*A. benhamiae*)	CAGGGTG	422	UPRE-1
TERG_11595	LipA and NB-ARC domain-containing protein (*M. canis*)	CAGTGTG	29	UPRE-1
TERG_11619	hypothetical protein	CAGTGTG	454	UPRE-1
TERG_11622	hypothetical protein	CAGGGTG	613	UPRE-1
TERG_11656	class V chitinase (*T. tonsurans*)	CAGTGTG	169	UPRE-1
TERG_11683	NADH-ubiquinone oxidoreductase 40 kDa subunit, mitochondrial	CACACTG (rc)	558	UPRE-1
TERG_11686	hypothetical protein	CAGGGTG	40	UPRE-1
TERG_11697	hypothetical protein	CACGCTG (rc)	100	UPRE-1
TERG_11701	hypothetical protein	CACTCTG (rc)	51	UPRE-1
TERG_11706	hypothetical protein	CACCCTG (rc)	278	UPRE-1
TERG_11708	hypothetical protein	CAGTGTG	513	UPRE-1
TERG_11725	C6 transcription factor, putative (*T. verrucosum*)	CAGTGTG	478	UPRE-1
TERG_11731	zinc/cadmium resistance protein (*T. tonsurans*)	CACTCTG (rc)	683	UPRE-1
TERG_11738	cytoskeleton organization protein (Dec1), putative (*T. verrucosum*)	CAGCGTG	451	UPRE-1

TERG_11739	cytoskeleton organization protein (Dec1), putative (*T. verrucosum*)	CAGCGTG	39	UPRE-1
TERG_11739	cytoskeleton organization protein (Dec1), putative (*T. verrucosum*)	CAGCGTG	855	UPRE-1
TERG_11744	arylsulfatase, putative (*T. verrucosum*)	CAGGGTG	124	UPRE-1
TERG_11749	hypothetical protein	CAGAGTG	134	UPRE-1
TERG_11749	hypothetical protein	CAGTGTG	720	UPRE-1
TERG_11754	hypothetical protein	CAGGGTG	384	UPRE-1
TERG_11767	amidase family protein, putative (*T. verrucosum*)	CAGTGTG	331	UPRE-1
TERG_11772	TKL protein kinase (*T. tonsurans*)	CAGAGTG	705	UPRE-1
TERG_11772	TKL protein kinase (*T. tonsurans*)	CAGCGTG	827	UPRE-1
TERG_11775	hypothetical protein	CAGGGTG	353	UPRE-1
TERG_11781	hypothetical protein	CAGGGTG	454	UPRE-1
TERG_11782	hypothetical protein	CAGTGTG	98	UPRE-1
TERG_11784	extracellular metalloproteinase 5	CAGGGTG	685	UPRE-1
TERG_11786	oligosaccharyl transferase stt3 subunit (*T. equinum*)	CAGCGTG	859	UPRE-1
TERG_11787	oligosaccharyl transferase stt3 subunit (*T. equinum*)	CAGCGTG	378	UPRE-1
TERG_11789	DNA mismatch repair protein Msh1 (*T. equinum*)	CAGAGTG	550	UPRE-1
TERG_11796	hypothetical protein	CACGCTG (rc)	34	UPRE-1
TERG_11797	hypothetical protein	CACGCTG (rc)	673	UPRE-1
TERG_11814	FAD dependent oxidoreductase, putative (*A. benhamiae*)	CAGCGTG	440	UPRE-1
TERG_11821	hypothetical protein	CAGGGTG	50	UPRE-1
TERG_11821	hypothetical protein	CAGAGTG	836	UPRE-1
TERG_11831	hypothetical protein	CACCCTG (rc)	316	UPRE-1
TERG_11831	hypothetical protein	CACACTG (rc)	466	UPRE-1
TERG_11844	RNA polymerase II mediator complex component Srb8 (*T. equinum*)	CAGGGTG	75	UPRE-1
TERG_11851	hypothetical protein	CAGAGTG	704	UPRE-1
TERG_11865	hypothetical protein	CACGCTG (rc)	49	UPRE-1
TERG_11865	hypothetical protein	CACGCTG (rc)	181	UPRE-1
TERG_11868	MFS monosaccharide transporter (*T. tonsurans*)	CAGGGTG	195	UPRE-1
TERG_11880	hypothetical protein	CAGGGTG	40	UPRE-1
TERG_11885	ABC bile acid transporter, putative (*T. verrucosum*)	CAGAGTG	12	UPRE-1
TERG_11890	hypothetical protein	CAGGGTG	182	UPRE-1
TERG_11897	hypothetical protein	CAGTGTG	585	UPRE-1
TERG_11904	hypothetical protein	CACTCTG (rc)	916	UPRE-1
TERG_11905	hypothetical protein	CAGAGTG	435	UPRE-1

TERG_11914	Protein phosphatase 2C, putative (*T. verrucosum*)	CAGAGTG	431	UPRE-1
TERG_11924	ankyrin repeat protein (*T. tonsurans*)	CAGGGTG	687	UPRE-1
TERG_11925	tRNA methyltransferase (*T. tonsurans*)	CAGGGTG	173	UPRE-1
TERG_11933	Cytochrome b5 (*T. tonsurans*)	CACCCTG (rc)	662	UPRE-1
TERG_11934	PAB-dependent poly(A)-specific ribonuclease subunit PAN2	CAGAGTG	377	UPRE-1
TERG_11943	MFS maltose permease (*T. tonsurans*)	CACGCTG (rc)	757	UPRE-1
TERG_11945	hypothetical protein	CACTCTG (rc)	126	UPRE-1
TERG_11946	hypothetical protein	CAGAGTG	774	UPRE-1
TERG_11948	hypothetical protein	CAGAGTG	979	UPRE-1
TERG_11949	hypothetical protein	CAGGGTG	65	UPRE-1
TERG_11952	mandelate racemase/muconate lactonizing enzyme (*T. tonsurans*)	CAGAGTG	95	UPRE-1
TERG_11954	hypothetical protein	CAGCGTG	23	UPRE-1
TERG_11960	CMGC/CDK/PITSLRE protein kinase	CAGCGTG	985	UPRE-1
TERG_11962	DEAD/DEAH box RNA helicase (*T. tonsurans*)	CAGCGTG	310	UPRE-1
TERG_11968	hypothetical protein	CAGGGTG	650	UPRE-1
TERG_11975	integral membrane protein (*T. verrucosum*)	CACTCTG (rc)	468	UPRE-1
TERG_11976	integral membrane protein (*T. tonsurans*)	CACCCTG (rc)	480	UPRE-1
TERG_11982	ankyrin repeat protein (*T. verrucosum*)	CACGCTG (rc)	223	UPRE-1
TERG_11985	vacuolar fusion protein mon1	CAGAGTG	408	UPRE-1
TERG_11994	hypothetical protein	CAGGGTG	499	UPRE-1
TERG_11996	chitinase (*M. canis*)	CAGGGTG	830	UPRE-1
TERG_12004	lipase (*T. tonsurans*)	CACGCTG (rc)	985	UPRE-1
TERG_12011	hypothetical protein	CAGGGTG	8	UPRE-1
TERG_12016	hypothetical protein	CAGCGTG	929	UPRE-1
TERG_12021	Zn cluster transcription factor Rds2 (*T. tonsurans*)	CACGCTG (rc)	636	UPRE-1
TERG_12022	hypothetical protein	CAGCGTG	238	UPRE-1
TERG_12032	hypothetical protein	CACCCTG (rc)	450	UPRE-1
TERG_12034	hypothetical protein	CAGGGTG	230	UPRE-1
TERG_12039	hypothetical protein	CACGCTG (rc)	700	UPRE-1
TERG_12041	priB protein (*M. gypseum*)	CACCCTG (rc)	707	UPRE-1
TERG_12042	RNA helicase/RNAse III, putative (*A. benhamiae*)	CACACTG (rc)	853	UPRE-1
TERG_12042	RNA helicase/RNAse III, putative (*A. benhamiae*)	CACGCTG (rc)	927	UPRE-1
TERG_12045	hypothetical protein	CAGAGTG	987	UPRE-1
TERG_12048	guanyl-nucleotide exchange factor Sec7 (*T. equinum*)	CAGAGTG	366	UPRE-1
TERG_12048	guanyl-nucleotide exchange factor Sec7 (*T. equinum*)	CAGGGTG	706	UPRE-1
TERG_12049	DUF890 domain-containing protein (*T. equinum*)	CAGCGTG	698	UPRE-1

TERG_12054	HEAT repeat protein (*A. benhamiae*)	CACGCTG (rc)	304	UPRE-1
TERG_12056	hypothetical protein	CAGGGTG	281	UPRE-1
TERG_12058	transporter smf2 (*T. tonsurans*)	CAGGGTG	213	UPRE-1
TERG_12071	AGC/DMPK protein kinase (*T. equinum*)	CACTCTG (rc)	521	UPRE-1
TERG_12082	hypothetical protein	CAGAGTG	791	UPRE-1
TERG_12099	hypothetical protein	CAGTGTG	83	UPRE-1
TERG_12102	hypothetical protein	CACTCTG (rc)	805	UPRE-1
TERG_12104	hypothetical protein	CAGGGTG	465	UPRE-1
TERG_12105	amino-acid N-acetyltransferase subunit Mak10 (*T. tonsurans*)	CACACTG (rc)	99	UPRE-1
TERG_12107	1,4-alpha-glucan-branching enzyme	CAGCGTG	555	UPRE-1
TERG_12112	hypothetical protein	CACACTG (rc)	779	UPRE-1
TERG_12121	DNA replication licensing factor mcm5 (*T. tonsurans*)	CAGCGTG	459	UPRE-1
TERG_12127	phosphatidylinositol?glycan (*T. equinum*)	CAGTGTG	207	UPRE-1
TERG_12130	hypothetical protein	CAGGGTG	693	UPRE-1
TERG_12132	hypothetical protein	CAGAGTG	561	UPRE-1
TERG_12138	hypothetical protein	CAGGGTG	733	UPRE-1
TERG_12139	hypothetical protein	CAGGGTG	224	UPRE-1
TERG_12143	hypothetical protein	CAGCGTG	234	UPRE-1
TERG_12145	histone deacetylase HdaA (*T. tonsurans*)	CACACTG (rc)	645	UPRE-1
TERG_12160	STE/STE11/CDC15 protein kinase (*T. tonsurans*)	CAGCGTG	572	UPRE-1
TERG_12163	RSC complex subunit Sfh1 (*T. tonsurans*)	CAGGGTG	215	UPRE-1
TERG_12165	PX domain protein (*T. verrucosum*)	CACTCTG (rc)	701	UPRE-1
TERG_12170	WD domain-containing protein (*T. equinum*)	CACTCTG (rc)	639	UPRE-1
TERG_12172	glycerol-3-phosphate dehydrogenase (*T. equinum*)	CACACTG (rc)	110	UPRE-1
TERG_12175	hypothetical protein	CAGCGTG	320	UPRE-1
TERG_12176	hypothetical protein	CAGGGTG	18	UPRE-1
TERG_12192	hypothetical protein	CAGTGTG	671	UPRE-1
TERG_12198	hypothetical protein	CACCCTG (rc)	190	UPRE-1
TERG_12200	hypothetical protein	CAGGGTG	214	UPRE-1
TERG_12201	hypothetical protein	CACTCTG (rc)	963	UPRE-1
TERG_12204	RNA polymerase I specific transcription initiation factor Rrn7, putative (*T. verrucosum*)	CAGAGTG	676	UPRE-1
TERG_12206	translation regulator (Cya5), putative (*T. verrucosum*)	CAGCGTG	1	UPRE-1
TERG_12212	hypothetical protein	CAGAGTG	292	UPRE-1
TERG_12213	hypothetical protein	CAGGGTG	641	UPRE-1
TERG_12265	glutathione-dependent formaldehyde-activating enzyme (*T. tonsurans*)	CAGCGTG	333	UPRE-1

TERG_12285	hypothetical protein	CAGAGTG	310	UPRE-1
TERG_12290	mitochondrial cytochrome b2 (*T. tonsurans*)	CACGCTG (rc)	328	UPRE-1
TERG_12290	mitochondrial cytochrome b2 (*T. tonsurans*)	CACCCTG (rc)	837	UPRE-1
TERG_12292	hypothetical protein	CAGAGTG	931	UPRE-1
TERG_12293	hypothetical protein	CAGCGTG	580	UPRE-1
TERG_12298	hypothetical protein	CAGTGTG	877	UPRE-1
TERG_12302	hypothetical protein	CAGCGTG	619	UPRE-1
TERG_12310	null (*T. verrucosum*)	CACACTG (rc)	24	UPRE-1
TERG_12316	phosphotransferase enzyme family protein (*T. equinum*)	CAGGGTG	828	UPRE-1
TERG_12317	phosphotransferase enzyme family protein (*T. equinum*)	CAGGGTG	78	UPRE-1
TERG_12324	hypothetical protein	CAGGGTG	316	UPRE-1
TERG_12325	hypothetical protein	CAGGGTG	416	UPRE-1
TERG_12334	hypothetical protein	CAGAGTG	35	UPRE-1
TERG_12335	proliferating cell nuclear antigen (pcna)	CAGCGTG	127	UPRE-1
TERG_12342	membrane zinc transporter (*T. tonsurans*)	CAGCGTG	195	UPRE-1
TERG_12347	Anaphase promoting complex subunit Apc11, putative (*T. tonsurans*)	CACCCTG (rc)	193	UPRE-1
TERG_12348	anaphase promoting complex subunit Apc11 (*T. equinum*)	CACGCTG (rc)	489	UPRE-1
TERG_12348	anaphase promoting complex subunit Apc11 (*T. equinum*)	CACCCTG (rc)	943	UPRE-1
TERG_12362	mitochondrial molecular chaperone (*T. tonsurans*)	CACACTG (rc)	828	UPRE-1
TERG_12372	multidrug resistance protein (*T. tonsurans*)	CAGGGTG	964	UPRE-1
TERG_12374	UDP-N-acetylglucosaminyltransferase (*T. equinum*)	CACCCTG (rc)	140	UPRE-1
TERG_12412	MFS sugar transporter, putative (*T. verrucosum*)	CAGCGTG	211	UPRE-1
TERG_12412	MFS sugar transporter, putative (*T. verrucosum*)	CAGAGTG	263	UPRE-1
TERG_12419	uracil permease (*M. gypseum*)	CAGGGTG	698	UPRE-1
TERG_12422	SPX domain-containing protein (*T. tonsurans*)	CAGCGTG	628	UPRE-1
TERG_12426	T-complex protein 1 (*T. tonsurans*)	CACCCTG (rc)	159	UPRE-1
TERG_12442	hypothetical protein	CACGCTG (rc)	936	UPRE-1
TERG_12443	hypothetical protein	CAGAGTG	31	UPRE-1
TERG_12454	cutinase, putative (*A. benhamiae*)	CAGGGTG	102	UPRE-1
TERG_12454	cutinase, putative (*A. benhamiae*)	CAGAGTG	892	UPRE-1
TERG_12454	cutinase, putative (*A. benhamiae*)	CAGGGTG	950	UPRE-1
TERG_12456	hypothetical protein	CACTCTG (rc)	422	UPRE-1
TERG_12460	hypothetical protein	CAGGGTG	894	UPRE-1
TERG_12461	HMG-CoA reductase (*A. benhamiae*)	CAGGGTG	642	UPRE-1
TERG_12473	FYVE zinc finger protein (*T. equinum*)	CACGCTG (rc)	726	UPRE-1
TERG_12488	rRNA processing protein (*T. tonsurans*)	CAGTGTG	6	UPRE-1

TERG_12491	carboxylesterase, putative (*A. benhamiae*)	CAGGGTG	415	UPRE-1
TERG_12500	sorbitol dehydrogenase (*T. equinum*)	CAGCGTG	239	UPRE-1
TERG_12501	catechol dioxygenase, putative (*T. verrucosum*)	CAGCGTG	274	UPRE-1
TERG_12501	catechol dioxygenase, putative (*T. verrucosum*)	CAGGGTG	364	UPRE-1
TERG_12518	phosphotransferase enzyme family protein (*T. equinum*)	CAGAGTG	576	UPRE-1
TERG_12522	4-hydroxyphenylpyruvate dioxygenase (*T. tonsurans*)	CAGTGTG	264	UPRE-1
TERG_12534	glycerol-3-phosphate O-acyltransferase (*T. equinum*)	CACCCTG (rc)	278	UPRE-1
TERG_12534	glycerol-3-phosphate O-acyltransferase (*T. equinum*)	CACTCTG (rc)	680	UPRE-1
TERG_12541	hypothetical protein	CAGGGTG	93	UPRE-1
TERG_12543	hypothetical protein	CAGAGTG	549	UPRE-1
TERG_12544	hypothetical protein	CAGCGTG	259	UPRE-1
TERG_12544	hypothetical protein	CAGGGTG	921	UPRE-1
TERG_12546	chromosome segregation protein BIR1 (*T. equinum*)	CACACTG (rc)	86	UPRE-1
TERG_12547	chromosome segregation protein BIR1 (*T. equinum*)	CACACTG (rc)	610	UPRE-1
TERG_12555	hypothetical protein	CAGGGTG	585	UPRE-1
TERG_12556	hypothetical protein	CAGAGTG	561	UPRE-1
TERG_12562	hypothetical protein	CAGTGTG	446	UPRE-1
TERG_12566	hypothetical protein	CAGTGTG	707	UPRE-1
TERG_12571	ATP-dependent RNA helicase DOB1 (*T. equinum*)	CAGTGTG	661	UPRE-1
TERG_12571	ATP-dependent RNA helicase DOB1 (*T. equinum*)	CAGAGTG	943	UPRE-1
TERG_12579	MFS transporter (*T. tonsurans*)	CAGTGTG	596	UPRE-1
TERG_12582	hypothetical protein	CAGCGTG	529	UPRE-1
TERG_12582	hypothetical protein	CAGCGTG	954	UPRE-1
TERG_12588	spindle pole body associated protein SnaD, putative (*T. verrucosum*)	CAGGGTG	937	UPRE-1
TERG_12595	ABC multidrug transporter SitT (*T. tonsurans*)	CAGCGTG	58	UPRE-1
TERG_12596	hypothetical protein	CAGGGTG	936	UPRE-1
TERG_12597	hypothetical protein	CAGGGTG	151	UPRE-1
TERG_12598	hypothetical protein	CAGAGTG	600	UPRE-1
TERG_12599	hypothetical protein	CAGGGTG	154	UPRE-1
TERG_12603	hypothetical protein	CACTCTG (rc)	202	UPRE-1
TERG_12606	protease DPPV, putative (*A. benhamiae*)	CACACTG (rc)	281	UPRE-1
TERG_12614	succinyl-CoA synthetase beta subunit, putative (*T. verrucosum*)	CAGAGTG	890	UPRE-1
TERG_12620	hypothetical protein	CAGAGTG	513	UPRE-1
TERG_12630	nicotinamide nucleotide transhydrogenase (*T. tonsurans*)	CAGGGTG	711	UPRE-1
TERG_12647	hypothetical protein	CAGGGTG	435	UPRE-1

TERG_12656	endoribonuclease ysh1 (*T. tonsurans*)	CACTCTG (rc)	383	UPRE-1
TERG_12672	hypothetical protein	CAGAGTG	136	UPRE-1
TERG_12672	hypothetical protein	CAGAGTG	458	UPRE-1
TERG_12673	hypothetical protein	CAGGGTG	282	UPRE-1
TERG_12684	hypothetical protein	CACGCTG (rc)	380	UPRE-1
TERG_12688	Cullin family protein (*T. equinum*)	CAGAGTG	963	UPRE-1
TERG_12692	hypothetical protein	CAGCGTG	944	UPRE-1
TERG_12704	hypothetical protein	CACACTG (rc)	167	UPRE-1
TERG_12712	hypothetical protein	CACTCTG (rc)	197	UPRE-1
TERG_12712	hypothetical protein	CACGCTG (rc)	366	UPRE-1
TERG_12721	transferase (*M. canis*)	CAGTGTG	188	UPRE-1
TERG_12725	MFS transporter (*T. equinum*)	CAGTGTG	219	UPRE-1
TERG_12727	cytochrome P450 monooxigenase GliC2 (*T. equinum*)	CAGAGTG	554	UPRE-1
TERG_00009	peroxisomal biogenesis factor (*T. tonsurans*)	TACGTG	310	UPRE-2
TERG_00019	mitochondrial Rho GTPase 1	CACGTA (rc)	174	UPRE-2
TERG_00055	essential cytoplasmic protein Ctr86 (*T. equinum*)	TACGTG	100	UPRE-2
TERG_00057	hypothetical protein	CACGTA (rc)	677	UPRE-2
TERG_00061	GDP-mannose pyrophosphorylase A (*T. equinum*)	CACGTA (rc)	648	UPRE-2
TERG_00062	dynamin GTPase, putative (*T. verrucosum*)	TACGTG	368	UPRE-2
TERG_00063	lysine-tRNA ligase	TACGTG	121	UPRE-2
TERG_00065	cytochrome c oxidase assembly protein COX15 (*T. equinum*)	CACGTA (rc)	748	UPRE-2
TERG_00073	NADH-dependent flavin oxidoreductase, putative (*T. verrucosum*)	TACGTG	131	UPRE-2
TERG_00117	SNF2 family helicase, putative (*T. verrucosum*)	CACGTA (rc)	868	UPRE-2
TERG_00141	DUF803 domain-containing protein (*T. equinum*)	CACGTA (rc)	533	UPRE-2
TERG_00159	hypothetical protein	TACGTG	596	UPRE-2
TERG_00227	glutamine amidotransferase subunit pdxT (*T. equinum*)	TACGTG	731	UPRE-2
TERG_00250	phosphoacetylglucosamine mutase (*T. equinum*)	CACGTA (rc)	111	UPRE-2
TERG_00256	26S protease regulatory subunit 6A	TACGTG	799	UPRE-2
TERG_00266	hypothetical protein	CACGTA (rc)	327	UPRE-2
TERG_00274	mitochondrial methyltransferase (predicted) (*A. benhamiae*)	CACGTA (rc)	349	UPRE-2
TERG_00276	TATA-box-binding protein	CACGTA (rc)	379	UPRE-2
TERG_00278	electron transfer flavoprotein-ubiquinone oxidoreductase (*T. equinum*)	TACGTG	187	UPRE-2
TERG_00292	hypothetical protein	CACGTA (rc)	926	UPRE-2
TERG_00310	dolichol-phosphate mannosyltransferase (*T. tonsurans*)	CACGTA (rc)	800	UPRE-2
TERG_00311	hypothetical protein	TACGTG	628	UPRE-2

TERG_00322	hypothetical protein	CACGTA (rc)	401	UPRE-2
TERG_00323	DNA damage response protein (*T. tonsurans*)	TACGTG	493	UPRE-2
TERG_00331	golgi apparatus membrane protein TVP18 (*T. tonsurans*)	CACGTA (rc)	244	UPRE-2
TERG_00340	hypothetical protein	TACGTG	145	UPRE-2
TERG_00357	SNARE-dependent exocytosis protein (*T. tonsurans*)	TACGTG	354	UPRE-2
TERG_00357	SNARE-dependent exocytosis protein (*T. tonsurans*)	TACGTG	842	UPRE-2
TERG_00372	hypothetical protein	CACGTA (rc)	615	UPRE-2
TERG_00390	AP-1 complex subunit gamma-1 (*T. tonsurans*)	CACGTA (rc)	144	UPRE-2
TERG_00417	zinc-binding oxidoreductase, putative (*A. benhamiae*)	CACGTA (rc)	92	UPRE-2
TERG_00417	zinc-binding oxidoreductase, putative (*A. benhamiae*)	CACGTA (rc)	209	UPRE-2
TERG_00474	tRNA-specific adenosine deaminase (*T. equinum*)	TACGTG	518	UPRE-2
TERG_00504	hypothetical protein	CACGTA (rc)	15	UPRE-2
TERG_00524	trans-sialidase (*M. canis*)	CACGTA (rc)	988	UPRE-2
TERG_00525	NAK protein kinase	TACGTG	93	UPRE-2
TERG_00548	elongation factor 1-alpha	CACGTA (rc)	338	UPRE-2
TERG_00570	autophagy protein (*T. equinum*)	CACGTA (rc)	627	UPRE-2
TERG_00577	BAR domain-containing protein (*T. tonsurans*)	CACGTA (rc)	102	UPRE-2
TERG_00579	glutathione S-transferase (*T. tonsurans*)	TACGTG	525	UPRE-2
TERG_00579	glutathione S-transferase (*T. tonsurans*)	TACGTG	945	UPRE-2
TERG_00587	nuclear pore complex protein sonA (*T. equinum*)	CACGTA (rc)	875	UPRE-2
TERG_00596	M protein repeat-containing protein (*T. equinum*)	TACGTG	713	UPRE-2
TERG_00608	hypothetical protein	CACGTA (rc)	239	UPRE-2
TERG_00609	ankyrin repeat protein (*T. equinum*)	TACGTG	685	UPRE-2
TERG_00644	ER membrane DUF1077 domain-containing protein (*T. equinum*)	CACGTA (rc)	394	UPRE-2
TERG_00646	integral membrane protein (*T. verrucosum*)	TACGTG	850	UPRE-2
TERG_00651	phosphatidylinositol 3-kinase (*T. tonsurans*)	CACGTA (rc)	802	UPRE-2
TERG_00669	C-5 sterol desaturase (*T. tonsurans*)	CACGTA (rc)	305	UPRE-2
TERG_00670	hypothetical protein	TACGTG	195	UPRE-2
TERG_00681	5-oxoprolinase (*T. tonsurans*)	TACGTG	602	UPRE-2
TERG_00697	nonribosomal siderophore peptide synthase SidC (*A. benhamiae*)	CACGTA (rc)	480	UPRE-2
TERG_00707	GPI anchored serine-threonine rich protein (*T. verrucosum*)	CACGTA (rc)	373	UPRE-2
TERG_00725	hypothetical protein	TACGTG	924	UPRE-2
TERG_00738	60S ribosomal protein L44	CACGTA (rc)	115	UPRE-2
TERG_00740	rds1 (*T. equinum*)	TACGTG	33	UPRE-2

TERG_00746	vacuolar carboxypeptidase Cps1, putative (*A. benhamiae*)	TACGTG	347	UPRE-2
TERG_00761	homocitrate synthase (*T. equinum*)	CACGTA (rc)	163	UPRE-2
TERG_00761	homocitrate synthase (*T. equinum*)	CACGTA (rc)	498	UPRE-2
TERG_00762	vesicular-fusion protein SEC18 (*M. gypseum*)	TACGTG	771	UPRE-2
TERG_00799	Haloacid dehalogenase (*T. tonsurans*)	CACGTA (rc)	973	UPRE-2
TERG_00800	hypothetical protein	TACGTG	708	UPRE-2
TERG_00804	U1 small nuclear ribonucleoprotein C (*T. tonsurans*)	CACGTA (rc)	378	UPRE-2
TERG_00805	benzoate 4-monooxygenase cytochrome P450 (*T. equinum*)	CACGTA (rc)	516	UPRE-2
TERG_00809	glycosyltransferase family 28 protein, putative (*A. benhamiae*)	CACGTA (rc)	820	UPRE-2
TERG_00824	neutral trehalase	TACGTG	534	UPRE-2
TERG_00883	acyl carrier protein	CACGTA (rc)	412	UPRE-2
TERG_00888	hypothetical protein	CACGTA (rc)	744	UPRE-2
TERG_00900	BCAS2 domain containing protein (*T. equinum*)	TACGTG	317	UPRE-2
TERG_00929	N-acetylglucosaminyl transferase component Gpi1 (*T. tonsurans*)	CACGTA (rc)	131	UPRE-2
TERG_00940	rRNA processing protein Utp6 (*T. tonsurans*)	CACGTA (rc)	699	UPRE-2
TERG_00954	hypothetical protein	TACGTG	888	UPRE-2
TERG_00955	ABC drug exporter AtrF (*T. verrucosum*)	TACGTG	515	UPRE-2
TERG_00957	transporter sec-24 (*T. equinum*)	CACGTA (rc)	296	UPRE-2
TERG_00957	transporter sec-24 (*T. equinum*)	CACGTA (rc)	454	UPRE-2
TERG_00993	allantoinase (*T. tonsurans*)	CACGTA (rc)	560	UPRE-2
TERG_00995	hypothetical protein	CACGTA (rc)	63	UPRE-2
TERG_01010	Leucine Rich Repeat domain protein (*A. benhamiae*)	CACGTA (rc)	870	UPRE-2
TERG_01019	hypothetical protein	TACGTG	19	UPRE-2
TERG_01038	hypothetical protein	TACGTG	29	UPRE-2
TERG_01039	DNA topoisomerase III (*T. tonsurans*)	CACGTA (rc)	791	UPRE-2
TERG_01040	DUF1295 domain-containing protein (*T. equinum*)	TACGTG	926	UPRE-2
TERG_01079	prenyltransferase (*T. equinum*)	CACGTA (rc)	180	UPRE-2
TERG_01080	di-trans,poly-cis-decaprenylcistransferase	TACGTG	871	UPRE-2
TERG_01134	metalloprotease MEP1 (*A. benhamiae*)	TACGTG	393	UPRE-2
TERG_01157	rhomboid family protein (*T. tonsurans*)	TACGTG	8	UPRE-2
TERG_01198	pre-mRNA-splicing factor rse1	CACGTA (rc)	350	UPRE-2
TERG_01241	ClpTM1 domain-containing protein (*T. tonsurans*)	CACGTA (rc)	588	UPRE-2
TERG_01250	hypothetical protein	TACGTG	271	UPRE-2
TERG_01261	serine/threonine protein kinase	CACGTA (rc)	231	UPRE-2
TERG_01274	mRNA export factor elf1 (*T. equinum*)	TACGTG	538	UPRE-2

TERG_01276	amino acid permease family protein, putative (*A. benhamiae*)	TACGTG	136	UPRE-2
TERG_01277	nexin-1 (*T. equinum*)	TACGTG	36	UPRE-2
TERG_01302	lipase, putative (*T. verrucosum*)	TACGTG	119	UPRE-2
TERG_01346	lipase/serine esterase (*T. tonsurans*)	TACGTG	174	UPRE-2
TERG_01349	glutathione peroxidase (*T. tonsurans*)	TACGTG	468	UPRE-2
TERG_01354	V-type proton ATPase proteolipid subunit	TACGTG	278	UPRE-2
TERG_01369	ubiquinol-cytochrome C reductase complex subunit UcrQ (*T. tonsurans*)	CACGTA (rc)	90	UPRE-2
TERG_01371	hypothetical protein	TACGTG	86	UPRE-2
TERG_01377	hypothetical protein	CACGTA (rc)	18	UPRE-2
TERG_01379	serine/threonine-protein phosphatase 2A activator 2 (*T. equinum*)	TACGTG	334	UPRE-2
TERG_01401	high affinity copper transporter (*T. tonsurans*)	TACGTG	563	UPRE-2
TERG_01403	serine/threonine-protein phosphatase	TACGTG	146	UPRE-2
TERG_01405	glutathione S-transferase (*T. equinum*)	TACGTG	62	UPRE-2
TERG_01418	integral membrane protein, putative (*T. verrucosum*)	TACGTG	913	UPRE-2
TERG_01437	MFS transporter, putative (*T. verrucosum*)	CACGTA (rc)	365	UPRE-2
TERG_01455	hypothetical protein	TACGTG	726	UPRE-2
TERG_01468	hypothetical protein	CACGTA (rc)	217	UPRE-2
TERG_01470	serine/threonine protein kinase (*M. canis*)	TACGTG	947	UPRE-2
TERG_01477	cwl1 (*M. gypseum*)	CACGTA (rc)	703	UPRE-2
TERG_01505	hypothetical protein	TACGTG	758	UPRE-2
TERG_01512	mitochondrial tricarboxylate transporter (Ctp), putative (*T. verrucosum*)	TACGTG	307	UPRE-2
TERG_01520	hypothetical protein	TACGTG	692	UPRE-2
TERG_01522	kinesin heavy chain	TACGTG	679	UPRE-2
TERG_01537	hypothetical protein	CACGTA (rc)	276	UPRE-2
TERG_01546	oxidoreductase (*T. tonsurans*)	CACGTA (rc)	594	UPRE-2
TERG_01547	hypothetical protein	CACGTA (rc)	115	UPRE-2
TERG_01548	DNA polymerase subunit delta-2 (*T. tonsurans*)	TACGTG	80	UPRE-2
TERG_01555	L-serine dehydratase, putative (*T. verrucosum*)	TACGTG	188	UPRE-2
TERG_01560	conserved eukaryotic protein (*A. benhamiae*)	CACGTA (rc)	166	UPRE-2
TERG_01579	hypothetical protein	TACGTG	903	UPRE-2
TERG_01588	MAK11 (*T. equinum*)	CACGTA (rc)	237	UPRE-2
TERG_01634	MFS multidrug transporter, putative (*T. verrucosum*)	TACGTG	395	UPRE-2
TERG_01645	mitochondrial carrier protein (*T. tonsurans*)	CACGTA (rc)	385	UPRE-2
TERG_01674	hypothetical protein	CACGTA (rc)	628	UPRE-2

TERG_01675	proteasome subunit alpha type-3 (*T. tonsurans*)	TACGTG	164	UPRE-2
TERG_01694	ankyrin repeat-containing protein (*T. equinum*)	CACGTA (rc)	756	UPRE-2
TERG_01709	hypothetical protein	TACGTG	268	UPRE-2
TERG_01738	hypothetical protein	CACGTA (rc)	383	UPRE-2
TERG_01764	rab6 (*T. equinum*)	CACGTA (rc)	147	UPRE-2
TERG_01765	V-ATPase proteolipid subunit Ppa1 (*T. tonsurans*)	TACGTG	472	UPRE-2
TERG_01782	hypothetical protein	CACGTA (rc)	212	UPRE-2
TERG_01786	DENN domain-containing protein (*T. tonsurans*)	TACGTG	967	UPRE-2
TERG_01793	ATP-dependent RNA helicase DBP10	TACGTG	188	UPRE-2
TERG_01798	MATE efflux family protein subfamily, putative (*T. verrucosum*)	CACGTA (rc)	261	UPRE-2
TERG_01812	nucleolar complex protein 14 (*T. equinum*)	CACGTA (rc)	356	UPRE-2
TERG_01815	origin recognition complex subunit 3, putative (*A. benhamiae*)	TACGTG	720	UPRE-2
TERG_01857	mitochondrial import inner membrane translocase subunit Tim9 (*T. tonsurans*)	TACGTG	97	UPRE-2
TERG_01871	acetyl-CoA acetyltransferase (*T. tonsurans*)	CACGTA (rc)	467	UPRE-2
TERG_01872	polyketide synthase, putative (*T. verrucosum*)	CACGTA (rc)	633	UPRE-2
TERG_01915	O-methyltransferase (*A. benhamiae*)	CACGTA (rc)	628	UPRE-2
TERG_01931	bis(5'-nucleosyl)-tetraphosphatase (*T. equinum*)	CACGTA (rc)	947	UPRE-2
TERG_01936	COP9 signalosome complex subunit 1 (*T. equinum*)	CACGTA (rc)	637	UPRE-2
TERG_01936	COP9 signalosome complex subunit 1 (*T. equinum*)	CACGTA (rc)	860	UPRE-2
TERG_01985	hypothetical protein	CACGTA (rc)	668	UPRE-2
TERG_01986	Fe-S protein assembly co-chaperone HscB	CACGTA (rc)	430	UPRE-2
TERG_01996	serine/threonine protein kinase	CACGTA (rc)	115	UPRE-2
TERG_02025	hypothetical protein	TACGTG	900	UPRE-2
TERG_02046	cysteine synthase (*T. tonsurans*)	CACGTA (rc)	231	UPRE-2
TERG_02063	hypothetical protein	TACGTG	295	UPRE-2
TERG_02086	exocyst complex component Sec10 (*T. tonsurans*)	CACGTA (rc)	485	UPRE-2
TERG_02138	AGC protein kinase	TACGTG	104	UPRE-2
TERG_02221	proteasome component PRE3	CACGTA (rc)	119	UPRE-2
TERG_02245	hypothetical protein	TACGTG	55	UPRE-2
TERG_02272	DUF323 domain-containing protein (*T. equinum*)	TACGTG	716	UPRE-2
TERG_02282	hypothetical protein	TACGTG	611	UPRE-2
TERG_02299	HET-C domain-containing protein (*T. tonsurans*)	CACGTA (rc)	977	UPRE-2
TERG_02304	importin subunit beta-5 (*T. equinum*)	CACGTA (rc)	236	UPRE-2
TERG_02317	phosphatidylethanolamine N-methyltransferase	TACGTG	92	UPRE-2
TERG_02327	rRNA biogenesis protein RRP5 (*T. tonsurans*)	TACGTG	9	UPRE-2
TERG_02353	UbiD family decarboxylase, putative (*T. verrucosum*)	CACGTA (rc)	642	UPRE-2

TERG_02355	hypothetical protein	TACGTG	989	UPRE-2
TERG_02356	hypothetical protein	TACGTG	127	UPRE-2
TERG_02368	extracellular developmental signal biosynthesis protein FluG (*A. benhamiae*)	TACGTG	134	UPRE-2
TERG_02371	hypothetical protein	TACGTG	842	UPRE-2
TERG_02376	60S ribosomal protein (*T. equinum*)	CACGTA (rc)	81	UPRE-2
TERG_02392	MYB DNA-binding domain protein (*T. verrucosum*)	CACGTA (rc)	555	UPRE-2
TERG_02405	mating locus protein, putative (*T. verrucosum*)	CACGTA (rc)	904	UPRE-2
TERG_02408	DNA lyase (*T. tonsurans*)	CACGTA (rc)	198	UPRE-2
TERG_02408	DNA lyase (*T. tonsurans*)	CACGTA (rc)	288	UPRE-2
TERG_02481	F-box domain-containing protein (*M. canis*)	CACGTA (rc)	317	UPRE-2
TERG_02497	serine/threonine protein kinase (*T. tonsurans*)	TACGTG	110	UPRE-2
TERG_02538	carboxylesterase (*T. equinum*)	TACGTG	479	UPRE-2
TERG_02556	dynamin GTPase, putative (*T. verrucosum*)	TACGTG	458	UPRE-2
TERG_02572	hypothetical protein	CACGTA (rc)	168	UPRE-2
TERG_02575	hypothetical protein	TACGTG	87	UPRE-2
TERG_02575	hypothetical protein	TACGTG	811	UPRE-2
TERG_02599	dihydrolipoyl dehydrogenase	TACGTG	335	UPRE-2
TERG_02611	WD repeat protein (*A. benhamiae*)	CACGTA (rc)	888	UPRE-2
TERG_02612	DNA-directed RNA polymerase I, II, and III subunit RPABC5	TACGTG	170	UPRE-2
TERG_02645	hypothetical protein	CACGTA (rc)	117	UPRE-2
TERG_02649	benzoate 4-monooxygenase cytochrome P450 (*T. equinum*)	CACGTA (rc)	131	UPRE-2
TERG_02650	NmrA family protein (*T. equinum*)	CACGTA (rc)	443	UPRE-2
TERG_02651	NRPS-like enzyme, putative (*A. benhamiae*)	TACGTG	815	UPRE-2
TERG_02653	hypothetical protein	CACGTA (rc)	576	UPRE-2
TERG_02714	proteinase, putative (*A. benhamiae*)	CACGTA (rc)	49	UPRE-2
TERG_02715	beta-lactamase (*T. equinum*)	TACGTG	736	UPRE-2
TERG_02728	3-hydroxyisobutyrate dehydrogenase	TACGTG	11	UPRE-2
TERG_02732	hypothetical protein	TACGTG	889	UPRE-2
TERG_02753	E3 ubiquitin-protein ligase ubr1 (*T. equinum*)	TACGTG	536	UPRE-2
TERG_02763	ankyrin and HET domain containing protein (*T. equinum*)	CACGTA (rc)	221	UPRE-2
TERG_02769	RNP domain protein (*A. benhamiae*)	TACGTG	879	UPRE-2
TERG_02770	hypothetical protein	TACGTG	88	UPRE-2
TERG_02788	DNA-directed RNA polymerase I (*T. tonsurans*)	TACGTG	603	UPRE-2
TERG_02802	amino acid permease, putative (*T. verrucosum*)	TACGTG	289	UPRE-2

TERG_02803	3-oxoacyl-(acyl-carrier-protein) reductase (*T. verrucosum*)	TACGTG	844	UPRE-2
TERG_02823	hypothetical protein	TACGTG	16	UPRE-2
TERG_02829	hypothetical protein	TACGTG	388	UPRE-2
TERG_02830	hypothetical protein	CACGTA (rc)	645	UPRE-2
TERG_02831	C2H2 finger domain protein, putative (*A. benhamiae*)	CACGTA (rc)	100	UPRE-2
TERG_02848	short chain dehydrogenase/reductase family protein (*A. benhamiae*)	TACGTG	594	UPRE-2
TERG_02849	flavin-containing monooxygenase (*T. equinum*)	CACGTA (rc)	40	UPRE-2
TERG_02854	dynein light chain (*T. tonsurans*)	CACGTA (rc)	42	UPRE-2
TERG_02883	R3H domain protein, putative (*T. verrucosum*)	TACGTG	579	UPRE-2
TERG_02892	hypothetical protein	CACGTA (rc)	423	UPRE-2
TERG_02900	hypothetical protein	CACGTA (rc)	628	UPRE-2
TERG_02901	N-acetyltransferase complex ARD1 subunit (*T. tonsurans*)	TACGTG	729	UPRE-2
TERG_02929	carboxylesterase, putative (*T. verrucosum*)	TACGTG	573	UPRE-2
TERG_02929	carboxylesterase, putative (*T. verrucosum*)	TACGTG	693	UPRE-2
TERG_02972	SRF-type transcription factor rlmA (*T. equinum*)	CACGTA (rc)	73	UPRE-2
TERG_02973	morphogenesis protein (Msb1), putative (*T. verrucosum*)	TACGTG	886	UPRE-2
TERG_02974	hypothetical protein	CACGTA (rc)	962	UPRE-2
TERG_02977	hypothetical protein	CACGTA (rc)	617	UPRE-2
TERG_02978	FAD-dependent oxygenase (*T. tonsurans*)	TACGTG	491	UPRE-2
TERG_02987	mitochondrial import inner membrane translocase subunit tim-17	TACGTG	496	UPRE-2
TERG_03004	3-methyl-2-oxobutanoate hydroxymethyltransferase	CACGTA (rc)	714	UPRE-2
TERG_03009	hypothetical protein	TACGTG	137	UPRE-2
TERG_03010	SIR2 family histone deacetylase (*T. tonsurans*)	TACGTG	482	UPRE-2
TERG_03041	class E vacuolar protein-sorting machinery protein HSE1 (*T. equinum*)	CACGTA (rc)	590	UPRE-2
TERG_03076	centrin-binding protein Sfi1, putative (*T. verrucosum*)	TACGTG	180	UPRE-2
TERG_03078	cytochrome P450 oxidoreductase OrdA-like, putative (*T. verrucosum*)	CACGTA (rc)	448	UPRE-2
TERG_03084	pentatricopeptide repeat protein (*A. benhamiae*)	TACGTG	382	UPRE-2
TERG_03095	methyltransferase, putative (*A. benhamiae*)	TACGTG	523	UPRE-2
TERG_03096	hypothetical protein	TACGTG	955	UPRE-2
TERG_03118	transcriptional corepressor of histone genes (Hir3), putative (*A. benhamiae*)	CACGTA (rc)	73	UPRE-2
TERG_03177	RING finger domain protein, putative (*A. benhamiae*)	CACGTA (rc)	577	UPRE-2
TERG_03179	NlpC/P60-like cell-wall peptidase, putative (*A. benhamiae*)	TACGTG	661	UPRE-2

TERG_03183	hypothetical protein	TACGTG	191	UPRE-2
TERG_03194	nonribosomal peptide synthase, putative (*A. benhamiae*)	CACGTA (rc)	704	UPRE-2
TERG_03203	ELL complex subunit Eap30 (*T. tonsurans*)	CACGTA (rc)	135	UPRE-2
TERG_03206	hsp7-like protein	CACGTA (rc)	88	UPRE-2
TERG_03226	glucosamine-6-phosphate deaminase	CACGTA (rc)	10	UPRE-2
TERG_03234	DUF907 domain-containing protein (*T. equinum*)	TACGTG	181	UPRE-2
TERG_03242	mitochondrial respiratory complex I chaperone (Cia84), putative (*A. benhamiae*)	CACGTA (rc)	250	UPRE-2
TERG_03244	WD repeat containing protein 2 (*T. tonsurans*)	TACGTG	504	UPRE-2
TERG_03248	extracellular metalloproteinase 3	TACGTG	651	UPRE-2
TERG_03250	MFS monocarboxylate transporter (*T. equinum*)	TACGTG	927	UPRE-2
TERG_03258	alkaline phosphatase (*T. tonsurans*)	CACGTA (rc)	605	UPRE-2
TERG_03279	C6 finger domain-containing protein (*T. tonsurans*)	TACGTG	29	UPRE-2
TERG_03296	coatomer subunit zeta (*T. equinum*)	CACGTA (rc)	773	UPRE-2
TERG_03313	fucose-specific lectin FleA (*A. benhamiae*)	TACGTG	915	UPRE-2
TERG_03323	hypothetical protein	TACGTG	421	UPRE-2
TERG_03323	hypothetical protein	TACGTG	427	UPRE-2
TERG_03328	anaphase-promoting complex subunit ApcB (*T. equinum*)	TACGTG	12	UPRE-2
TERG_03328	anaphase-promoting complex subunit ApcB (*T. equinum*)	TACGTG	99	UPRE-2
TERG_03337	hypothetical protein	CACGTA (rc)	165	UPRE-2
TERG_03339	alternative oxidase, mitochondrial	TACGTG	378	UPRE-2
TERG_03350	RecQ family helicase MusN (*T. verrucosum*)	CACGTA (rc)	625	UPRE-2
TERG_03351	kelch repeats protein (*T. equinum*)	TACGTG	962	UPRE-2
TERG_03352	tyrosine decarboxylase (*T. equinum*)	CACGTA (rc)	671	UPRE-2
TERG_03362	KH domain RNA binding protein (*T. tonsurans*)	TACGTG	668	UPRE-2
TERG_03365	hypothetical protein	TACGTG	180	UPRE-2
TERG_03379	AGC/NDR/NDR protein kinase	CACGTA (rc)	892	UPRE-2
TERG_03382	ThiJ/PfpI family protein (*A. benhamiae*)	TACGTG	507	UPRE-2
TERG_03388	hypothetical protein	TACGTG	767	UPRE-2
TERG_03389	orotidine 5'-phosphate decarboxylase	TACGTG	219	UPRE-2
TERG_03391	diacylglycerol O-acyltransferase (*T. equinum*)	CACGTA (rc)	906	UPRE-2
TERG_03406	hypothetical protein	CACGTA (rc)	461	UPRE-2
TERG_03427	peptidyl-prolyl cis-trans isomerase (*T. tonsurans*)	CACGTA (rc)	374	UPRE-2
TERG_03441	hypothetical protein	TACGTG	247	UPRE-2
TERG_03493	hypothetical protein	CACGTA (rc)	444	UPRE-2

TERG_03493	hypothetical protein	CACGTA (rc)	596	UPRE-2
TERG_03495	vesicular-fusion protein SEC17 (*T. equinum*)	CACGTA (rc)	469	UPRE-2
TERG_03501	PtaB protein (*T. tonsurans*)	CACGTA (rc)	771	UPRE-2
TERG_03502	AAA-type ATPase (*T. equinum*)	TACGTG	162	UPRE-2
TERG_03554	hypothetical protein	TACGTG	972	UPRE-2
TERG_03561	short chain dehydrogenase/reductase (*M. canis*)	TACGTG	221	UPRE-2
TERG_03581	DNA mismatch repair protein (*T. tonsurans*)	TACGTG	95	UPRE-2
TERG_03587	WD repeat protein (*T. verrucosum*)	TACGTG	759	UPRE-2
TERG_03603	cytochrome c oxidase assembly protein (*T. tonsurans*)	TACGTG	366	UPRE-2
TERG_03622	hypothetical protein	CACGTA (rc)	64	UPRE-2
TERG_03628	serine/threonine protein kinase (*T. tonsurans*)	TACGTG	395	UPRE-2
TERG_03675	hypothetical protein	CACGTA (rc)	967	UPRE-2
TERG_03690	hypothetical protein	TACGTG	229	UPRE-2
TERG_03704	integral membrane protein Pth11-like, putative (*T. verrucosum*)	CACGTA (rc)	845	UPRE-2
TERG_03711	histone acetyltransferase type B catalytic subunit (*T. equinum*)	CACGTA (rc)	325	UPRE-2
TERG_03729	6-phosphogluconate dehydrogenase (decarboxylating)	CACGTA (rc)	73	UPRE-2
TERG_03731	C6 transcription factor (*T. equinum*)	TACGTG	122	UPRE-2
TERG_03738	60S ribosomal protein L3	TACGTG	37	UPRE-2
TERG_03739	hypothetical protein	TACGTG	522	UPRE-2
TERG_03750	small nuclear ribonucleoprotein (*T. tonsurans*)	CACGTA (rc)	225	UPRE-2
TERG_03752	hypothetical protein	TACGTG	240	UPRE-2
TERG_03772	hypothetical protein	CACGTA (rc)	280	UPRE-2
TERG_03791	NADH-ubiquinone oxidoreductase 20 kDa subunit (*T. equinum*)	TACGTG	896	UPRE-2
TERG_03813	hexose transporter protein (*A. benhamiae*)	TACGTG	233	UPRE-2
TERG_03815	subtilisin-like protease 3	TACGTG	745	UPRE-2
TERG_03850	hypothetical protein	TACGTG	45	UPRE-2
TERG_03877	cytochrome P450 monooxygenase, putative (*T. verrucosum*)	TACGTG	894	UPRE-2
TERG_03879	cell wall biogenesis protein Mhp1, putative (*T. verrucosum*)	CACGTA (rc)	548	UPRE-2
TERG_03884	hypothetical protein	CACGTA (rc)	402	UPRE-2
TERG_03885	hypothetical protein	TACGTG	298	UPRE-2
TERG_03903	DUF6 domain-containing protein (*T. equinum*)	CACGTA (rc)	594	UPRE-2
TERG_03904	aflatoxin B1 aldehyde reductase member 2 (*T. tonsurans*)	TACGTG	27	UPRE-2
TERG_03907	neutral amino acid permease (*T. tonsurans*)	TACGTG	42	UPRE-2

TERG_03927	leucine permease transcriptional regulator (SAC3), putative (*A. benhamiae*)	CACGTA (rc)	531	UPRE-2
TERG_03967	THO complex protein subunit 2 (*T. equinum*)	CACGTA (rc)	114	UPRE-2
TERG_03970	DUF636 domain-containing protein (*T. equinum*)	TACGTG	540	UPRE-2
TERG_04035	hypothetical protein	CACGTA (rc)	705	UPRE-2
TERG_04052	hypothetical protein	TACGTG	173	UPRE-2
TERG_04054	hypothetical protein	TACGTG	268	UPRE-2
TERG_04061	ribosome biogenesis GTPase Lsg1 (*T. tonsurans*)	TACGTG	257	UPRE-2
TERG_04063	CDP-diacylglycerol-glycerol-3-phosphate 3-phosphatidyltransferase (*T. tonsurans*)	CACGTA (rc)	526	UPRE-2
TERG_04064	NADH-ubiquinone oxidoreductase subunit (*T. tonsurans*)	TACGTG	690	UPRE-2
TERG_04068	cytochrome P450 monooxygenase, putative (*A. benhamiae*)	TACGTG	542	UPRE-2
TERG_04072	SNF2 family helicase/ATPase, putative (*T. verrucosum*)	CACGTA (rc)	465	UPRE-2
TERG_04073	glutathione synthetase	TACGTG	255	UPRE-2
TERG_04080	transcription elongation factor spt4	CACGTA (rc)	871	UPRE-2
TERG_04081	serine/threonine protein phosphatase PPT1 (*T. tonsurans*)	TACGTG	726	UPRE-2
TERG_04087	DnaJ domain-containing protein (*T. tonsurans*)	TACGTG	42	UPRE-2
TERG_04131	heat shock trehalose synthase, putative (*T. verrucosum*)	TACGTG	894	UPRE-2
TERG_04139	hypothetical protein	CACGTA (rc)	729	UPRE-2
TERG_04142	cofilin (*T. equinum*)	TACGTG	891	UPRE-2
TERG_04146	FAD/FMN-containing isoamyl alcohol oxidase MreA-like, putative (*A. benhamiae*)	TACGTG	399	UPRE-2
TERG_04160	hypothetical protein	CACGTA (rc)	608	UPRE-2
TERG_04160	hypothetical protein	CACGTA (rc)	908	UPRE-2
TERG_04182	MFS multidrug transporter (*T. tonsurans*)	CACGTA (rc)	418	UPRE-2
TERG_04204	hypothetical protein	CACGTA (rc)	801	UPRE-2
TERG_04235	WW domain-containing protein (*T. tonsurans*)	TACGTG	64	UPRE-2
TERG_04236	ankyrin repeat protein (*T. tonsurans*)	TACGTG	801	UPRE-2
TERG_04255	hypothetical protein	TACGTG	963	UPRE-2
TERG_04265	palmitoyltransferase pfa5 (*T. equinum*)	TACGTG	365	UPRE-2
TERG_04283	hypothetical protein	TACGTG	760	UPRE-2
TERG_04324	extracellular metalloproteinase 4	CACGTA (rc)	160	UPRE-2
TERG_04324	extracellular metalloproteinase 4	CACGTA (rc)	583	UPRE-2
TERG_04370	hypothetical protein	CACGTA (rc)	502	UPRE-2
TERG_04371	hypothetical protein	TACGTG	468	UPRE-2

TERG_04374	STE/STE20/YSK protein kinase	CACGTA (rc)	118	UPRE-2
TERG_04416	penicillolysin/deuterolysin metalloprotease, putative (*A. benhamiae*)	TACGTG	47	UPRE-2
TERG_04421	hypothetical protein	TACGTG	782	UPRE-2
TERG_04437	nucleolar protein NOP2 (*T. tonsurans*)	TACGTG	627	UPRE-2
TERG_04438	GDP-mannose transporter 1	TACGTG	973	UPRE-2
TERG_04443	lipase/esterase, putative (*A. benhamiae*)	CACGTA (rc)	565	UPRE-2
TERG_04452	cell cycle control protein (*T. tonsurans*)	CACGTA (rc)	160	UPRE-2
TERG_04457	POS9-activating factor FAP7 (*T. tonsurans*)	TACGTG	95	UPRE-2
TERG_04458	RdgB/HAM1 family non-canonical purine NTP pyrophosphatase	CACGTA (rc)	470	UPRE-2
TERG_04459	class V myosin (*T. tonsurans*)	CACGTA (rc)	974	UPRE-2
TERG_04464	hypothetical protein	CACGTA (rc)	491	UPRE-2
TERG_04502	impact family protein (*T. equinum*)	TACGTG	690	UPRE-2
TERG_04506	60S ribosomal protein L4-A (*T. tonsurans*)	TACGTG	929	UPRE-2
TERG_04508	AAA ATPase (*T. equinum*)	TACGTG	422	UPRE-2
TERG_04515	WD domain-containing protein (*T. tonsurans*)	TACGTG	852	UPRE-2
TERG_04553	thymidylate kinase	CACGTA (rc)	544	UPRE-2
TERG_04576	CRAL/TRIO domain protein (*T. verrucosum*)	CACGTA (rc)	859	UPRE-2
TERG_04616	hypothetical protein	TACGTG	432	UPRE-2
TERG_04666	hypothetical protein	TACGTG	682	UPRE-2
TERG_04713	cystathionine beta-synthase (*T. tonsurans*)	CACGTA (rc)	405	UPRE-2
TERG_04739	3-ketoacyl-CoA reductase	CACGTA (rc)	373	UPRE-2
TERG_04746	nucleolar GTP-binding protein (*T. tonsurans*)	TACGTG	373	UPRE-2
TERG_04810	bifunctional fatty acid transporter/acyl-CoA synthetase (FAT1), putative (*A. benhamiae*)	TACGTG	251	UPRE-2
TERG_04828	IZH family channel protein (*T. tonsurans*)	CACGTA (rc)	377	UPRE-2
TERG_04828	IZH family channel protein (*T. tonsurans*)	CACGTA (rc)	538	UPRE-2
TERG_04881	hypothetical protein	CACGTA (rc)	773	UPRE-2
TERG_04891	ubiquitin-protein ligase E3C (*T. equinum*)	TACGTG	828	UPRE-2
TERG_04896	GARP complex subunit (Sac2), putative (*T. verrucosum*)	CACGTA (rc)	352	UPRE-2
TERG_04923	dynactin (*T. equinum*)	TACGTG	299	UPRE-2
TERG_04931	allantoate permease (*T. equinum*)	CACGTA (rc)	189	UPRE-2
TERG_04938	hypothetical protein	TACGTG	984	UPRE-2
TERG_04954	hypothetical protein	TACGTG	13	UPRE-2
TERG_04960	glutathione S-transferase Ure2-like, putative (*A. benhamiae*)	TACGTG	298	UPRE-2
TERG_04960	glutathione S-transferase Ure2-like, putative (*A. benhamiae*)	TACGTG	679	UPRE-2

TERG_04980	RNA exonuclease, putative (*A. benhamiae*)	CACGTA (rc)	6	UPRE-2
TERG_04980	RNA exonuclease, putative (*A. benhamiae*)	CACGTA (rc)	392	UPRE-2
TERG_04985	ATP synthase subunit F (*T. tonsurans*)	CACGTA (rc)	889	UPRE-2
TERG_04986	NADH-ubiquinone oxidoreductase 18 kDa subunit (*T. tonsurans*)	TACGTG	941	UPRE-2
TERG_05024	phosphomevalonate kinase (*T. tonsurans*)	TACGTG	500	UPRE-2
TERG_05026	hypothetical protein	TACGTG	276	UPRE-2
TERG_05057	DUF775 domain-containing protein (*T. equinum*)	CACGTA (rc)	14	UPRE-2
TERG_05077	D-amino-acid oxidase (*T. equinum*)	CACGTA (rc)	816	UPRE-2
TERG_05083	adaptin ear-binding coat-associated protein 1 (*M. canis*)	CACGTA (rc)	120	UPRE-2
TERG_05174	acetyltransferase, GNAT family (*T. verrucosum*)	CACGTA (rc)	669	UPRE-2
TERG_05185	RING finger ubiquitin ligase (*T. tonsurans*)	CACGTA (rc)	617	UPRE-2
TERG_05208	transcription factor iws1	CACGTA (rc)	208	UPRE-2
TERG_05263	CCCH zinc finger DNA binding protein (*A. benhamiae*)	CACGTA (rc)	873	UPRE-2
TERG_05264	signal sequence receptor alpha chain (*T. equinum*)	TACGTG	852	UPRE-2
TERG_05265	DEAD/DEAH box helicase (*T. tonsurans*)	CACGTA (rc)	116	UPRE-2
TERG_05292	hypothetical protein	TACGTG	706	UPRE-2
TERG_05294	hypothetical protein	CACGTA (rc)	825	UPRE-2
TERG_05295	TFIIIC transcription initiation factor complex subunits Tfc3, putative (*A. benhamiae*)	TACGTG	689	UPRE-2
TERG_05318	polyphosphoinositide phosphatase (*T. tonsurans*)	TACGTG	242	UPRE-2
TERG_05328	pre-rRNA-processing protein ESF2 (*T. tonsurans*)	TACGTG	148	UPRE-2
TERG_05328	pre-rRNA-processing protein ESF2 (*T. tonsurans*)	TACGTG	202	UPRE-2
TERG_05333	DDT domain-containing protein (*T. tonsurans*)	CACGTA (rc)	329	UPRE-2
TERG_05347	epoxide hydrolase, putative (*T. verrucosum*)	CACGTA (rc)	84	UPRE-2
TERG_05363	hypothetical protein	TACGTG	98	UPRE-2
TERG_05440	hypothetical protein	CACGTA (rc)	289	UPRE-2
TERG_05441	cytochrome P450 alkane hydroxylase, putative (*T. verrucosum*)	TACGTG	908	UPRE-2
TERG_05483	transcription elongation factor spt5 (*M. gypseum*)	CACGTA (rc)	53	UPRE-2
TERG_05510	DUF858 domain protein (*T. verrucosum*)	TACGTG	516	UPRE-2
TERG_05529	telomere length regulator protein (Rif1), putative (*A. benhamiae*)	CACGTA (rc)	798	UPRE-2
TERG_05532	monosaccharide transporter (*T. tonsurans*)	TACGTG	644	UPRE-2
TERG_05534	hypothetical protein	CACGTA (rc)	190	UPRE-2
TERG_05537	MFS multidrug transporter (*T. equinum*)	CACGTA (rc)	801	UPRE-2
TERG_05554	integral membrane protein (*T. equinum*)	CACGTA (rc)	306	UPRE-2
TERG_05588	NRPS-like enzyme, putative (*A. benhamiae*)	TACGTG	908	UPRE-2

TERG_05605	Hrq1p (*M. gypseum*)	CACGTA (rc)	672	UPRE-2
TERG_05606	hypothetical protein	TACGTG	831	UPRE-2
TERG_05615	hsp70-like protein (*T. equinum*)	CACGTA (rc)	271	UPRE-2
TERG_05634	mitochondrial protein (*T. tonsurans*)	CACGTA (rc)	68	UPRE-2
TERG_05634	mitochondrial protein (*T. tonsurans*)	CACGTA (rc)	76	UPRE-2
TERG_05637	4'-phosphopantetheinyl transferase superfamily protein (*T. tonsurans*)	TACGTG	599	UPRE-2
TERG_05644	mucin family signaling protein Msb2, putative (*T. verrucosum*)	CACGTA (rc)	78	UPRE-2
TERG_05645	vacuolar protein sorting protein Vps36 (*T. tonsurans*)	CACGTA (rc)	428	UPRE-2
TERG_05681	actin (*T. tonsurans*)	CACGTA (rc)	137	UPRE-2
TERG_05708	decapping enzyme Dcp1 (*T. tonsurans*)	TACGTG	70	UPRE-2
TERG_05716	PAP/25A associated domain-containing protein (*T. equinum*)	TACGTG	675	UPRE-2
TERG_05767	dynein light chain (*T. equinum*)	CACGTA (rc)	174	UPRE-2
TERG_05771	hypothetical protein	CACGTA (rc)	358	UPRE-2
TERG_05799	chlorophyll synthesis pathway protein BchC	CACGTA (rc)	687	UPRE-2
TERG_05800	ubiquitin-protein ligase (Hul4), putative (*A. benhamiae*)	CACGTA (rc)	425	UPRE-2
TERG_05806	methionine aminopeptidase, type I	CACGTA (rc)	200	UPRE-2
TERG_05807	kynurenine 3-monooxygenase (*T. tonsurans*)	TACGTG	226	UPRE-2
TERG_05817	hypothetical protein	TACGTG	172	UPRE-2
TERG_05836	hypothetical protein	TACGTG	294	UPRE-2
TERG_05840	hypothetical protein	CACGTA (rc)	740	UPRE-2
TERG_05841	AmmeMemoRadiSam system protein B	TACGTG	298	UPRE-2
TERG_05897	CoA-binding protein (*T. equinum*)	CACGTA (rc)	126	UPRE-2
TERG_05906	serine/threonine protein kinase	CACGTA (rc)	167	UPRE-2
TERG_05917	MOSC domain protein (*A. benhamiae*)	CACGTA (rc)	18	UPRE-2
TERG_05924	transcription factor TFIIIB complex subunit brf1 (*M. gypseum*)	TACGTG	217	UPRE-2
TERG_05938	tetratricopeptide repeat domain-containing protein (*T. equinum*)	TACGTG	139	UPRE-2
TERG_05962	phosphotransferase (*M. canis*)	TACGTG	58	UPRE-2
TERG_05964	hypothetical protein	TACGTG	449	UPRE-2
TERG_05991	hypothetical protein	CACGTA (rc)	44	UPRE-2
TERG_06019	hypothetical protein	TACGTG	502	UPRE-2
TERG_06031	meiotic recombination protein DLH1	TACGTG	50	UPRE-2
TERG_06081	hypothetical protein	TACGTG	424	UPRE-2
TERG_06128	phosphoribosylglycinamide formyltransferase	CACGTA (rc)	261	UPRE-2
TERG_06155	phosphotransferase (*T. tonsurans*)	CACGTA (rc)	851	UPRE-2

TERG_06180	endonuclease/exonuclease/phosphatase family protein (*T. verrucosum*)	TACGTG	700	UPRE-2
TERG_06249	hypothetical protein	CACGTA (rc)	256	UPRE-2
TERG_06266	hypothetical protein	TACGTG	326	UPRE-2
TERG_06322	alpha-1,2-mannosyltransferase (*T. tonsurans*)	CACGTA (rc)	181	UPRE-2
TERG_06367	SH3 domain protein, putative (*A. benhamiae*)	TACGTG	867	UPRE-2
TERG_06414	DUF1338 domain-containing protein (*T. equinum*)	TACGTG	422	UPRE-2
TERG_06449	CCAAT-binding factor complex subunit HapE (*T. tonsurans*)	CACGTA (rc)	984	UPRE-2
TERG_06465	O-mannosyl transferase pmtA-Aspergillus niger (*T. equinum*)	TACGTG	594	UPRE-2
TERG_06492	hypothetical protein	TACGTG	285	UPRE-2
TERG_06500	mitochondrial GTP/GDP carrier protein 1	CACGTA (rc)	613	UPRE-2
TERG_06501	hypothetical protein	TACGTG	605	UPRE-2
TERG_06510	hypothetical protein	CACGTA (rc)	108	UPRE-2
TERG_06532	chitin synthase export chaperone (*M. gypseum*)	TACGTG	242	UPRE-2
TERG_06536	tRNA-specific adenosine-34 deaminase subunit Tad3 (*T. equinum*)	CACGTA (rc)	71	UPRE-2
TERG_06540	glutathione transferase (*T. tonsurans*)	CACGTA (rc)	288	UPRE-2
TERG_06541	hypothetical protein	TACGTG	922	UPRE-2
TERG_06565	protein kinase subdomain-containing protein (*T. equinum*)	CACGTA (rc)	399	UPRE-2
TERG_06584	hypothetical protein	TACGTG	425	UPRE-2
TERG_06590	ABC1 (*T. equinum*)	CACGTA (rc)	81	UPRE-2
TERG_06591	urease accessory protein UreF (*T. tonsurans*)	TACGTG	650	UPRE-2
TERG_06607	serine-rich protein, putative (*T. verrucosum*)	CACGTA (rc)	913	UPRE-2
TERG_06630	proteasome component PRE5 (*T. equinum*)	CACGTA (rc)	210	UPRE-2
TERG_06653	conserved serine-rich protein (*T. verrucosum*)	CACGTA (rc)	938	UPRE-2
TERG_06683	hypothetical protein	CACGTA (rc)	490	UPRE-2
TERG_06684	molybdenum cofactor biosynthetic protein (*T. equinum*)	TACGTG	964	UPRE-2
TERG_06714	hypothetical protein	CACGTA (rc)	980	UPRE-2
TERG_06715	LEA domain protein (*A. benhamiae*)	TACGTG	283	UPRE-2
TERG_06778	PAP2 domain protein (*T. verrucosum*)	CACGTA (rc)	573	UPRE-2
TERG_06785	cytochrome P450 monooxygenase (*T. equinum*)	TACGTG	489	UPRE-2
TERG_06798	hypothetical protein	TACGTG	649	UPRE-2
TERG_06801	cyclic nucleotide-binding domain-containing protein (*T. tonsurans*)	TACGTG	778	UPRE-2
TERG_06833	hypothetical protein	TACGTG	294	UPRE-2
TERG_06844	40S ribosomal protein S21	CACGTA (rc)	47	UPRE-2

TERG_06859	exosome complex endonuclease 1/ribosomal RNA processing protein (*T. equinum*)	CACGTA (rc)	395	UPRE-2
TERG_06872	hypothetical protein	CACGTA (rc)	272	UPRE-2
TERG_06872	hypothetical protein	CACGTA (rc)	437	UPRE-2
TERG_06884	C6 transcription factor (War1), putative (*T. verrucosum*)	CACGTA (rc)	308	UPRE-2
TERG_06890	DUF636 domain protein (*T. verrucosum*)	CACGTA (rc)	474	UPRE-2
TERG_06891	C6 transcription factor, putative (*T. verrucosum*)	TACGTG	808	UPRE-2
TERG_06926	chitosanase (*T. equinum*)	CACGTA (rc)	531	UPRE-2
TERG_06929	chitinase (*T. equinum*)	CACGTA (rc)	822	UPRE-2
TERG_06955	hypothetical protein	CACGTA (rc)	548	UPRE-2
TERG_06956	RNA polymerase I-specific transcription initiation factor rrn3 (*M. gypseum*)	TACGTG	246	UPRE-2
TERG_06963	hsp90-like protein	TACGTG	804	UPRE-2
TERG_06971	hypothetical protein	CACGTA (rc)	508	UPRE-2
TERG_06980	hypothetical protein	CACGTA (rc)	291	UPRE-2
TERG_07012	rRNA 2'-O-methyltransferase fibrillarin	TACGTG	457	UPRE-2
TERG_07033	glycerophosphodiesterase GDE1 (*T. equinum*)	TACGTG	17	UPRE-2
TERG_07035	hypothetical protein	TACGTG	690	UPRE-2
TERG_07057	Hsp70 family chaperone, putative (*A. benhamiae*)	TACGTG	619	UPRE-2
TERG_07060	acyl-CoA thioesterase II	CACGTA (rc)	148	UPRE-2
TERG_07062	Ser/Thr protein phosphatase family protein (*T. verrucosum*)	TACGTG	839	UPRE-2
TERG_07062	Ser/Thr protein phosphatase family protein (*T. verrucosum*)	TACGTG	899	UPRE-2
TERG_07085	peptidase (*T. tonsurans*)	CACGTA (rc)	336	UPRE-2
TERG_07109	phosphotransferase enzyme family protein (*T. equinum*)	CACGTA (rc)	416	UPRE-2
TERG_07110	kelch domain-containing protein domain-containing protein (*T. tonsurans*)	TACGTG	949	UPRE-2
TERG_07158	cell cycle regulatory protein, putative (*T. verrucosum*)	CACGTA (rc)	307	UPRE-2
TERG_07159	geranylgeranyl transferae type II alpha subunit, putative (*T. verrucosum*)	CACGTA (rc)	440	UPRE-2
TERG_07162	geranylgeranyl transferae type II alpha subunit, putative (*T. verrucosum*)	TACGTG	640	UPRE-2
TERG_07170	MFS transporter, putative (*A. benhamiae*)	CACGTA (rc)	625	UPRE-2
TERG_07171	hypothetical protein	TACGTG	531	UPRE-2
TERG_07190	hypothetical protein	CACGTA (rc)	714	UPRE-2
TERG_07201	hypothetical protein	TACGTG	579	UPRE-2
TERG_07216	hypothetical protein	CACGTA (rc)	412	UPRE-2
TERG_07217	histone acetyltransferase, putative (*T. verrucosum*)	TACGTG	868	UPRE-2
TERG_07221	hypothetical protein	TACGTG	808	UPRE-2

TERG_07227	proteasome activator subunit 4 (*T. tonsurans*)	TACGTG	626	UPRE-2
TERG_07229	vacuolar ATP synthase 116kDa subunit (*T. tonsurans*)	TACGTG	425	UPRE-2
TERG_07269	mannose-1-phosphate guanyltransferase	CACGTA (rc)	318	UPRE-2
TERG_07274	hypothetical protein	CACGTA (rc)	935	UPRE-2
TERG_07284	hypothetical protein	TACGTG	283	UPRE-2
TERG_07290	glutathione transferase (*T. equinum*)	CACGTA (rc)	109	UPRE-2
TERG_07299	O-methyltransferase (*M. gypseum*)	CACGTA (rc)	327	UPRE-2
TERG_07323	endosomal peripheral membrane protein (*T. equinum*)	CACGTA (rc)	971	UPRE-2
TERG_07324	ubiquitin-conjugating enzyme (*T. equinum*)	TACGTG	569	UPRE-2
TERG_07339	WD repeat-containing protein (*T. tonsurans*)	CACGTA (rc)	324	UPRE-2
TERG_07351	hypothetical protein	CACGTA (rc)	765	UPRE-2
TERG_07395	WD repeat protein (*T. tonsurans*)	CACGTA (rc)	114	UPRE-2
TERG_07396	DNA-directed RNA polymerase II subunit RPB1 (*M. gypseum*)	TACGTG	509	UPRE-2
TERG_07398	tRNA(His) guanylyltransferase (*T. tonsurans*)	CACGTA (rc)	889	UPRE-2
TERG_07399	fibronectin type III domain-containing protein (*M. gypseum*)	CACGTA (rc)	194	UPRE-2
TERG_07399	fibronectin type III domain-containing protein (*M. gypseum*)	CACGTA (rc)	804	UPRE-2
TERG_07400	nuclear elongation and deformation protein 1 (*T. equinum*)	TACGTG	149	UPRE-2
TERG_07416	peroxisome targeting signal receptor Pex7 (*T. tonsurans*)	CACGTA (rc)	479	UPRE-2
TERG_07437	class II aldolase/adducin domain-containing protein (*T. tonsurans*)	CACGTA (rc)	762	UPRE-2
TERG_07441	methyltransferase (*T. tonsurans*)	TACGTG	869	UPRE-2
TERG_07443	hypothetical protein	TACGTG	267	UPRE-2
TERG_07443	hypothetical protein	TACGTG	820	UPRE-2
TERG_07468	hypothetical protein	CACGTA (rc)	593	UPRE-2
TERG_07482	ribonuclease H1, putative (*A. benhamiae*)	CACGTA (rc)	972	UPRE-2
TERG_07499	CCCH zinc finger and SMR domain-containing protein (*M. gypseum*)	CACGTA (rc)	286	UPRE-2
TERG_07501	nitrate reductase (*T. tonsurans*)	TACGTG	617	UPRE-2
TERG_07509	serine/threonine protein kinase (*M. canis*)	CACGTA (rc)	596	UPRE-2
TERG_07510	hypothetical protein	CACGTA (rc)	28	UPRE-2
TERG_07530	50S ribosomal protein L36 (*T. tonsurans*)	CACGTA (rc)	538	UPRE-2
TERG_07533	hypothetical protein	CACGTA (rc)	724	UPRE-2
TERG_07534	NADH-ubiquinone oxidoreductase complex 1/LYR family protein (*M. gypseum*)	TACGTG	148	UPRE-2
TERG_07596	hypothetical protein	CACGTA (rc)	909	UPRE-2

TERG_07607	hypothetical protein	TACGTG	412	UPRE-2
TERG_07612	hypothetical protein	TACGTG	232	UPRE-2
TERG_07628	tyrosine phosphatase family protein (*A. benhamiae*)	CACGTA (rc)	211	UPRE-2
TERG_07653	DNA repair protein (Rad57), putative (*T. verrucosum*)	TACGTG	306	UPRE-2
TERG_07656	lupus La protein (*T. equinum*)	CACGTA (rc)	529	UPRE-2
TERG_07657	chitin synthase class VI (*T. equinum*)	TACGTG	568	UPRE-2
TERG_07672	GABA permease (*T. equinum*)	TACGTG	377	UPRE-2
TERG_07689	ribosomal biogenesis protein Gar2 (*T. tonsurans*)	CACGTA (rc)	639	UPRE-2
TERG_07706	hypothetical protein	CACGTA (rc)	827	UPRE-2
TERG_07708	hypothetical protein	CACGTA (rc)	404	UPRE-2
TERG_07720	copper-transporting ATPase (*T. equinum*)	TACGTG	789	UPRE-2
TERG_07731	2Fe-2S iron-sulfur cluster binding domain-containing protein (*T. tonsurans*)	CACGTA (rc)	221	UPRE-2
TERG_07731	2Fe-2S iron-sulfur cluster binding domain-containing protein (*T. tonsurans*)	CACGTA (rc)	580	UPRE-2
TERG_07740	farnesyl pyrophosphate synthetase (*T. equinum*)	CACGTA (rc)	690	UPRE-2
TERG_07755	ergot alkaloid biosynthetic protein A (*T. verrucosum*)	TACGTG	642	UPRE-2
TERG_07774	EF hand domain-containing protein (*T. tonsurans*)	TACGTG	426	UPRE-2
TERG_07794	hypothetical protein	TACGTG	509	UPRE-2
TERG_07831	GNAT family acetyltransferase, putative (*T. verrucosum*)	TACGTG	490	UPRE-2
TERG_07844	HAL protein kinase	CACGTA (rc)	30	UPRE-2
TERG_07858	aldo/keto reductase (*T. equinum*)	CACGTA (rc)	823	UPRE-2
TERG_07859	ubiquitin C-terminal hydrolase L3 (*T. tonsurans*)	TACGTG	163	UPRE-2
TERG_07864	SVP1-like protein (*T. tonsurans*)	CACGTA (rc)	105	UPRE-2
TERG_07881	pre-mRNA-splicing factor isy1	CACGTA (rc)	922	UPRE-2
TERG_07906	hypothetical protein	CACGTA (rc)	769	UPRE-2
TERG_07962	hypothetical protein	TACGTG	321	UPRE-2
TERG_07993	hypothetical protein	TACGTG	243	UPRE-2
TERG_07998	hypothetical protein	CACGTA (rc)	12	UPRE-2
TERG_08003	fungal specific transcription factor domain-containing protein (*T. equinum*)	CACGTA (rc)	307	UPRE-2
TERG_08013	Rox3 mediator complex subunit (*T. equinum*)	CACGTA (rc)	238	UPRE-2
TERG_08036	mitochondrial carrier protein (*T. equinum*)	CACGTA (rc)	105	UPRE-2
TERG_08064	choline kinase, putative (*A. benhamiae*)	CACGTA (rc)	673	UPRE-2
TERG_08097	hypothetical protein	TACGTG	123	UPRE-2
TERG_08102	3-isopropylmalate dehydrogenase A	TACGTG	554	UPRE-2
TERG_08107	phosphoglycerate mutase (*T. tonsurans*)	TACGTG	238	UPRE-2
TERG_08148	hypothetical protein	TACGTG	10	UPRE-2

TERG_08182	hypothetical protein	CACGTA (rc)	766	UPRE-2
TERG_08184	dioxygenase, putative (*T. verrucosum*)	TACGTG	464	UPRE-2
TERG_08191	glucooligosaccharide oxidase (*T. equinum*)	CACGTA (rc)	75	UPRE-2
TERG_08194	serine/threonine protein kinase (*M. canis*)	TACGTG	887	UPRE-2
TERG_08245	branched-chain amino acid aminotransferase (*T. tonsurans*)	TACGTG	767	UPRE-2
TERG_08260	subtilisin-like protease 2	TACGTG	311	UPRE-2
TERG_08264	L-xylulose reductase	TACGTG	711	UPRE-2
TERG_08266	hypothetical protein	TACGTG	40	UPRE-2
TERG_08277	transcriptional elongation regulator Elc1/Elongin C (*T. tonsurans*)	CACGTA (rc)	369	UPRE-2
TERG_08289	Rpp14 family protein (*T. equinum*)	TACGTG	267	UPRE-2
TERG_08295	benzoate 4-monooxygenase cytochrome P450 (*M. canis*)	CACGTA (rc)	149	UPRE-2
TERG_08298	RTA1 domain-containing protein (*T. equinum*)	CACGTA (rc)	549	UPRE-2
TERG_08306	hypothetical protein	CACGTA (rc)	658	UPRE-2
TERG_08307	ubiquitin-protein ligase (*T. tonsurans*)	TACGTG	828	UPRE-2
TERG_08316	aflatoxin efflux pump (*M. gypseum*)	TACGTG	820	UPRE-2
TERG_08324	hypothetical protein	CACGTA (rc)	526	UPRE-2
TERG_08368	TTK protein kinase	CACGTA (rc)	474	UPRE-2
TERG_08401	protein transport membrane glycoprotein Sec20, putative (*T. verrucosum*)	CACGTA (rc)	719	UPRE-2
TERG_08417	Hsc70 cochaperone (*T. tonsurans*)	TACGTG	156	UPRE-2
TERG_08444	CipC protein (*M. canis*)	CACGTA (rc)	154	UPRE-2
TERG_08460	microfibril-associated protein (*T. equinum*)	TACGTG	858	UPRE-2
TERG_08462	NAD kinase/ATP NAD kinase (*T. tonsurans*)	CACGTA (rc)	308	UPRE-2
TERG_08462	NAD kinase/ATP NAD kinase (*T. tonsurans*)	CACGTA (rc)	648	UPRE-2
TERG_08463	hypothetical protein	TACGTG	230	UPRE-2
TERG_08463	hypothetical protein	TACGTG	570	UPRE-2
TERG_08468	hydroxyacylglutathione hydrolase	TACGTG	654	UPRE-2
TERG_08474	nuclear protein SNF4 (*T. tonsurans*)	CACGTA (rc)	149	UPRE-2
TERG_08478	translation initiation factor eif-2b delta subunit (*T. tonsurans*)	CACGTA (rc)	892	UPRE-2
TERG_08479	kinesin family protein (*T. verrucosum*)	CACGTA (rc)	569	UPRE-2
TERG_08508	hypothetical protein	TACGTG	137	UPRE-2
TERG_08524	C2H2 finger domain-containing protein (*T. tonsurans*)	CACGTA (rc)	26	UPRE-2
TERG_08526	N2,N2-dimethylguanosine tRNA methyltransferase	TACGTG	217	UPRE-2
TERG_08539	Diacylglycerol kinase domain-containing protein (*T. equinum*)	TACGTG	142	UPRE-2

TERG_08539	Diacylglycerol kinase domain-containing protein (*T. equinum*)	TACGTG	433	UPRE-2
TERG_08539	Diacylglycerol kinase domain-containing protein (*T. equinum*)	TACGTG	851	UPRE-2
TERG_08550	tRNA-specific adenosine deaminase subunit Tad2p/ADAT2 (*T. tonsurans*)	TACGTG	977	UPRE-2
TERG_08593	seven transmembrane protein 1 (*T. equinum*)	TACGTG	744	UPRE-2
TERG_08601	hypothetical protein	TACGTG	865	UPRE-2
TERG_08602	alpha-ketoglutarate-dependent taurine dioxygenase (*T. verrucosum*)	TACGTG	519	UPRE-2
TERG_08620	siderophore iron transporter (*T. equinum*)	CACGTA (rc)	895	UPRE-2
TERG_08622	transferase (*M. canis*)	TACGTG	371	UPRE-2
TERG_08697	serine/threonine protein kinase	CACGTA (rc)	829	UPRE-2
TERG_08724	hypothetical protein	TACGTG	343	UPRE-2
TERG_08803	hypothetical protein	TACGTG	308	UPRE-2
TERG_08823	hypothetical protein	CACGTA (rc)	678	UPRE-2
TERG_08869	hypothetical protein	TACGTG	711	UPRE-2
TERG_08924	MIZ zinc finger domain protein (*A. benhamiae*)	CACGTA (rc)	346	UPRE-2
TERG_08979	CMGC/SRPK protein kinase	TACGTG	577	UPRE-2
TERG_08990	hypothetical protein	CACGTA (rc)	33	UPRE-2
TERG_08990	hypothetical protein	CACGTA (rc)	41	UPRE-2
TERG_11513	esterase (*T. equinum*)	TACGTG	46	UPRE-2
TERG_11516	hypothetical protein	TACGTG	571	UPRE-2
TERG_11560	sterol O-acyltransferase (*T. tonsurans*)	TACGTG	479	UPRE-2
TERG_11573	hypothetical protein	TACGTG	484	UPRE-2
TERG_11574	hypothetical protein	CACGTA (rc)	749	UPRE-2
TERG_11589	hypothetical protein	CACGTA (rc)	727	UPRE-2
TERG_11593	carboxypeptidase Y, putative (*A. benhamiae*)	TACGTG	695	UPRE-2
TERG_11614	hypothetical protein	TACGTG	765	UPRE-2
TERG_11615	hypothetical protein	TACGTG	620	UPRE-2
TERG_11625	allantoicase (*T. equinum*)	TACGTG	904	UPRE-2
TERG_11649	hypothetical protein	TACGTG	537	UPRE-2
TERG_11653	calcofluor white hypersensitive protein (*T. equinum*)	TACGTG	446	UPRE-2
TERG_11676	hypothetical protein	TACGTG	682	UPRE-2
TERG_11694	hypothetical protein	TACGTG	83	UPRE-2
TERG_11701	hypothetical protein	CACGTA (rc)	123	UPRE-2
TERG_11713	vesicle-associated membrane protein 712 (*T. tonsurans*)	TACGTG	411	UPRE-2
TERG_11719	hypothetical protein	TACGTG	232	UPRE-2
TERG_11729	hypothetical protein	TACGTG	709	UPRE-2

TERG_11748	hypothetical protein	TACGTG	816	UPRE-2
TERG_11749	hypothetical protein	TACGTG	524	UPRE-2
TERG_11751	PCI domain-containing protein (*T. tonsurans*)	TACGTG	463	UPRE-2
TERG_11752	hypothetical protein	TACGTG	63	UPRE-2
TERG_11759	hypothetical protein	CACGTA (rc)	570	UPRE-2
TERG_11795	hypothetical protein	TACGTG	21	UPRE-2
TERG_11804	hypothetical protein	CACGTA (rc)	285	UPRE-2
TERG_11809	hydroxymethylglutaryl-CoA synthase	CACGTA (rc)	427	UPRE-2
TERG_11813	FAD dependent oxidoreductase, putative (*A. benhamiae*)	TACGTG	84	UPRE-2
TERG_11855	hypothetical protein	TACGTG	27	UPRE-2
TERG_11880	hypothetical protein	TACGTG	649	UPRE-2
TERG_11898	hypothetical protein	CACGTA (rc)	833	UPRE-2
TERG_11919	extracellular lipase (*T. tonsurans*)	CACGTA (rc)	448	UPRE-2
TERG_11919	extracellular lipase (*T. tonsurans*)	CACGTA (rc)	568	UPRE-2
TERG_11920	vesicle-mediated transport protein Bfr2/Che-1, putative (*T. verrucosum*)	TACGTG	909	UPRE-2
TERG_11922	myo-inositol-1(or 4)-monophosphatase (*T. verrucosum*)	TACGTG	593	UPRE-2
TERG_11936	sister chromatid separation protein (*T. tonsurans*)	CACGTA (rc)	948	UPRE-2
TERG_11938	carboxylesterase, putative (*A. benhamiae*)	TACGTG	429	UPRE-2
TERG_11939	hypothetical protein	TACGTG	11	UPRE-2
TERG_11942	MFS maltose permease (*T. tonsurans*)	CACGTA (rc)	621	UPRE-2
TERG_11943	MFS maltose permease (*T. tonsurans*)	CACGTA (rc)	567	UPRE-2
TERG_11946	hypothetical protein	TACGTG	976	UPRE-2
TERG_11963	hypothetical protein	TACGTG	740	UPRE-2
TERG_11986	GABA permease, putative (*A. benhamiae*)	CACGTA (rc)	867	UPRE-2
TERG_11992	hypothetical protein	TACGTG	682	UPRE-2
TERG_11993	hypothetical protein	TACGTG	387	UPRE-2
TERG_11994	hypothetical protein	TACGTG	9	UPRE-2
TERG_11998	potassium uptake transporter (*T. equinum*)	TACGTG	518	UPRE-2
TERG_11999	ubiquitin carboxyl-terminal hydrolase (*T. tonsurans*)	TACGTG	846	UPRE-2
TERG_12000	ubiquitin C-terminal hydrolase, putative (*T. verrucosum*)	TACGTG	113	UPRE-2
TERG_12007	DUF726 domain protein (*T. verrucosum*)	TACGTG	692	UPRE-2
TERG_12026	nonsense-mediated mRNA decay protein 2 (*T. tonsurans*)	CACGTA (rc)	925	UPRE-2
TERG_12030	DUF821 domain protein (*T. verrucosum*)	CACGTA (rc)	784	UPRE-2
TERG_12060	C-x8-C-x5-C-x3-H type zinc finger protein (*T. equinum*)	TACGTG	793	UPRE-2

TERG_12077	hypothetical protein	CACGTA (rc)	717	UPRE-2
TERG_12079	protein N-terminal asparagine amidohydrolase, putative (*T. verrucosum*)	TACGTG	891	UPRE-2
TERG_12121	DNA replication licensing factor mcm5 (*T. tonsurans*)	TACGTG	316	UPRE-2
TERG_12138	hypothetical protein	TACGTG	474	UPRE-2
TERG_12150	serine/threonine-protein kinase bur1	TACGTG	53	UPRE-2
TERG_12153	pumilio-family RNA binding protein (*T. tonsurans*)	CACGTA (rc)	353	UPRE-2
TERG_12157	PQ-loop repeat-containing protein 1 (*T. equinum*)	TACGTG	189	UPRE-2
TERG_12160	STE/STE11/CDC15 protein kinase (*T. tonsurans*)	TACGTG	52	UPRE-2
TERG_12181	C6 finger domain protein, putative (*T. verrucosum*)	CACGTA (rc)	920	UPRE-2
TERG_12198	hypothetical protein	CACGTA (rc)	16	UPRE-2
TERG_12203	GARP complex subunit (Sac2), putative (*T. verrucosum*)	TACGTG	382	UPRE-2
TERG_12204	RNA polymerase I specific transcription initiation factor Rrn7, putative (*T. verrucosum*)	TACGTG	526	UPRE-2
TERG_12238	integral membrane protein (*M. gypseum*)	TACGTG	264	UPRE-2
TERG_12293	hypothetical protein	TACGTG	985	UPRE-2
TERG_12301	hypothetical protein	TACGTG	873	UPRE-2
TERG_12310	null (*T. verrucosum*)	CACGTA (rc)	186	UPRE-2
TERG_12342	membrane zinc transporter (*T. tonsurans*)	TACGTG	620	UPRE-2
TERG_12365	secreted protein (*T. equinum*)	TACGTG	187	UPRE-2
TERG_12405	cap binding protein (*T. equinum*)	TACGTG	965	UPRE-2
TERG_12417	hypothetical protein	TACGTG	992	UPRE-2
TERG_12419	uracil permease (*M. gypseum*)	TACGTG	333	UPRE-2
TERG_12425	von Willebrand RING finger domain-containing protein (*T. tonsurans*)	TACGTG	811	UPRE-2
TERG_12432	hypothetical protein	TACGTG	754	UPRE-2
TERG_12441	hypothetical protein	CACGTA (rc)	515	UPRE-2
TERG_12442	hypothetical protein	CACGTA (rc)	599	UPRE-2
TERG_12459	hypothetical protein	TACGTG	409	UPRE-2
TERG_12480	ribosome biogenesis protein Pescadillo (*T. tonsurans*)	TACGTG	385	UPRE-2
TERG_12483	nonribosomal brevianamide peptide synthase FtmA (*T. verrucosum*)	TACGTG	370	UPRE-2
TERG_12485	nonribosomal brevianamide peptide synthase FtmA (*T. verrucosum*)	TACGTG	756	UPRE-2
TERG_12486	nonribosomal brevianamide peptide synthase FtmA (*A. benhamiae*)	TACGTG	204	UPRE-2
TERG_12493	averantin oxidoreductase (*T. equinum*)	TACGTG	45	UPRE-2
TERG_12513	hypothetical protein	CACGTA (rc)	667	UPRE-2
TERG_12518	phosphotransferase enzyme family protein (*T. equinum*)	TACGTG	568	UPRE-2

TERG_12579	MFS transporter (*T. tonsurans*)	TACGTG	124	UPRE-2
TERG_12590	hypothetical protein	CACGTA (rc)	889	UPRE-2
TERG_12592	hypothetical protein	TACGTG	874	UPRE-2
TERG_12593	hypothetical protein	TACGTG	451	UPRE-2
TERG_12628	hypothetical protein	CACGTA (rc)	199	UPRE-2
TERG_12633	conserved lysine-rich protein, putative (*A. benhamiae*)	CACGTA (rc)	365	UPRE-2
TERG_12635	sulfate transporter (*T. equinum*)	CACGTA (rc)	26	UPRE-2
TERG_12644	MFS transporter, putative (*A. benhamiae*)	TACGTG	825	UPRE-2
TERG_12655	hypothetical protein	TACGTG	242	UPRE-2
TERG_12687	ubiquitin ligase subunit CulD (*T. tonsurans*)	TACGTG	545	UPRE-2
TERG_12697	serine/threonine protein kinase (*T. tonsurans*)	TACGTG	113	UPRE-2
TERG_12698	serine/threonine protein kinase (*T. equinum*)	TACGTG	397	UPRE-2
TERG_00327	hypothetical protein	GTTGTCCT (rc)	985	UPRE-3
TERG_00331	golgi apparatus membrane protein TVP18 (*T. tonsurans*)	GTTGTCCT (rc)	445	UPRE-3
TERG_00377	hypothetical protein	AGGACAAC	907	UPRE-3
TERG_00568	elongator associated protein (*T. equinum*)	AGGACAAC	885	UPRE-3
TERG_00726	short-chain dehydrogenase/reductase 2, putative (*T. verrucosum*)	AGGACAAC	431	UPRE-3
TERG_00815	hypothetical protein	AGGACAAC	399	UPRE-3
TERG_00841	C6 transcription factor, putative (*T. verrucosum*)	GTTGTCCT (rc)	810	UPRE-3
TERG_00851	ankyrin repeat-containing protein (*T. equinum*)	GTTGTCCT (rc)	79	UPRE-3
TERG_00861	hypothetical protein	GTTGTCCT (rc)	897	UPRE-3
TERG_00888	hypothetical protein	GTTGTCCT (rc)	222	UPRE-3
TERG_00915	CAMK protein kinase	AGGACAAC	87	UPRE-3
TERG_01139	kinesin family protein (*T. tonsurans*)	AGGACAAC	841	UPRE-3
TERG_01280	IgE-binding protein (*A. benhamiae*)	AGGACAAC	503	UPRE-3
TERG_01329	Ctr copper transporter, putative (*T. verrucosum*)	AGGACAAC	431	UPRE-3
TERG_01447	bcp1 (*T. equinum*)	AGGACAAC	376	UPRE-3
TERG_01470	serine/threonine protein kinase (*M. canis*)	AGGACAAC	272	UPRE-3
TERG_01475	DNA topoisomerase 2	AGGACAAC	204	UPRE-3
TERG_01558	cation chloride cotransporter (*T. tonsurans*)	GTTGTCCT (rc)	185	UPRE-3
TERG_01782	hypothetical protein	GTTGTCCT (rc)	818	UPRE-3
TERG_01783	hypothetical protein	AGGACAAC	431	UPRE-3
TERG_01786	DENN domain-containing protein (*T. tonsurans*)	AGGACAAC	889	UPRE-3
TERG_01831	adenylate-forming enzyme AfeA (*A. benhamiae*)	GTTGTCCT (rc)	301	UPRE-3
TERG_01851	Pfs, NACHT and WD domain protein (*A. benhamiae*)	AGGACAAC	96	UPRE-3

TERG_02232	Rho GTPase activator (Bem2), putative (*T. verrucosum*)	GTTGTCCT (rc)	384	UPRE-3
TERG_02363	succinate-semialdehyde dehydrogenase (*T. tonsurans*)	AGGACAAC	982	UPRE-3
TERG_02383	mitochondrial carrier protein (*T. tonsurans*)	AGGACAAC	72	UPRE-3
TERG_02421	urea transporter (*M. canis*)	GTTGTCCT (rc)	468	UPRE-3
TERG_02438	hypothetical protein	GTTGTCCT (rc)	737	UPRE-3
TERG_02439	palmitoyltransferase (*T. tonsurans*)	AGGACAAC	715	UPRE-3
TERG_02624	hypothetical protein	GTTGTCCT (rc)	371	UPRE-3
TERG_02772	DNA polymerase V (*T. tonsurans*)	GTTGTCCT (rc)	920	UPRE-3
TERG_02785	hypothetical protein	GTTGTCCT (rc)	581	UPRE-3
TERG_02870	pre-mRNA-splicing factor ATP-dependent RNA helicase prp22 (*T. equinum*)	GTTGTCCT (rc)	637	UPRE-3
TERG_02871	CUE domain-containing protein (*T. tonsurans*)	AGGACAAC	907	UPRE-3
TERG_03063	hypothetical protein	GTTGTCCT (rc)	322	UPRE-3
TERG_03064	MCAK-like kinesin (*T. equinum*)	AGGACAAC	363	UPRE-3
TERG_03114	T-complex protein 1 subunit gamma	AGGACAAC	336	UPRE-3
TERG_03222	DUF895 domain membrane protein (*T. verrucosum*)	GTTGTCCT (rc)	488	UPRE-3
TERG_03388	hypothetical protein	AGGACAAC	160	UPRE-3
TERG_03481	hypothetical protein	AGGACAAC	895	UPRE-3
TERG_03486	Lectin C-type domain protein (*T. verrucosum*)	GTTGTCCT (rc)	397	UPRE-3
TERG_03503	ULK protein kinase (*T. tonsurans*)	GTTGTCCT (rc)	31	UPRE-3
TERG_03587	WD repeat protein (*T. verrucosum*)	AGGACAAC	531	UPRE-3
TERG_03636	ankyrin repeat protein (*T. equinum*)	GTTGTCCT (rc)	66	UPRE-3
TERG_03650	hypothetical protein	AGGACAAC	267	UPRE-3
TERG_03682	hypothetical protein	AGGACAAC	470	UPRE-3
TERG_03745	ankyrin repeat-containing protein (*T. equinum*)	GTTGTCCT (rc)	427	UPRE-3
TERG_03797	hypothetical protein	GTTGTCCT (rc)	215	UPRE-3
TERG_03854	laccase (*T. tonsurans*)	AGGACAAC	387	UPRE-3
TERG_03898	GTP cyclohydrolase II	AGGACAAC	562	UPRE-3
TERG_04185	calcium channel subunit Mid1 (*T. verrucosum*)	AGGACAAC	767	UPRE-3
TERG_04339	signal transduction protein Syg1, putative (*A. benhamiae*)	GTTGTCCT (rc)	121	UPRE-3
TERG_04444	hypothetical protein	GTTGTCCT (rc)	36	UPRE-3
TERG_04486	ER lumen protein retaining receptor (*T. tonsurans*)	AGGACAAC	495	UPRE-3
TERG_04686	porphobilinogen synthase (*T. tonsurans*)	AGGACAAC	443	UPRE-3
TERG_04724	hypothetical protein	AGGACAAC	661	UPRE-3
TERG_04873	autophagy protein Apg5, putative (*A. benhamiae*)	AGGACAAC	308	UPRE-3
TERG_04946	rho GTPase ModA (*T. tonsurans*)	GTTGTCCT (rc)	127	UPRE-3
TERG_05047	hypothetical protein	GTTGTCCT (rc)	344	UPRE-3

TERG_05048	U2 small nuclear ribonucleoprotein A'	AGGACAAC	700	UPRE-3
TERG_05062	ribonuclease P complex subunit Pop4 (*T. tonsurans*)	GTTGTCCT (rc)	94	UPRE-3
TERG_05257	integral membrane protein (*T. tonsurans*)	AGGACAAC	601	UPRE-3
TERG_05363	hypothetical protein	AGGACAAC	902	UPRE-3
TERG_05366	hypothetical protein	AGGACAAC	772	UPRE-3
TERG_05628	GPI ethanolamine phosphate transferase (*T. tonsurans*)	GTTGTCCT (rc)	826	UPRE-3
TERG_05700	hypothetical protein	AGGACAAC	38	UPRE-3
TERG_05776	peroxisomal ABC transporter (*T. tonsurans*)	AGGACAAC	807	UPRE-3
TERG_05908	hypothetical protein	GTTGTCCT (rc)	147	UPRE-3
TERG_05956	rRNA processing protein Ebp2p (*T. equinum*)	AGGACAAC	709	UPRE-3
TERG_06026	ribosomal protein S24 (*T. tonsurans*)	AGGACAAC	118	UPRE-3
TERG_06057	C2 domain-containing protein (*T. equinum*)	GTTGTCCT (rc)	408	UPRE-3
TERG_06118	hypothetical protein	GTTGTCCT (rc)	759	UPRE-3
TERG_06250	alpha 1,6 mannosyltransferase (*M. canis*)	GTTGTCCT (rc)	849	UPRE-3
TERG_06251	hypothetical protein	AGGACAAC	276	UPRE-3
TERG_06269	hypothetical protein	GTTGTCCT (rc)	534	UPRE-3
TERG_06368	hypothetical protein	GTTGTCCT (rc)	249	UPRE-3
TERG_06369	PQ loop repeat protein (*A. benhamiae*)	AGGACAAC	292	UPRE-3
TERG_06379	transcription factor RfeF (*T. tonsurans*)	AGGACAAC	858	UPRE-3
TERG_06506	hypothetical protein	GTTGTCCT (rc)	615	UPRE-3
TERG_06507	oxidoreductase, 2OG-Fe(II) oxygenase family (*T. verrucosum*)	AGGACAAC	61	UPRE-3
TERG_06507	oxidoreductase, 2OG-Fe(II) oxygenase family (*T. verrucosum*)	AGGACAAC	649	UPRE-3
TERG_06577	37S ribosomal protein S9 (*T. verrucosum*)	GTTGTCCT (rc)	747	UPRE-3
TERG_06658	hypothetical protein	AGGACAAC	340	UPRE-3
TERG_06685	riboflavin aldehyde-forming enzyme (*T. tonsurans*)	GTTGTCCT (rc)	265	UPRE-3
TERG_06728	hypothetical protein	AGGACAAC	670	UPRE-3
TERG_06768	pre-mRNA-splicing ATP-dependent RNA helicase PRP28	GTTGTCCT (rc)	86	UPRE-3
TERG_06769	hypothetical protein	GTTGTCCT (rc)	798	UPRE-3
TERG_07077	C6 transcription factor (*T. equinum*)	AGGACAAC	315	UPRE-3
TERG_07085	peptidase (*T. tonsurans*)	GTTGTCCT (rc)	819	UPRE-3
TERG_07159	geranylgeranyl transferae type II alpha subunit, putative (*T. verrucosum*)	GTTGTCCT (rc)	547	UPRE-3
TERG_07163	GTPase activating protein (*T. tonsurans*)	AGGACAAC	938	UPRE-3
TERG_07168	histone acetylase complex subunit (*T. tonsurans*)	AGGACAAC	813	UPRE-3
TERG_07195	AAA family ATPase (*T. tonsurans*)	AGGACAAC	982	UPRE-3

TERG_07233	acriflavine sensitivity control protein acr-2 (*T. equinum*)	GTTGTCCT (rc)	559	UPRE-3
TERG_07234	hypothetical protein	AGGACAAC	393	UPRE-3
TERG_07253	F-box and WD repeat-containing protein (*T. tonsurans*)	GTTGTCCT (rc)	112	UPRE-3
TERG_07331	integral membrane protein (*T. tonsurans*)	GTTGTCCT (rc)	143	UPRE-3
TERG_07433	hypothetical protein	AGGACAAC	668	UPRE-3
TERG_07435	hypothetical protein	AGGACAAC	397	UPRE-3
TERG_07446	hypothetical protein	AGGACAAC	685	UPRE-3
TERG_07469	SUN domain-containing protein (*T. equinum*)	GTTGTCCT (rc)	270	UPRE-3
TERG_07472	DNA repair helicase rad15	AGGACAAC	671	UPRE-3
TERG_07479	hypothetical protein	GTTGTCCT (rc)	793	UPRE-3
TERG_07485	UDP-galactopyranose mutase (*T. equinum*)	AGGACAAC	279	UPRE-3
TERG_07636	dual specificity phosphatase (*T. tonsurans*)	GTTGTCCT (rc)	361	UPRE-3
TERG_07698	CHL4 family chromosome segregation protein, putative (*T. verrucosum*)	AGGACAAC	235	UPRE-3
TERG_07844	HAL protein kinase	GTTGTCCT (rc)	584	UPRE-3
TERG_07932	vesicle-mediated transport protein Vid24, putative (*T. verrucosum*)	GTTGTCCT (rc)	524	UPRE-3
TERG_07983	hypothetical protein	GTTGTCCT (rc)	236	UPRE-3
TERG_08013	Rox3 mediator complex subunit (*T. equinum*)	GTTGTCCT (rc)	519	UPRE-3
TERG_08017	glutamyl-tRNA synthetase (*T. tonsurans*)	AGGACAAC	505	UPRE-3
TERG_08031	PHD finger domain-containing protein (*T. equinum*)	GTTGTCCT (rc)	210	UPRE-3
TERG_08153	hypothetical protein	GTTGTCCT (rc)	547	UPRE-3
TERG_08191	glucooligosaccharide oxidase (*T. equinum*)	GTTGTCCT (rc)	969	UPRE-3
TERG_08208	glutathione S-transferase (*T. verrucosum*)	AGGACAAC	832	UPRE-3
TERG_08282	hypothetical protein	GTTGTCCT (rc)	222	UPRE-3
TERG_08289	Rpp14 family protein (*T. equinum*)	AGGACAAC	343	UPRE-3
TERG_08316	aflatoxin efflux pump (*M. gypseum*)	AGGACAAC	509	UPRE-3
TERG_08319	CMGC/SRPK protein kinase	GTTGTCCT (rc)	738	UPRE-3
TERG_08320	tetratricopeptide repeat protein 1 (*T. equinum*)	AGGACAAC	940	UPRE-3
TERG_08421	pH signal transduction protein PalI, putative (*T. verrucosum*)	GTTGTCCT (rc)	548	UPRE-3
TERG_08422	hypothetical protein	AGGACAAC	224	UPRE-3
TERG_08503	secalin (*M. canis*)	AGGACAAC	384	UPRE-3
TERG_08508	hypothetical protein	AGGACAAC	956	UPRE-3
TERG_08521	Ser/Thr protein phosphatase (*T. equinum*)	AGGACAAC	885	UPRE-3
TERG_08532	DNA-binding protein HGH1, putative (*T. verrucosum*)	AGGACAAC	968	UPRE-3
TERG_08708	SesA protein (*T. equinum*)	GTTGTCCT (rc)	246	UPRE-3
TERG_11527	tenascin C (*T. equinum*)	AGGACAAC	116	UPRE-3

TERG_11614	hypothetical protein	AGGACAAC	301	UPRE-3
TERG_11646	hypothetical protein	AGGACAAC	506	UPRE-3
TERG_11827	DUF323 domain-containing protein (*T. equinum*)	AGGACAAC	930	UPRE-3
TERG_11831	hypothetical protein	GTTGTCCT (rc)	428	UPRE-3
TERG_11834	hypothetical protein	GTTGTCCT (rc)	808	UPRE-3
TERG_11962	DEAD/DEAH box RNA helicase (*T. tonsurans*)	AGGACAAC	369	UPRE-3
TERG_12004	lipase (*T. tonsurans*)	GTTGTCCT (rc)	752	UPRE-3
TERG_12152	hypothetical protein	AGGACAAC	827	UPRE-3
TERG_12211	ribosome biogenesis protein (*T. tonsurans*)	AGGACAAC	340	UPRE-3
TERG_12239	integral membrane protein (*M. gypseum*)	AGGACAAC	972	UPRE-3
TERG_12342	membrane zinc transporter (*T. tonsurans*)	AGGACAAC	871	UPRE-3
TERG_12432	hypothetical protein	AGGACAAC	423	UPRE-3
TERG_12477	ABC bile acid transporter, putative (*T. verrucosum*)	GTTGTCCT (rc)	592	UPRE-3
TERG_12622	hypothetical protein	GTTGTCCT (rc)	140	UPRE-3
TERG_12663	hypothetical protein	AGGACAAC	969	UPRE-3

DNA sequences upstream (1,000-bp)
Number of unique genes (UPRE-1=1,518; UPRE-2=678; UPRE-3=142)
Overall number of unique genes=2,178
rc: reverse complement
